# Regulation of gene expression in trypanosomatids: living with polycistronic transcription

**DOI:** 10.1098/rsob.190072

**Published:** 2019-06-05

**Authors:** Christine Clayton

**Affiliations:** University of Heidelberg Center for Molecular Biology (ZMBH), Im Neuenheimer Feld 282, D69120 Heidelberg, Germany

**Keywords:** *Trypanosoma*, *Leishmania*, mRNA decay, mRNA translation, mRNA processing, transcription

## Abstract

In trypanosomes, RNA polymerase II transcription is polycistronic and individual mRNAs are excised by *trans*-splicing and polyadenylation. The lack of individual gene transcription control is compensated by control of mRNA processing, translation and degradation. Although the basic mechanisms of mRNA decay and translation are evolutionarily conserved, there are also unique aspects, such as the existence of six cap-binding translation initiation factor homologues, a novel decapping enzyme and an mRNA stabilizing complex that is recruited by RNA-binding proteins. High-throughput analyses have identified nearly a hundred regulatory mRNA-binding proteins, making trypanosomes valuable as a model system to investigate post-transcriptional regulation.

## 1. Introduction

In trypanosomatids, initiation of transcription by RNA polymerase II is not controlled at the level of individual genes. Instead, there is regulation of mRNA processing, translation and decay. Trypanosomatids that are easily cultured and genetically manipulated are, therefore, excellent models for the study of post-transcriptional control. The supergroup to which trypanosomatids belong, Excavata, diverged very early in eukaryotic evolution from the supergroups that include mammals, fungi and plants [1] (electronic supplementary material, Note 1 in Supplementary Notes). Because of this, comparisons between the different models enable us to distinguish characteristics that were present in the last eukaryotic common ancestor from those that evolved later [2]. Moreover, one aspect of trypanosome gene expression—their mRNA processing—is a target of clinically useful drugs for the treatment of human and ruminant trypanosomiasis [3,4].

In this review, I summarize our knowledge of kinetoplastid RNA polymerase II transcription, mRNA processing and export, then I describe regulation of mRNA translation and mRNA degradation in more detail. The electronic supplementary material, Notes, provides extra information and references on particular topics, while electronic supplementary material, table S1, lists many of the proteins involved. The table includes details of all currently known mRNA-binding proteins, and other proteins that have been implicated in post-transcriptional RNA control.

Trypanosomatida are intracellular or extracellular parasites of plants and/or animals [5]. All have a single flagellum and a complex of circular mitochondrial DNAs called the kinetoplast. Trypanosomatid parasites of medical, veterinary or agricultural importance are usually transmitted by arthropods; they include plant parasites of the genus *Phytomonas*, and *Leishmania* and *Trypanosoma* species, which cause diseases in vertebrates.

**Figure 1.**
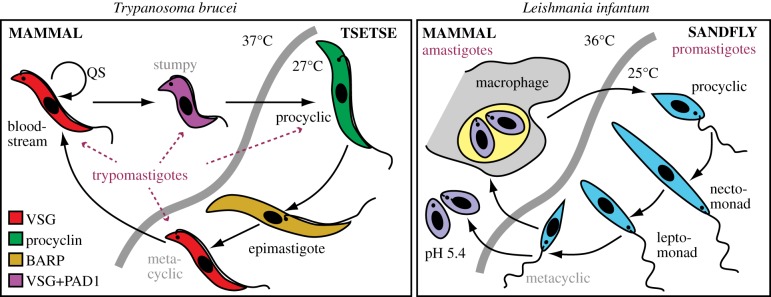
Life cycles of *T. brucei* and *Leishmania*. These simplified diagrams show the main developmental stages mentioned in this review. Different surface molecules are colour coded. The different stages are named according to the presence or absence of an external flagellum, overall shape and the position of the kinetoplast relative to the nucleus. In trypomastigotes (dotted arrows), the kinetoplast and flagellar base are posterior to the nucleus; in promastigotes, they are anterior. Amastigotes are amotile and have only a flagellar stub. Non-dividing transmission forms are labelled in grey and the macrophage lumen is pale yellow; QS means quorum sensing. Transformation of bloodstream-form trypanosomes to procyclic forms can be induced *in vitro* by applying specific stimuli, such as *cis*-aconitate, and changing the culture conditions (providing proline, decreasing the temperature). (*b*) Transformation of *Leishmania* metacyclic promastigotes to axenic amastigote-like forms can be achieved by elevating the temperature and the medium acidity. Culture temperatures for amastigotes are species dependent.

Gene expression has been studied most extensively in the African trypanosome *Trypanosoma brucei*, which will, therefore, be the main focus of this review. *T. brucei* live extracellularly in the blood and tissue fluids of mammals, and in the digestive system of tsetse flies. Within mammals, the long slender bloodstream-form trypomastigotes evade humoral immunity by antigenic variation of the variant surface glycoprotein (VSG) coat [6]. Bloodstream forms generate ATP from substrate-level phosphorylation during glycolysis (electronic supplementary material, Note 2). When they attain a sufficient density, oligopeptide quorum sensing [7] triggers the cells to become shorter and fatter stumpy-form trypomastigotes, with *G*_0_/*G*_1_ cell-cycle arrest and strongly downregulated transcription and translation [8] (figure 1) (electronic supplementary material, Note 2). Upon uptake by a tsetse fly, the stumpy forms differentiate into procyclic trypomastigotes, which multiply in the midgut. Procyclic forms express different surface proteins—procyclins—and rely on mitochondrial metabolism for ATP (electronic supplementary material, Note 2). Further movement of the parasites through the proventriculus to the salivary glands, differentiation to epimastigotes, a non-obligatory sexual stage, and then conversion to cell-cycle-arrested, VSG-expressing metacyclic trypomastigotes completes the cycle [9].

Leishmanias live in various vertebrates, where they grow inside modified macrophage lysosomes as amastigotes, and within the sandfly digestive system as promastigotes (figure 1). *Trypanosoma cruzi* grows as epimastigotes in reduviid bugs, converting to transmissible metacyclic forms. Within mammals, amastigotes divide in the cytosol of various cell types, while non-dividing extracellular trypomastigotes are responsible for transmission (electronic supplementary material, Note 3).

## 2. Genome organization and transcription in kinetoplastids

### 2.1. Genome organization

**Figure 2.**
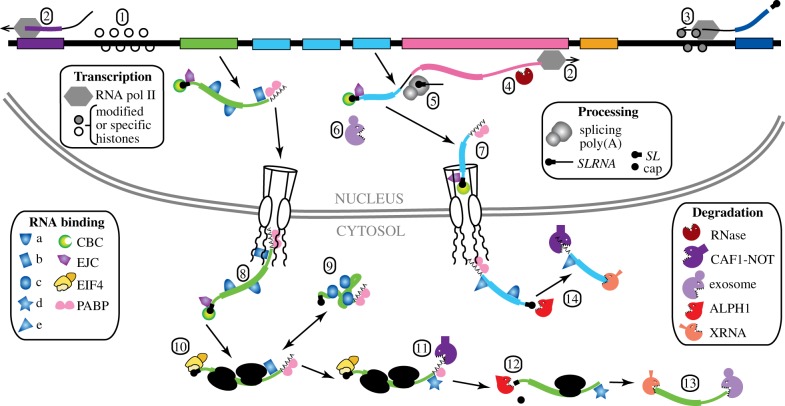
Gene expression in kinetoplastids. This diagram is modified from [11]. The thick line at the top represents the genome, and each coloured block is represented in a mature mRNA. The corresponding mRNAs are shown with the coding regions thicker than the untranslated regions. The numbered steps are described in the text. 1: Modified histones in an RNA polymerase II initiation region. 2: RNA polymerase II elongation. 3: RNA polymerase II termination. 4: Endonuclease cleavage of precursor (postulated). 5: *Trans*-splicing and polyadenylation. 6: Incompletely processed mRNAs can be degraded by the exosome. 7: Export of a completed mRNA, with bound poly(A) binding protein (PABP), exon junction complex (EJC) and nuclear cap-binding complex (CBC). (Note that we do not know which, if any, splice junctions are bound by the EJC, and also that mRNA export can commence before the 3′-end is complete.) 8: Emergence of a mature mRNA including proteins on the coding region (a) and a specific, stabilizing protein (b) bound to the 3′-untranslated region (3′-UTR). 9: Binding by a silencing or aggregating RNA-binding protein (c) and condensation into granules. 10: Binding of EIF4E, EIF4G and EIF4A, and translation. 11: Protein (b) is replaced by a destabilizing RNA-binding protein (d) and deadenylation starts. 12: Decapping by ALPH1. 13: Degradation by XRNA and the exosome. 14: Rapid decay pathway—immediate decapping promoted by protein (e). Many of the proteins involved are listed in electronic supplementary material, table S1.

All Kinetoplastea genomes that have been studied to date have similar overall structures [10,11]. Multiple intron-less open reading frames are arranged in a head-to-tail fashion, sometimes with tandem repeats, and with only occasional changes in direction (figure 2). Most RNA polymerase II transcription initiates in DNA stretches of several kilobases that separate gene arrays orientated in opposite, diverging directions (figure 2(1)). Transcription units can be over 100 kb long. Although the gene order is partially evolutionarily conserved [12], individual transcription units contain genes that are unrelated with regard to both mRNA expression and the functions of the encoded proteins. Termination occurs either where two transcription units converge (figure 2(3)) or at regions transcribed by other polymerases (e.g. tRNA and rRNA genes). The primary transcript is processed into individual mRNAs by *trans*-splicing of a capped ‘spliced leader’ (*SL*) [13], and by polyadenylation [14] (figure 2(4)). *T. brucei* has only two verified *cis*-spliced genes, and none has been confirmed for other kinetoplastids (electronic supplementary material, Note 4). To compensate for the lack of ‘activatable’ polymerase II transcription, genes encoding abundant mRNAs are often present in multiple copies (illustrated in cyan in figure 2) (electronic supplementary material, Note 5).

In *T. brucei*, the telomeres carry long arrays of *VSG* genes and pseudogenes [15]. The active *VSG* gene is transcribed by RNA polymerase I from one of several alternative expression sites, most of which contain other genes upstream of the *VSG* gene. Antigenic switching can occur either by a transcriptional switch to use of an alternative expression site and promoter, or by recombination or gene conversion events that change the *VSG* sequence within the currently active expression site [6].

### 2.2. Transcription initiation by RNA polymerase II

Trypanosome RNA polymerase II has 12 subunits, which are homologues of those from other eukaryotes. The basal polymerase II transcription factors are more divergent from those of opisthokonts, and include both conserved and novel proteins [16,17]. They include TBP (originally called TRF4) [18], TFIIB [19], the small TFIIA subunit and SNAPc [16], TFIIH [20], Mediator complex [21], a PAF complex [22] and two kinetoplastid-specific proteins that may form the equivalent of TFIIF [17,19]. The general transcription factors are required for *SLRNA* transcription *in vitro*, and it seems very likely that they are needed for polycistronic transcription of protein-coding genes too, although in most cases, this has not been tested. Chromatin immunoprecipitation (ChIP-Seq) experiments should resolve this issue.

In opisthokonts, the repetitive C-terminal domain (CTD) of the largest subunit of RNA polymerase II is phosphorylated; and in humans, this is effected by the CDK7 kinase component of TFIIH [23]. Although the *T. brucei* largest pol II subunit is phosphorylated [24] and the C-terminus is required for RNA synthesis [25,26], it lacks repeats [27]. *T. brucei* TFIIH also has no associated kinase and did not co-purify with RNA polymerase II [28] (electronic supplementary material, Note 6).

Transcription start sites (figure 2(1)) for mRNAs were mapped by purification and sequencing of 5′-triphosphate RNAs. Most start sites are scattered throughout regions that separate divergent transcription units, with clear overlaps between the initiation sites for each direction; some start sites instead allow for re-initiation without a direction change [29,30]. Initiation regions lack open reading frames, have open chromatin [30] and are enriched in H4K10ac, H3K4ac, the histone variants H2AZ, H2BV, the bromodomain factor BDF3 [31,32] and RNA–DNA hybrids [33]. Although several results indicate that polymerase II initiation can be stimulated merely by the existence of open chromatin [34–36], initiation is favoured by GT-rich sequences [30] (electronic supplementary material, Note 7). It is not known how these sequences are recognized by chromatin modifiers. Only two, rather minor differences in histone variant distribution were observed between life cycle stages [31]. The significance of the differences is unclear, since the parasites used had been cultured separately for many years. It would be useful to find out whether differences are also seen for trypanosomes that have recently differentiated. Results from nuclear run-on experiments (e.g. [37]), as well as results from modelling [38], indicate that transcription elongation (figure 2(2)) is usually constitutive. New results from the sequencing of 5-ethynyl uridine-labelled RNA should provide much more detail about transcription initiation and elongation [39].

The ‘retroposon hotspot’ (RHS) proteins of *T. brucei* [40] fall into six groups, of which all but RHS6 can be cross-linked to mRNA [41]. By ChIP-Seq, RHS2, RHS4 and RHS6 all colocalize with RNA polymerase II, and RHS6 proteins are directly associated with chromatin [39]. RHS4 and RHS6 proteins are also associated with several other proteins implicated in transcription, whereas RHS2 associates with ribosomes and mRNA-binding proteins [39]. Depletion of any of these proteins resulted in a decrease in transcription, as judged by 5-ethynyl uridine incorporation [39]. These proteins are present only in *Trypanosoma*; it is not known whether equivalent proteins exist in other kinetoplastids.

The 142 nt spliced leader precursor RNA, *SLRNA*, is the only polymerase II transcript that arises from genes with discrete promoters and terminators [42]. The high *SLRNA* synthesis rate required for *trans*-splicing is attained in part through the presence of about 100 *SLRNA* gene copies arranged as tandem repeats [43]. In *T. cruzi*, this arrangement results in concentration of RNA polymerase II at a specific position within the nucleus [44]. Although *T. cruzi* is at least diploid, only one location is usually seen, perhaps suggesting contact between homologous chromosomes.

Despite the high gene copy number initiation of *SLRNA* transcription has to be much more efficient than at the divergence regions: in bloodstream form *T. brucei*, every *SLRNA* gene needs to make about 140 mRNAs per hour, in contrast to protein-coding genes which are probably transcribed only once or twice per hour [45]. Nucleosomes are strongly phased over the *T. brucei SLRNA* genes, with peaks at the start and termination sites [46]. H2AZ is present, but at a lower level than on the polycistronic unit divergence regions [30]. *SLRNA* promoter recognition and transcription can be studied in an *in vitro* system, and were shown to require a specific initiation complex which contains TBP, distant homologues of human SNAP50 and SNAP43, and a unique component [16,42]. Endoplasmic reticulum stress triggers phosphorylation of TBP and silencing of *SLRNA* transcription, ultimately leading to programmed cell death [47,48].

### 2.3. Polymerase II termination

In opisthokonts, RNA polymerase II termination is linked to polyadenylation. Poly(A) site cleavage seems to suffice for termination, but termination efficiency is enhanced by the nuclear 5′→3′ exoribonuclease Rat1/Xrn2, which is thought to digest the remnant pre-RNA precursor until it catches up with the polymerase [49]. This polyadenylation-linked ‘torpedo’ model is not possible in an organism with polycistronic transcription.

Kinetoplastid RNA polymerase II transcription terminates at convergence regions (figure 2(3)), just upstream of tRNA genes [31,35] or pol I-transcribed genes (electronic supplementary material, Note 8), and upstream of the *SLRNA* array [50]. *T. brucei* polymerase II termination regions are enriched with variant histones H3V and H4V [31,50] and cohesin [15]. Histones H3V and H4V are also enriched near telomeres, which have compacted chromatin and are not transcribed by polymerase II [15].

In addition to the two variant histones, a specific modification called base J (beta-D-glucosyl-hydroxymethyluracil) [51] is involved in termination, but the details are species specific. Base J plays a role in termination in bloodstream-form *T. brucei* [50,52,53] and *Leishmania* [54–56] but not in procyclic *T. brucei*, where it is absent [57]. In *T. cruzi*, base J may influence initiation as well [58].

The detailed mechanism of polymerase II termination has not been investigated. Intriguingly, the essential nuclear RNA-binding protein RPB33 binds preferentially to RNAs from regions where polymerase II transcription units converge, and its loss leads to a considerable increase in RNAs that originate from the wrong strand, as well as retroposon and repeat RNAs [59]. These results implicate RBP33 in termination. How RBP33 is recruited to nascent RNAs from termination regions, or targets them for destruction, is not known (electronic supplementary material, Note 9). Kinetoplastids do have a nuclear Rat1/Xrn2 homologue, XRND, which is essential [60], but its detailed function has not been investigated.

In *Leishmania tarentolae*, *SLRNA* transcription terminates just downstream of the mature 3′-end, within an oligo d(T) stretch that is important for, but not the sole determinant of, termination [61]. Oligo d(T) tracts are found downstream of *SLRNA* genes in other trypanosomatids as well. After termination, the mature *SLRNA* is trimmed, probably by a 3′→5′ exoribonuclease that is also required for processing other small RNAs [62]. The mechanism of *SLRNA* transcription termination is clearly different from that for mRNAs, since oligo d(T) tracts are scattered throughout the DNA that specifies mRNA 3′-untranslated regions (UTRs) and intergenic regions.

### 2.4. RNA polymerase I transcription of protein-coding genes

VSG expression sites and the procyclin loci are transcribed by RNA polymerase I [63], with expression in bloodstream and procyclic forms, respectively [64]. This review concentrates on regulation of genes that are transcribed by RNA polymerase II so evidence concerning VSG and procyclin expression will be summarized only extremely briefly.

*VSG,* procyclin and *rRNA* genes have discrete promoter sequences with clear—but completely different—consensus sequences. So far, no transcription factor that binds just one of these promoter types has been found [65]. Use of polymerase I to make *VSG* mRNA is necessary in order to obtain adequate mRNA levels from the single active *VSG* gene [66]: transcription of a gene from a polymerase I promoter can give at least 10 times more RNA than read-through transcription by RNA polymerase II [64]. The nature of the associated histones may affect this, since the transcription efficiency is affected by the location of the promoter in the genome [30,64,67–70]. The mechanisms of *VSG* and procyclin gene promoter developmental regulation are not understood, but chromosomal location and epigenetic effects are implicated.

The complex mechanism by which all but one *VSG* expression site is suppressed in bloodstream forms has been studied in considerable detail. Very briefly, chromatin structure [15,67,71], sub-nuclear localization [72] and transcription elongation [73], rather than the precise promoter sequence [74], appear to be important. For further information and references to the very extensive literature concerning VSG transcription and antigenic variation, see [15,33,75,76].

## 3. mRNA processing and export

### 3.1. The 5′ cap and *SLRNA* processing

Mature mRNAs acquire their cap via *trans*-splicing, and all have the same 39 nt at the 5′-end (electronic supplementary material, Note 10). The structure is m^7^G(5′)ppp(5′)m^6^_2_AmpAmpCmpm^3^Ump, with 2′-O ribose methylation of the first four transcribed residues [77,78], as well as N^6^,N^6^,2′-O-trimethyladenosine and 3,2′-O-dimethyluridine [79]. Although the guanylyl transferase [80] and enzymes responsible for ribose methylation [81,82] are known, those responsible for base methylation are not [83]. Cap methylation is required for *trans*-splicing [84], and the ribose methylation is needed for efficient translation [82]. *SLRNA* is also pseudouridylated, although this is not required for function [85].

Capping and methylation of *SLRNA* are co-transcriptional [86], as for polymerase II transcripts in other eukaryotes. In opisthokonts, the capping enzyme is associated with RNA polymerase II via interactions with both the body of the enzyme and the CTD. Presumably, the capping machinery is also associated with the *SLRNA* transcription complex in trypanosomes, but how this occurs is not known. If indeed capping is specific to the *SLRNA*, involvement of promoter-specific transcription factors seems possible. Depletion of the kinase CRK9, which is required for CTD phosphorylation, impaired cap methylation, but did not impair either capping itself or (surprisingly) *SLRNA* transcription [87]. Interestingly, depletion of a PRP19 complex component, which inhibits splicing (see below), resulted in similar accumulation of unmethylated *SLRNA* [88]. These observations might indicate feedback control of *SLRNA* cap methylation. However, they might also reflect growth arrest, or a first step in *SLRNA* disposal: if assembly of Sm-containing particles is inhibited by depletion of an Sm protein, *SLRNA* cannot be properly assembled as a small nuclear ribonucleoprotein particle (snRNP). Instead, unmethylated *SLRNA* accumulates, first in the nucleus but then in the cytoplasm [89].

### 3.2. Splicing and polyadenylation: basal machineries and signals

*Trans*-splicing was first discovered in trypanosomes [90,91], but was later found in many other very diverse organisms [92] (electronic supplementary material, Note 11). Trypanosomatid *trans*-splicing is mechanistically similar to *cis*-splicing, with U2, U4, U5 and U6 snRNPs; but the *SLRNA* snRNP takes the place of the U1 snRNP. The components of the spliceosomal snRNPs are broadly similar to those of other eukaryotes, with Sm core proteins [93,94] and specific components [13,95,96]. The U snRNPs undergo SMN-dependent assembly in the nucleoplasm [97,98]. A minimal U1 snRNP is implicated in *cis*-splicing of two transcripts [99]; whether it is also involved in *trans*-splicing is unclear [94,100]. The PRP19 complex [88] is required for both *cis-* and *trans*-splicing [94].

**Figure 3.**
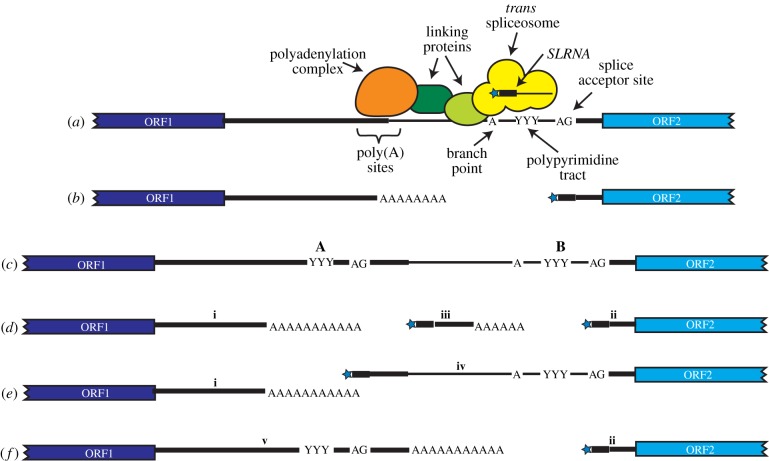
mRNA processing. (*a*) This shows the region between two different open reading frames, ORF1 and ORF2. The distance between the polypyrimidine tract and polyadenylation site in *T. brucei* is about 100 nt. The nature of the interaction between the polyadenylation and splicing complexes is unknown; hypothetical linking proteins are shown but the interaction might instead be direct. (*b*) The simplest possibility for processing the primary transcript in (*a*), when only a single splicing signal is present. (*c*) This is a precursor with two possible splicing signals, labelled as A and B. If neither signal is used, the mRNA will contain more than one open reading frame, and only protein (1) will be produced. (*d*) If both sites from precursor (*c*) are used, the RNA from ORF1 (i) has a short 3′-UTR, RNA (2) has a short 5′-UTR (ii) and there will be an additional processed RNA with no open reading frame from intergenic region (iii). This pattern has been documented in detail for the procyclin [101,102] mRNAs but is doubtless seen in many more [29]. (*e*) If only site A of precursor (*c*) is used, the RNA for ORF2 will have a long 5′-UTR (iv) that includes splicing signal (*b*). (*f*) If only the distal site B f precursor (*c*) is used, the ORF1 mRNA will have a long 3′-UTR (v) which includes polypyrimidine tract (*a*).

Like 3′ *cis* splice acceptor sites in other organisms, *trans*-splicing sites are usually preceded by a polypyrimidine tract (figure 3*a*) [29,103]. There is a preference for U over C in *T. brucei* but not in *Leishmania major* [104,105]. Longer polypyrimidine tracts give more efficient processing [69,106] and the first downstream AG is the preferred 3′ acceptor [29,107]. During *trans*-splicing, a branched Y-structure intermediate is formed when the *SLRNA* intron is 2′-5′ joined to an A residue in the intergenic region upstream of the polypyrimidine tract: this is equivalent to the lariat that is formed during *cis*-splicing. There is no consensus branch point sequence; evidence from a few genes suggests use of the A residues that are nearest to the polypyrimidine tract (figure 3*a*) [107–109] (electronic supplementary material, Note 12).

Two genes encode potential poly(A) polymerases in *T. brucei*. One mRNA is *cis* spliced, and its product is implicated in small nucleolar RNA (snoRNA) processing [110]. The other, which has no intron, encodes the major mRNA poly(A) polymerase [111]. The polyadenylation complex is conventional apart from two subunits that do not have obvious homologues in unrelated eukaryotes [111]. Polyadenylation site specification is, by contrast, not conventional: instead of there being a dedicated polyadenylation signal, the processing apparatus ‘measures’ a species-specific distance upstream from the active polypyrimidine tract [14,29,105,112] (figures 2(5) and 3*a*). The only distinguishing feature of the cleavage site itself is a preference for strings of A residues (electronic supplementary material, Note 13). The ‘measuring’ mechanism usually results in the use of a cluster of alternative polyadenylation sites. The nature of the link between the splicing and polyadenylation machineries (figure 3*a*) is not known. Rather oddly, with the exception of one study of AG acceptor dinucleotides [107], there have so far been no studies of trypanosomatid mRNA processing that quantitatively examined the effects of splicing signal mutations on the abundances and processing patterns of both the *trans*-spliced and upstream polyadenylated mRNAs.

The benzoxaborole acoziborole is undergoing clinical trials for sleeping sickness treatment [113], and other benzoxaboroles are under consideration for other kinetoplastid diseases [114]. The first detectable effect after treatment of *T. brucei* with the benzoxaborole AN7973 is an increase in mRNA precursors and a decrease in the Y-structure intermediate [3], suggesting that this compound targets mRNA processing. CPSF3 (also called CPSF73) is the enzyme that cleaves the substrate at the polyadenylation site. Over-expression of CPSF3 gives moderate resistance to AN7973 [3] and to acoziborole [4]. Both compounds can be docked into the CPSF3 active site [3,4]. These results suggest that CPSF3 is a primary (though possibly not the only) target of these particular benzoxaboroles (electronic supplementary material, Note 14).

In opisthokonts and plants, there is cross-talk between splicing, RNA polymerase II elongation complexes [115] and chromatin [116] (electronic supplementary material, Note 15). Strict coupling between polymerase II and *cis*-splicing is based on the requirement for a 5′ cap on the 5′ exon. In kinetoplastids, the *SLRNA* is equivalent to the 5′ exon. mRNA precursors provide only the 3′ acceptor site, so the polymerase that makes them appears not to influence splicing—hence the ready production of mRNAs using RNA polymerase I or bacteriophage polymerases [117,118]. Kinetoplastid mRNA processing is, as in other eukaryotes, co-transcriptional [119], but interactions between the splicing machinery and the polymerase—if they are present at all—apparently do not influence splicing kinetics [118]. By the time processing occurs, the polymerase is probably roughly 1 kb downstream [38,45]. Nucleosome positioning is phased near or over processing sites, but the patterns in *T. brucei* and *Leishmania* are different [46,120], so might be influenced by nucleotide composition rather than by processing activity (electronic supplementary material, Note 16).

### 3.3. Splicing and polyadenylation: use of alternative processing sites

Trypanosomatid 3′-UTRs and intergenic regions are riddled with low-complexity sequences, which often contain polypyrimidine tracts. This can have functional consequences—for example, the use of alternative splice acceptor sites can result in different encoded proteins, and inclusion or exclusion of subcellular localization signals [121,122]. The sequences of UTRs can affect translation and mRNA turnover (see later). Figure 3*c* illustrates a relatively simply case in which there are two possible splicing signals, A and B. In figure 3*d*, both are used, making polyadenylation of the RNA with ORF1 independent of splicing of the RNA with ORF2. In figure 3*e*, only the upstream site is used, giving ORF2 mRNA an extended 5′-UTR (iv), so if there is an AUG and open reading frame between A and B, ORF2 will be very inefficiently translated. In figure 3*f*, only the downstream signal is used, and 3′-UTR (v) retains a splicing signal A, which could result in bound splicing factors being exported with the mRNA out of the nucleus. 3′-UTR (v) may also contain regulatory sequences that are absent in 3′-UTR (i). The presence of alternative splicing signals in 3′-UTRs may explain why depletion of the basal factors U2AF35, U2AF65 and SF1 affects mRNA stability as well as splicing [123] (electronic supplementary material, Note 17). Why some processing signals are preferred over others is not evident. Since alternative processing can affect mRNA abundance, the amounts of different alternative mature mRNAs do not necessarily indicate which processing sites are preferred.

Limited results from reporter experiments suggest that when there are several polypyrimidine tracts, the one that is closest to the downstream initiator AUG is usually preferred [124,125]. This might be explained by binding of specific protein factors in the 5′-UTR, at the start of the coding region or upstream of the polypyrimidine tract. As in other eukaryotes, RNA polymerase II occupancy is higher over coding regions [46]. This might mean that elongation is slowed relative to intergenic regions, which in turn might favour use of coding-region-proximal splice sites.

### 3.4. Quality control of RNA processing

If transcription, processing and mRNA export were all constitutive, the levels of mRNAs would depend solely on degradation of the processed mRNAs [38,126]. However, many mRNAs are much less abundant than expected from their half-lives [38,126], with a strong bias against longer mRNAs. Mathematical modelling showed that this could be explained by assuming that mRNA splicing competes with a nuclear quality control mechanism that attacks precursors stochastically (figure 2(4)) [38,126]. A direct test, however, failed to confirm this hypothesis [127], so the anomalous low abundance of longer mRNAs remains unexplained (electronic supplementary material, Note 18). Nevertheless, degradation of precursors and discarded intergenic regions clearly occurs [45].

The RNA exosome is a 3′→5′ exoribonuclease complex. A core structure that is responsible for substrate recognition and RNA unwinding [128] is associated with the 3′→5′ exonuclease Rrp6 and a combined 3′→5′ exonuclease and endonuclease, Rrp44 (electronic supplementary material, Note 19). In trypanosomes, as in other eukaryotes, the principal role of the exosome is probably in the nucleus, especially processing of stable RNAs such as rRNA [129,130]. The trypanosome exosome is stably associated only with a single exoribonuclease, RRP6. Kinetoplastids have RRP44, but no association of it with the exosome has been detected (electronic supplementary material, Note 20). When *trans*-splicing is impaired, less efficient processing sites can be skipped, yielding *trans*-spliced and polyadenylated mRNAs that contain more than one open reading frame, joined by the intergenic region (figure 3*c*; electronic supplementary material, Note 21). These can accumulate and escape into the cytoplasm [131]. Interestingly, depletion of the exosome resulted in accumulation of such mRNAs [130] (electronic supplementary material, Note 22). This suggests that such partially processed mRNAs may normally be targeted for destruction by the exosome (figure 2(6)). This would require endonuclease cleavage prior to 3′→5′ digestion—could RRP44 be responsible?

Many questions remain about kinetoplastid *trans*-splicing. Why are some polypyrimidine tracts favoured for splicing, while others are unused (figure 3*f*)? How do splicing kinetics affect the abundance of the upstream mRNA? And how are partially processed mRNAs recognized by the nuclear quality control machinery? Some of these issues may be resolved by studying the roles of potential splicing regulators.

### 3.5. Splicing and polyadenylation: possible specific regulators

Metazoan proteins with serine–arginine-rich (SR) domains are sequence-specific splicing regulators which mainly bind immediately downstream of exon junctions [132–134]. SR proteins are important in defining exon–intron boundaries, but may sometimes also influence polymerase II elongation. They can accompany mature mRNAs into the cytoplasm, but are mostly not associated with translating mRNAs. In addition to the basal splicing factor U2AF65, at least five kinetoplastid proteins combine one or more N-terminal RNA recognition motifs (RRMs) with C-terminal SR domains: TRRM1 (also called RRM1), RBSR1, RBSR2, RBSR3 and TSR1. All (except RBSR2, which has not been tested) are in the nucleus, although green fluorescent protein-tagged RBSR3 is in the cytoplasm as well [135].

TRRM1 is mostly in the nucleus [136] and is essential [137,138]. Its association with PTB2/DRBD4 (see below) suggests a role in splicing [138], although most splice sites were unchanged after TRRM1 depletion. The mRNAs that changed in abundance after TRRM1 depletion were mostly not the same as those that were bound to it. Longer, less abundant RNAs were more bound than shorter ones; but as noted above, length and abundance are not independent parameters. Interestingly, TRRM1 associated with some RHS proteins and nucleosome abundance increased in some regions upon TRRM1 loss [138]; whether this was a direct effect or secondary to growth inhibition is not yet clear.

RBSR1 is absent in *Leishmania*. In *T. cruzi* [139], it is associated with snoRNAs and snRNAs. A pull-down suggested association with TRRM1, RBSR2 and four other abundant RNA-binding proteins: UBP1, UBP2, ALBA3 and ALBA4. TSR1 interacts with TSR1IP, a highly polar protein [140]. Both are localized primarily in nuclear speckles. TSR1IP and TSR1 purifications contain TRRM1 and three other proteins with RNA-binding domains (electronic supplementary material, Note 23). Depletion of either resulted in an overall decrease in splicing, but microarray results revealed only minor changes in mature mRNA abundances [140].

In addition to SR proteins, several other RRM-containing proteins are localized primarily in the nucleus and have been implicated in splicing. DRBD3 (PTB1) and DRBD4 (PTB2) (electronic supplementary material, Note 24) are both essential, and were suggested as homologues of the polypyrimidine tract binding proteins of opisthokonts [141,142]. Depletion of either caused decreases in both *SLRNA* and the Y-structure intermediate. Many mRNAs showed altered abundances, but only some of the mRNA decreases could be linked to impaired splicing [142]. The authors suggested that mRNAs with C-rich polypyrimidine tracts were more strongly affected and indeed, a later study of binding sites identified a preference of DRBD3 for a C-rich motif [143].

HNRNPH/F is a hundred times more abundant in bloodstream forms than procyclic forms, but its depletion inhibited splicing in both forms. A putative binding motif was found in 5′-UTRs of some mRNAs whose splicing was particularly impaired after HNRNPH/F depletion [144]. Microarray analyses also suggested that HNRNPH/F has additional roles in regulating mRNA stability; these will be discussed later.

Many splicing factor studies so far were limited by microarray technology. Our understanding would be considerably increased by detailed mapping of RNA-binding sites. If a factor is bound near a *trans*-splice acceptor site, it is likely to affect processing, while binding elsewhere within the mature mRNA could influence mRNA export, translation or decay. We do not yet know how many of the proteins discussed above are exported bound to mRNAs (electronic supplementary material, Notes 17 and 25).

### 3.6. Export of mRNAs to the cytoplasm

The universally conserved system for mRNA export from the nucleus (figure 2(7, 8)) consists of homologues of yeast Mex67, Yra1 Sub2 and Mtr2 [145]. As expected, RNAi targeting *T. brucei* MEX67 [146], or its interaction partners MTR2 and importin 1 [147], causes accumulation of poly(A)+ RNAs in the nucleus. Unusually, kinetoplastid MEX67 has an N-terminal zinc finger domain which is essential for function and cell survival [147]. In opisthokonts, mRNA export factors are recruited to mRNAs via the SAGA, THO and TREX complexes. In most other eukaryotes, including kinetoplastids, these complexes are missing [145] and the mechanisms by which mature mRNAs are recognized for export are not known.

An *in situ* hybridization study demonstrated that in trypanosomes, mRNA export can commence before the 3′-end has been synthesized [148]—in other words, it can be co-transcriptional. When *trans*-splicing is inhibited, granules containing mRNAs accumulate on the cytosolic side of the nuclear pore [131]. The authors examined a very long mRNA—22 kb—and found that after treatment with sinefungin, a 5′-processed version that lacked a 3′-end got stuck in the nuclear pore channel [148]. This indicates that the export machinery recognizes processed 5′-ends of mRNAs, rather than the poly(A) tail. There are three obvious options for this. MEX67 might recognize the *SL* sequence (via the zinc finger?), or it might bind to the nuclear cap-binding complex or the exon junction complex.

The opisthokont nuclear cap-binding complex, CBP20/CBP80, stimulates splicing through interaction with snRNPs, and promotes mRNA export via the TREX complex [149,150]. The trypanosome *SL* cap-binding complex contains the cap-binding component CBP20, and three additional proteins that are unrelated to CBP80 [151] (electronic supplementary material, Note 26). Depletion of any CBP inhibits growth and *trans*-splicing. Poly(A) binding proteins bind to poly(A) tails in all eukaryotes examined so far [152]. *T. brucei* has two poly(A) binding proteins, PABP1 and PABP2 [153]; the association of PABP2 with CBPs [154,155] suggests that PABP2 may accompany new mRNAs from the nucleus to the cytoplasm.

The exon junction complex (EJC) [156,157] seems the most likely candidate for export recognition. In mammals, the EJC component eIF4AIII (a DEAD-box helicase) is recruited by splicing factor CWC22 and deposited 20–24 nt upstream of splice junctions [156–158]. A stable dimer of Y14 and Magoh/RBM3 [158] then binds to eIF4AIII (electronic supplementary material, Note 27). Various additional proteins interact with the EJC and facilitate mRNA export [157]. *T. brucei* Y14 is essential for normal growth and forms a stable complex with the Magoh homologue [159]. *T. brucei* also has CWC22 and a nuclear eIF4A-like helicase that is probably equivalent to eIF4AIII [160] (electronic supplementary material, Note 28). Although no association of the *T. brucei* eIF4AIII with Y14 has been detected [159], this might be because the interaction is quite transient. We do not currently know which splice junctions—if any—are bound by the kinetoplastid EJC. However, if the EJC is deposited on the *SL* at the 5′-UTR of each mRNA after *trans*-splicing (as shown in figure 2(7, 8)), it would be an excellent marker for newly processed mRNAs.

## 4. Translation

### 4.1. Cap-binding translation initiation complexes

Eukaryotic translation initiation usually commences with binding of the 5′ cap by initiation factor eIF4E (figure 2(10)). In most (but not all) organisms [161], eIF4E recruits eIF4G, which in turn recruits the helicase eiF4A1. Meanwhile, various other factors, including eIF2 and eIF3, are included during the assembly of the 43S complex, which contains a charged methionyl tRNA bound to the 40S ribosomal subunit. Phosphorylation of the alpha subunit of eIF2 prevents 43S complex assembly. Interaction of cap-associated eIF4G with eIF3 recruits the 43S complex to mRNA, forming a 48S complex. This then scans to the initiation codon, where it is joined by a 60S subunit [162–164]. An interaction between eIF4G and poly(A) binding protein can, at least transiently, circularize the mRNA, promoting re-initiation and protecting against exonucleolytic degradation [148,165,166] (electronic supplementary material, Note 29).

*T. brucei* PABP1 and PABP2 co-sediment with polysomes under normal conditions and each is essential, implying different functions [154]. Leishmanias have an additional PABP paralogue, PABP3, which is closer to PABP1 than to PABP2 [154].

**Figure 4.**
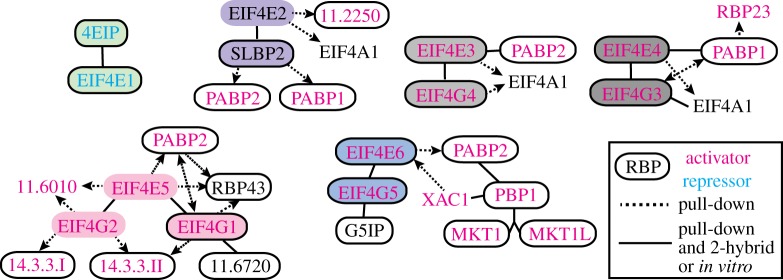
Interactions of cap-binding translation initiation complexes in *T. brucei* and *Leishmania.* The key is on the bottom right. The colours of the ovals identify different EIF4E interaction partners. Text colour codes for ‘activator’ (magenta) and ‘repressor’ (cyan) indicate whether the protein activates, or represses, gene expression when tethered within the 3′-UTR of a reporter mRNA in bloodstream-form *T. brucei* [41,167] (electronic supplementary material, Note 30). The solid frames indicate that the protein could be UV-cross-linked to poly(A)+ RNA in bloodstream-form *T. brucei* [41]. Gene numbers are from *T. brucei*, with the prefix ‘Tb927.’ removed. For results that rely on pull-down only, arrows point away from the bait protein. Suggested stage-specific interactions between *Leishmania* EIF4E1 and both EIF3 and EIF4G3 [168] are not shown. Binding activities of *Leishmania* EIF4E4 to modified caps are reported in [169,170]. Alternative names are G1-IP2 for RBP43, and G1-IP for Tb927.11.6720. References for pull-down and interaction results are as follows: *T. brucei* PABPs [155]; *Leishmania* and *T. brucei* EIF4E1 [168,171]; *T. brucei* EIF4E2 [172]; *Leishmania* EIF4G3 [173]; *T. brucei* and *Leishmania* EIF4E3 and EIF4E4 [168,174]; *T. brucei* EIF4E5 [175]; *T. brucei* EIF4E6 [176]. The tethering results for EIF4E5 and EIF4E6 are unpublished (L. Melo do Nascimento and C. Clayton 2019, unpublished data); note that the activities of these proteins when bound to the 5′ cap may not be the same as those seen in the tethering assay.

Kinetoplastids have an unusually large repertoire of eIF4Es and eIF4Gs (figure 4), which were reviewed in detail in [177]. EIF4E3, EIF4E4, EIF4E5 and EIF4E6 appear to be ‘classical’ EIF4Es, since each interacts with at least one EIF4G (figure 4). They also activate expression when tethered to the 3′-end of a reporter mRNA, suggesting that they are likely to be active in translation initiation. (For details of this assay see electronic supplementary material, Note 30.) Both EIF4E3 and EIF4E4 have unusual N-terminal extensions, and are about 100 times more abundant than EIF4E2 and EIF4E1 [174] so would suffice for translation of all mRNAs [178]. RNAi results, combined with failed knock-out attempts, indicate that the EIF4E3- and EIF4E4-based complexes are essential for general translation and cell survival [174]. Interestingly, the N-terminal extension of *Leishmania* EIF4E4 associates directly with PABP1 [179], while the C-terminal conserved domain is responsible for recruiting EIF4G3. Whether EIF4G3 also interacts directly with PABP1 is controversial [168,179,180]. EIF4E3–EIF4G4 can associate with both PABPs [155]. It is not yet known whether these complexes target different mRNAs. *Leishmania* EIF4E4 may be active only in promastigotes: its expression is downregulated in axenic amastigotes, where no EIF4E4 cap binding is detected [168,173].

EIF4E5 has two alternative EIF4G partners, EIF4G1 and EIF4G2 (figure 4) [175]; of these, EIF4G1 interacts with an RRM-domain protein (RBP43), with a protein that may modify or repair the 5′ cap [175], and with PABP2 [154,155]. *EIF4E5* RNAi in procyclics caused a motility defect [175]. Quantitative mass spectrometry results suggest that EIF4E6 is at least as abundant as EIF4E3 and EIF4E4, and that EIF4G5 is as abundant as EIF4G4 [181]. EIF4E6 might, therefore, have a constitutive function. RNAi targeting *EIF4E6* in procyclics resulted in flagellar detachment [176]. (See also electronic supplementary material, Note 31.)

Both EIF4E1 and EIF4E2 are, unlike the other EIF4Es, found in the nucleus as well as the cytoplasm and have lower abundances than EIF4E3 and EIF4E4 [174]. Neither co-purifies with an EIF4G. EIF4E2 pairs with a protein that has a double-stranded RNA-binding domain [172]. The functions of EIF4E2 and its partner are not known.

EIF4E1 is the only *T. brucei* EIF4E that suppresses expression when tethered. It interacts with a protein called 4EIP in both *Leishmania* [168] and *T. bruce*i [171]. 4EIP binds directly to mRNA [41] and, when tethered, is a suppressor even when EIF4E1 is absent [171]. By contrast, the suppressive activity of EIF4E1 depends on its interaction with 4EIP. The phenotypes of trypanosome depletion mutants suggest that the two proteins may act both together and independently. 4EIP is required to suppress translation in stumpy forms, but EIF4E1 is not; by contrast, procyclic forms require EIF4E1 but not 4EIP [171]. The results suggest that EIF4E1 is either unable to initiate translation independently, or is able to do so only weakly. Binding of 4EIP to EIF4E1 stabilizes EIF4E1, but simultaneously reduces the EIF4E1–cap interaction [182] (electronic supplementary material, Note 32).

In mammalian cells, the first or ‘pioneer’ round of translation involves eIF4AIII [183] and is initiated by a specialized eIF4G-like protein that interacts with CBP80 [184]. Nothing is known about the pioneer round of translation in kinetoplastids but there is no indication that the nuclear cap-binding complex has any translation-stimulating properties.

### 4.2. Scanning, elongation and termination

Before initiation, the 48S complex has to scan through the 5′-UTR in order to reach the initiation codon. Scanning can be impeded by secondary structures, which are unwound by eIF4A and additional RNA helicases [185–188]. Most kinetoplastid 5′-UTRs are under 100 nt long, and they rarely exceed 250 nt [29,103,189]. In *T. brucei*, both hairpins and 5′-UTRs longer than 200 nt were shown to decrease expression of a reporter protein [190], suggesting that 48S complexes may be lost during scanning. *T. brucei* has 13 predicted DEAD/H-box helicases, of which eight are known to be in the cytosol (electronic supplementary material, table S1). With the exception of EIF4A1 and DHH1, the functions of these proteins cannot readily be assigned by sequence comparisons. Two of them, however, are likely to be involved in translation since they can complement a yeast Ded1p mutant [191].

As noted above in relation to figure 3*e*, if there is a short open reading frame upstream of the principal coding region (uORF), translation may initiate and terminate before the principal initiation codon is reached. As a consequence, ribosome densities on opisthokont mRNAs with uORFs are lower than on mRNAs that lack them [192]. Roughly a tenth of *T. brucei* 5′-UTRs contain uORFs with ribosome occupancy [193,194]. The relevant mRNAs have significantly lower ribosome densities on the main ORF than mRNAs without uORFs [193,194] (electronic supplementary material, Note 33). They also have lower mRNA abundances [193,194], although they are only slightly more unstable [38] (electronic supplementary material, Note 34).

In prokaryotes and eukaryotes, rates of translation elongation are strongly influenced by codon composition, and slow translation correlates with lower mRNA abundance [195]. Variations in tRNA abundance are thought to underlie the influence of codon composition, with strings of codons with low tRNA abundances causing upstream ribosome traffic jams [195]. The cellular abundances of tRNAs are not known for kinetoplastids. Nevertheless, two studies have shown that codon composition influences translation and mRNA abundance: sub-optimal codons tend to result in less RNA and less protein [196,197].

In opisthokonts and plants, a quality control pathway called ‘nonsense-mediated decay’ ensures removal of mutant mRNAs in which a termination codon is present within the normal open reading frame [198,199]. This requires, minimally, termination factors eRF1 and eRF3 and the interacting nonsense-mediated decay factors Upf1 and Upf2 [199]. Kinetoplastids have homologues of all four proteins, and UPF1 and UPF2 mutually interact. However, RNAi targeting had no reproducible effect either [190,200] and it is not clear whether a classical nonsense-mediated decay pathway is present or not [190] (electronic supplementary material, Note 35).

## 5. mRNA decay

### 5.1. Removal of the poly(A) tail and 3′→5′ degradation

Opisthokont mRNA decay usually commences with deadenylation by the NOT complex (figure 2(11)). The NOT complex has been found in all eukaryotes examined so far. It has at least six subunits, and assembles on a large scaffold protein, NOT1; its activity is inhibited by PABP [201–203] (electronic supplementary material, Note 36). NOT complexes from many species are associated with two 3′→5′ exoribonucleases, but trypanosomes have only one— CAF1 [204,205]. Transcriptome-wide measurements of mRNA half-lives after inhibition of RNA synthesis yielded median half-lives of 12 min in bloodstream forms and 20 min in procyclic forms [38] (electronic supplementary material, Note 37). For the majority of mRNAs, deadenylation by the NOT complex was clearly the most important determinant of half-life [206]. RNAi targeting *CAF1* inhibited overall deadenylation, resulting in a marked increase in the half-lives of most mRNAs [204,207].

A second eukaryotic deadenylation complex consists of the enzyme PAN2 bound to a PAN3 dimer [203,208,209]. Mammalian PAN2/PAN3 can digest PABP-bound poly(A) and is important in trimming relatively long poly(A) tails [210]. In *T. brucei*, RNAi targeting PAN2 stabilizes mRNAs with intermediate half-lives [206] but has no effect on the steady-state poly(A) tail distribution and causes only a marginal delay in overall deadenylation [146].

Animal ‘poly(A) ribonuclease’ (PARN) enzymes have activities at specific developmental stages. Trypanosome genomes encode three PARNs that appear not to be implicated in constitutive deadenylation [211]. Their roles during differentiation or stress have not been studied.

Many questions remain concerning deadenylation in trypanosomatids. We do not know how tail lengths— which vary up to 200 nt [146,204]—are determined, or how deadenylases are differentially targeted to individual mRNAs. Counterintuitively, stable opisthokont mRNAs with high ribosome densities and optimal codon compositions have very short poly(A) tails [212]; the relationship is not known in trypanosomes.

After RNAi targeting the exosome or deadenylation, the effects on steady-state levels of many mRNAs correlated very poorly with the effects on decay rates [206]. Instead, after loss of CAF1, PAN2 or RRP45, changes in mRNA abundance correlated negatively with mRNA length [213]. Since the same was seen after loss of the transcription elongation factor CTR9 [213], is seems possible that loss of 3′→5′ decay causes feedback inhibition of transcription, but this has not been verified.

### 5.2. Decapping and degradation of the body of the mRNA

The shortest poly(A) tail length that is seen, in both opisthokonts and trypanosomes, is about 30 nt [204], which is slightly above the 25 nt that is occupied by a single PABP [152]. Since binding of opisthokont PABPs to poly(A) tails is enhanced by cooperative interactions [152], it is thought that this final PABP monomer can be removed relatively easily, after which degradation of the body of the mRNA is triggered. Although loss of PABP will expose the mRNA to the exosome, the major pathway is removal of the mRNA cap by decapping enzymes (figure 2(12)), followed by 5′→3′ degradation (figure 2(13)). This can start while the mRNA is still being translated [214,215]. In trypanosomes too, deadenylation followed by decapping and 5′→3′ degradation seems to be the predominant decay mechanism.

mRNA decapping enzymes are usually members of the MutT hydrolase family. The products are 5′-monophosphorylated mRNAs, which are substrates for the 5′→3′ exoribonuclease Xrn1 [83]. Kinetoplastids have five potential MutT hydrolases but none is implicated in decapping of deadenylated mRNAs. Instead, caps are removed by ALPH1, an ApaH-like phosphatase which removes the cap to leave an mRNA with a 5′-diphosphate [216]. An additional 5′-phosphatase may be required before the trypanosome Xrn1 homologue, XRNA [217], can digest the mRNA. We do not know how ALPH1 is recruited to its mRNA targets.

In addition to ALPH1, *T. brucei* has several enzymes that seem likely to be involved in cap repair or quality control. These include a MutT hydrolase that preferentially acts on unmodified caps [218] (electronic supplementary material, Note 38) and a protein with a guanylyltransferase domain that co-purified with mRNA, EIF4E5 and EIF4G1 [41,175]. Finally, an enzyme with activity similar to that of yeast DcpS was detected [219] but there is no obvious homologue in the *T. brucei* genome.

After decapping, XRNA is probably important for the degradation of most mRNAs [217]; this was illustrated at the single-molecule level for one mRNA [220]. The exosome plays a relatively minor role in mRNA degradation [206], and the roles of two additional 5′→3′ exoribonucleases [60] are unknown. Depletion of XRNA was lethal, but preferentially stabilized mRNAs with very rapid decay, suggesting that for these mRNAs, 5′→3′ degradation (rather than deadenylation) was limiting [217] (figure 2 (14)). Detailed examination of the mRNA encoding EP procyclin in bloodstream forms revealed that, rather unusually, it is degraded from both ends, with contributions from XRNA, CAF1, PAN2 and the exosome [60,146,221,222] (electronic supplementary material, Note 39). The combined results suggest that this and other similarly unstable mRNAs are attacked by the 5′→3′ degradation machinery without prior deadenylation. Nevertheless, this must start after export is complete, since the degradation rates are determined by sequences in the mRNA 3′-UTRs (figure 2 (14)).

In yeast, the RNA helicase Dhh1 is associated with mRNAs with slow elongation, and activates decapping [223]. Trypanosome DHH1 probably has several different roles in gene expression: it has been found in affinity-purified preparations of NOT complex components and various other proteins implicated in mRNA metabolism. Depletion of DHH1, or over-expression of a catalytically dead mutant, in procyclic forms led to accumulation of bloodstream-form-specific mRNAs [224].

### 5.3. The role of untranslated regions

Decay rates are a major determinant of trypanosomatid mRNA abundance. Codon usage contributes strongly to constitutive decay rates, giving at least a 25-fold range [196,197], but most sequences responsible for regulation in response to environmental change are in 3′-UTRs. Often, regions of at least 100 nt are found to be necessary for regulation. This may be because secondary structure is important, or because binding by several different proteins is required. A median *T. brucei* 3′-UTR of about 400 nt [29,103,189] would have space to bind at least 13 proteins (electronic supplementary material, Note 40).

### 5.4. Environmental adaptation and differentiation

Most studies of the mechanism of gene expression regulation have focused on differences between different life cycle stages or responses to individual developmental triggers (figure 1). Interpretation of results with growing cells is relatively straightforward. By contrast, with growth-arrested stages, it is difficult to distinguish genuine developmental regulation from the effects of stress or arrest *per se*. For example, during formation of stumpy *T. brucei*, there is downregulation of both translation and transcription [225,226], and translation arrest by itself can block mRNA degradation [196]. With the exception of naturally obtained life cycle stages, therefore, all results obtained with growth-arrested cells have to be treated with considerable caution (electronic supplementary material, Note 41).

Researchers have also looked for responses to individual nutrients. In *T. brucei*, changes in availability of the main substrates of energy metabolism in bloodstream and procyclic forms (glucose and proline respectively), and of glycerol in both forms, result in remodelling of metabolism [126,227–234]. Stablization of the *GPEET* procyclin mRNA via its 3′-UTR [227] is likely to be an indirect effect of changes in energy metabolism [228]. The increased expression of transferrin receptor mRNAs in response to iron deprivation [235], feedback inhibition of the synthesis of the prozyme subunit of S-adenosylmethionine decarboxylase [236] and repression of purine transporter NT8 expression by purines [237] are also 3′-UTR mediated (electronic supplementary material, Note 42).

### 5.5. Regulation by RNA-binding proteins

All results so far indicate that control by UTRs is effected exclusively via RNA-binding proteins [238]. Although RNAi is active in *T. brucei* and some *Leishmania* species, many kinetoplastids completely lack the RNAi machinery and its role is probably restricted to suppression of retroposons and viruses [239]. There is no experimental evidence for miRNAs [240] (electronic supplementary material, Note 4).

The behaviour and fate of each mRNA will depend on dynamic, combinatorial interactions between that mRNA and the various proteins that compete for binding (e.g. [241]). An annotated list of *T. brucei* proteins with RNA-binding domains is included in electronic supplementary material, table 1, sheet 1. The domains include approximately 90-residue RRMs, CCCH zinc finger domains (consensus: CX_8_CX_5_CX_3_H) and pumilio (Puf) domains. Most RNA-binding proteins are likely to be present in considerable excess over their target mRNAs [178], ensuring that, with low nM dissociation constants [242–244], the majority of potential binding sites in mRNAs will be occupied. The combination of mRNA and proteins is often called a ‘messenger ribonucleoprotein particle’ (mRNP) (electronic supplementary material, Note 43). A catalogue of all proteins bound directly to mRNA in bloodstream forms (mRNP proteome) is already available [41], and the ability of each protein to activate, or repress, expression when tethered to the 3′-UTR of a reporter RNA has also been assessed [41,167] (electronic supplementary material, Notes 30 and 44). In the following section, I will describe the roles of selected RNA-binding proteins in particular aspects of mRNA control.

### 5.6. Cell-cycle regulation

As in other eukaryotes, the abundances of some kinetoplastid mRNAs and proteins vary during the cell cycle [245]. This has been examined in detail only in *Crithidia fasciculata*, where levels of a few cell-cycle-regulated mRNAs are controlled by two complexes called CSBPI and CSBPII [246]. CSBPI consists of two proteins with CCCH zinc finger domains, CSBPA and CSBPB. The trypanosome homologues, ZC3H39 and ZC3H40, mutually interact, bind to mRNA and repress when tethered [41,247] (electronic supplementary material, Note 45).

The *Crithidia* CSBPII complex contains poly(A) binding protein and two other proteins whose activities are controlled by phosphorylation. CSBPII_45 binds specifically to a 5′-UTR motif that is required for cell-cycle regulation (electronic supplementary material, table S1) [248]. Both *T. brucei* CSBPII homologues cross-link to *T. brucei* mRNA and CSBPII_33 is a repressor when tethered [41,167], but the *Crithidia* regulatory motif is not found in trypanosome cell-cycle-regulated mRNAs [245].

In *T. brucei*, the pumilio domain protein PUF9 binds to, and stabilizes, some mRNAs that are preferentially increased during S-phase [249]. A motif that includes the Puf core recognition motif, UGUA, was required for binding and regulation [249]. Stabilization by PUF9 may depend on the MKT complex, which is described later. PUF9 is unusual, because Puf-domain proteins usually repress translation and promote mRNA decay [250]. *T. brucei* PUFs 1, 2, 3 and 10 indeed suppress expression when tethered (electronic supplementary material, table S1). Results for *Leishmania* [251] and *T. cruzi* PUF6 indicate that it too is a suppressor; *Leishmania* PUF6 contributes to destruction of stage-regulated mRNAs containing a retroposon sequence [251].

### 5.7. Procyclic-form-specific mRNAs

**Figure 5.**
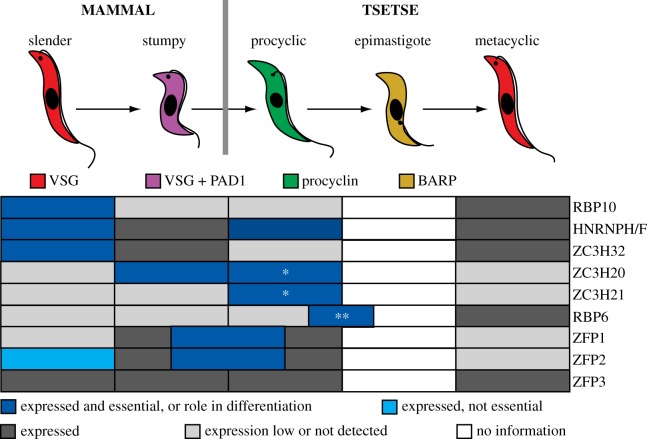
RNA-binding proteins that have been implicated in developmental regulation. The figure shows a simplified pathway from the bloodstream form to the metacyclic form. The proteins shown were chosen because they are well characterized. Several more proteins have been shown to be essential for differentiation; these, as well as other RNA-binding proteins that are expressed in a stage-specific manner, but whose roles in differentiation have yet to be defined in detail, are mentioned in the text or can be found in electronic supplementary material, table S1. Regulated expression is derived from proteome data for differentiation of bloodstream forms to procyclic forms [181,253,254] and for RBP6-induced differentiation of procyclic forms to metacyclic forms [255]. Proteome data for epimastigotes are not yet available. *RNAi targeting both simultaneously is lethal. **RBP6 expression triggers differentiation to epimastigotes, and the mRNA is present in proventricular and salivary gland parasites, but it is not known whether RBP6 is essential for differentiation. References for the phenotypic results are in the text. ‘Essential’ usually means that RNAi was detrimental; the absence of an RNAi effect does not necessarily mean that the protein is not required.

Several hundred mRNAs are much more abundant and/or better translated in procyclic forms than in bloodstream forms. Some of these, including those encoding EP procyclin, cytosolic phosphoglycerate kinase (PGKB) and various cytochrome subunits, have one or more U(A)U_6_ motifs in their 3′-UTR. This motif is bound by a repressive, bloodstream-form-specific protein called RBP10 [252] (figure 5). Bloodstream forms with depleted RBP10 show increases in many of the target mRNAs [252,256], and can grow only if transferred to procyclic conditions [252]. Conversely, procyclic forms with forced expression of RBP10 can grow only as bloodstream forms [252]. The mechanism by which RBP10 represses translation and promotes mRNA decay is not known: searches for interaction partners did not reliably reveal interactions with the degradation machinery [252,257] (electronic supplementary material, Note 46). RBP9 [257,258] and ZC3H32 [259] are additional bloodstream-form-specific repressors (figure 5) but so far their roles are unclear (electronic supplementary material, Note 47). In yeast, poor translation initiation correlates to some extent with rapid mRNA decay [260]. Similarly, tethering of RBP10 [252], ZC3H32 [259] or 4EIP [171] causes both translation inhibition and mRNA destruction, but in each case, it is difficult to determine what happens first.

The converse side of regulation for procyclic-form-specific mRNAs is stabilization and/or translation enhancement in the procyclic form. To try to identify the proteins resonsible, affinity purification using the U(A)U_6_-containing regulatory element from *PGKB* was undertaken. This yielded UBP2, RBP33, RBP42 and DRBD3 [261]. These are constitutively expressed abundant RNA-binding proteins with a preference for U-rich sequences. RBP33 was discussed earlier; the remaining proteins will be discussed later.

ZFPs 1, 2 and 3 (figure 5) mutually interact [262]. ZFP1 expression is upregulated during differentiation and remains high in procyclic forms [263], while ZFP2 [263] and ZFP3 [262] are constitutvely expressed. All three are implicated in differentiation. ZFP3 associates with the EP procyclin 3′-UTR [262,264] and might be implicated in the switch from early to late procyclic forms [264], but it also associates with many constitutively expressed mRNAs [265] (electronic supplementary material, Note 48). Expression of the membrane protein ESAG9 is suppressed in long slender bloodstream forms and procyclic forms, and elevated in stumpy forms. A screen for proteins required for the suppression in bloodstream forms revealed ZFP3, DRBD5 and a repressive RNA-binding protein, REG9.1 [266]. REG9.1 is most abundant in procyclic forms, where it is essential [266]. Its mode of action and direct targets are unknown.

RNAi targeting HNRNPH resulted in a loss of developmental regulation, and its putative binding motif was enriched in the UTRs of affected mRNAs. However, the effects of depletion were often opposite in bloodstream and procyclic forms. For example, the effect of HNRNPH in *stabilizing* an amino acid transporter (*AATP11*) mRNA in procyclic forms depended on the presence of the binding motif in the 3′-UTR [144]; but the same mRNA was *increased* after HNRNPF/H depletion in bloodstream forms, suggesting that HNRNPH was acting as a *repressor* [144]. It is possible that the activity of HNRNPH is changed by post-translational modification or protein–protein interactions; alternatively, some effects might be secondary. Tethering results for full-length HNRNPH, which might help to resolve this, are not available.

ZC3H20, ZC3H21 and ZC3H22 share a region containing two CCCH zinc finger domains; their mRNAs are targeted by RBP10. All three proteins increase in procyclic forms but ZC3H20 appears earlier in differentiation [181]. RNAi targeting both ZC3H20 and ZC3H21 inhibits procyclic form growth [267] and ZC3H20 binds to, and stabilizes, mRNAs encoding some procyclic-specific membrane proteins. The role of ZC3H22 is unknown.

After differentiation from the bloodstream form, *T. brucei* initially grows as ‘early’ procyclic forms which express GPEET and EP procyclins. Later, GPEET mRNA is destabilized, and non-templated oligo-U tails were detected [268]. In opisthokonts, addition of oligo-U tails to mRNAs is a trigger for their 3′→5′ degradation by the enzyme Dis3L2 [269], and the U tails on GPEET are the first indication that this pathway exists in trypanosomes. Trypanosomes have a Dis3L2 homologue but its function is unknown and the terminal uridylyl transferase responsible for cytosolic U addition has not been identified (electronic supplementary material, Note 49).

### 5.8. Bloodstream-form-specific mRNAs

So far, no common sequence motifs or binding proteins have been found for mRNAs that are specific for the bloodstream form. A 16mer sequence in the *VSG* 3′-UTR is implicated in its stability in bloodstream forms [66]; in procyclic forms, the 3′-UTR gives a relatively normal half-life of about 25 min [270]. Selection for mutations that suppressed the function of the *GPIPLC* 3′-UTR in procyclic forms resulted in 3′ deletions, but the smallest regulatory 3′-UTR was 800 nt [271]. Results for the *PGKC* 3′-UTR implicated the 3′-most 360 nt in the suppression of expression in procyclic forms [272]. Oddly, this segment contains two copies of the RBP10 binding motif, despite the very high stability of *PGKC* mRNA in bloodstream forms.

The *TCTP* genes provide an example of competition between regulatory sequences. The *TCTP1* mRNA includes an RBP10 binding motif, and has very low expression in bloodstream forms, whereas the *TCTP2* mRNA shows opposite regulation. The first 160 nt of the *TCTP2* 3′-UTR are needed for optimal bloodstream-form expression; and when they are inserted upstream of the *TCTP1* 3′-UTR, developmental regulation of the latter is lost, suggesting that these 160 nt can prevent the effects of the RBP10 binding site [273].

### 5.9. From procyclic to metacyclic forms

RBP6 has a single RRM domain and its expression is highest in salivary gland *T. brucei* [274,275]. Inducible expression of RBP6 in procyclic forms triggered their differentiation to epimastigotes then infectious metacyclic trypomastigotes [276]. Expression of a point mutant of RBP6 resulted in a ‘jump’ to infectious metacyclic forms without the appearance of epimastigotes [277]—which is also what happens after expression of RBP10 in procyclic forms. The direct RNA targets of RBP6 *in vivo* are not known, although it is tempting to speculate that it may stimulate RBP10 expression (electronic supplementary material, Note 50). Transcriptome and proteome results of the different stages [255,274,275,278] mainly reflect the transition from the procyclic to the bloodstream form, and growth arrest in metacyclic forms, but parasites with induced RBP6 also showed increases in mRNAs encoding RNA-binding proteins that might be specific to epimastigotes or metacyclic forms (electronic supplementary material, Note 51). One, RBP7, was also found to be needed for stumpy formation [279], so it might be implicated in developmental growth arrest.

DRBD13 is a repressor with several hundred identified bound mRNAs [280,281]. Both RNAi and over-expression killed procyclic forms, indicating that DRBD13 protein dosage is important [281]. The data were partially consistent with DRBD13 being a suppressor of RBP6 expression, but effects on the transcriptome after *DRBD13* RNAi were relatively minor [280,281] (electronic supplementary material, Note 52).

### 5.10. Abundant mRNA-binding proteins

Quantitative mass spectrometry results suggest that some RNA-binding proteins have abundances similar to those of the PABPs [181]. The ALBA proteins are top of the list, and were estimated by quantitative immunoblotting to be present at 10–20 000 molecules per procyclic trypanosome [282]. From the mass spectrometry results, UBP1 and UBP2, RBP42 and DRBD3/PTB1 are only slightly less abundant. To place this in context, a procyclic cell has about 44 000 mRNAs [45], and in *Leishmania* promastigotes the three PABPs are each present at around 1–2 × 10^5^ molecules/cell [153]. V5-tagged UBP2 was estimated at over a million molecules per cell [283], but this seems anomalous—perhaps the protein was stabilized by the tag. Abundant RNA-binding proteins may be general RNA chaperones, although they could also have sequence-specific functions. The four cytoplasmic ALBA domain proteins mutually interact, and tandem affinity purification yielded PABP2, EIF4E4 and EIF4G3 [282], consistent with their being associated with translating mRNA. Pull-down of both PABPs reciprocally confirmed association with ALBA proteins [155].

The small RNA-binding proteins UBP1 and UBP2 prefer U-rich sequences [284], can homo- and hetero-dimerize [285] and show RNA-binding-dependent shuttling from the nucleus to the cytosol [286]. Only a few possible specific target mRNAs were detected [287–289] (electronic supplementary material, Note 53).

RBP42 and DRBD3/PTB1 are the only mRNA-binding proteins for which *in vivo* binding sites have been mapped in detail by identification of cross-linked nucleotides. RBP42 is essential in all tested life cycle stages, is found in the mRNP proteome, and stimulates when tethered. Binding sites in procyclic forms were particularly common in the coding regions of mRNAs of energy metabolism [290]. No consensus binding motif was reported. DRBD3 is also an activator of expression, and consistent with this, the 265 bound mRNAs are relatively stable. DRBD3 binds predominantly in 3′-UTRs [143,290]. Microarrays detected changes in only eight of the 265 bound mRNAs after DRBD3 depletion [142,261], but the results did suggest that DRBD3 is important to maintain the stability of some bound mRNAs in procyclic forms [261] (electronic supplementary material, Note 54).

From mass spectrometry results, additional constitutively expressed abundant RNA-binding proteins are ZC3H41, DRBD2 and DRBD18, all of which are mRNA bound (electronic supplementary material, table S1). DRBD18 is described later.

### 5.11. Stress responses and ZC3H11

When eukaryotic cells are starved, over-heated or subjected to some chemical stresses, mRNAs and their associated proteins aggregate to form stress granules, which are thought to protect the mRNAs and enable renewed translation once normal conditions are restored [291,292] (figure 2 (9); electronic supplementary material, Note 55). Some effects of translation inhibition on mRNA decay may be caused by stress responses (electronic supplementary material, Note 56).

Most work on heat shock has been done with procyclic-form trypanosomes, which have a standard growth temperature of 27°C. After a severe heat shock (41°C), trypanosomes shut down transcription initiation [293]. In *T. cruzi*, this is accompanied (or perhaps caused) by RNA polymerase II large subunit dephosphorylation [294]. Translation of most *T. brucei* mRNAs is also suppressed, by an unknown mechanism, with either transfer to stress granules or degradation [295]. Effects are similar, but less extreme, at 39°C or the more physiological temperature of 37°C [296]. However, a subset of mRNAs, including those encoding all components of the protein refolding machinery, shows less granule association, and continues translation [296]. Initially, continued synthesis of these mRNAs is ensured by the fact that the genes are towards the ends of transcription units, so mRNAs can be made by elongating polymerases [297] (electronic supplementary material, Note 57). Meanwhile, maintenance of mRNA stability and translation is ensured by sequences in their 3′-UTRs.

The *HSP70* 3′-UTR is necessary and sufficient for the continued presence [298], stability and translation [295] of the mRNA after heat shock (electronic supplementary material, Note 57), and this requires a (UAU)n repeat which is bound by ZC3H11 and also found in other heat-shock protein transcripts. RNAi targeting *ZC3H11* caused death of bloodstream forms, while procyclic forms became highly susceptible to heat shock [299]. In heat-shocked procyclic forms, mRNAs that bind to ZC3H11 are preferentially retained on polysomes and excluded from heat-shock granules [296]. In both forms, ZC3H11 recruits an activating complex consisting of MKT1, PBP1, LSM12 and XAC1 [300]; PBP1 can recruit both PABPs. MKT1 complexes are probably implicated in the stabilization of many mRNAs, since MKT1 shows a two-hybrid interaction and/or *in vivo* association with 21 additional RNA-binding proteins, all but three of which activated when tethered (electronic supplementary material, table S1, sheet 1). Twelve of the interactors contain the motif H(E/D/N/Q)PY, which is necessary and sufficient for the interaction with MKT1, and several of the others have polyglutamine repeats (electronic supplementary material, table S1, sheet 1). MKT1 is essential in bloodstream forms; roles in procyclic forms are unproven (electronic supplementary material, Note 58).

Trypanosome starvation granules and heat-shock granules contain much the same set of proteins as opisthokont stress granules [295,301–303]. Interestingly, only PABP2, but not PABP1, accumulates in stress granules [154]. SCD6, an evolutionarily conserved granule component, functions as a translation repressor in trypanosomes [304]; upon over-expression, it aggregates and promotes granule formation [305] (electronic supplementary material, Note 59). Curiously, starvation causes ALPH1 and XRNA to move to a specialized stress granule at the posterior end of the cell [303]. The significance of this is unknown.

### 5.12. The odd behaviour of mRNAs encoding ribosomal proteins

**Figure 6.**
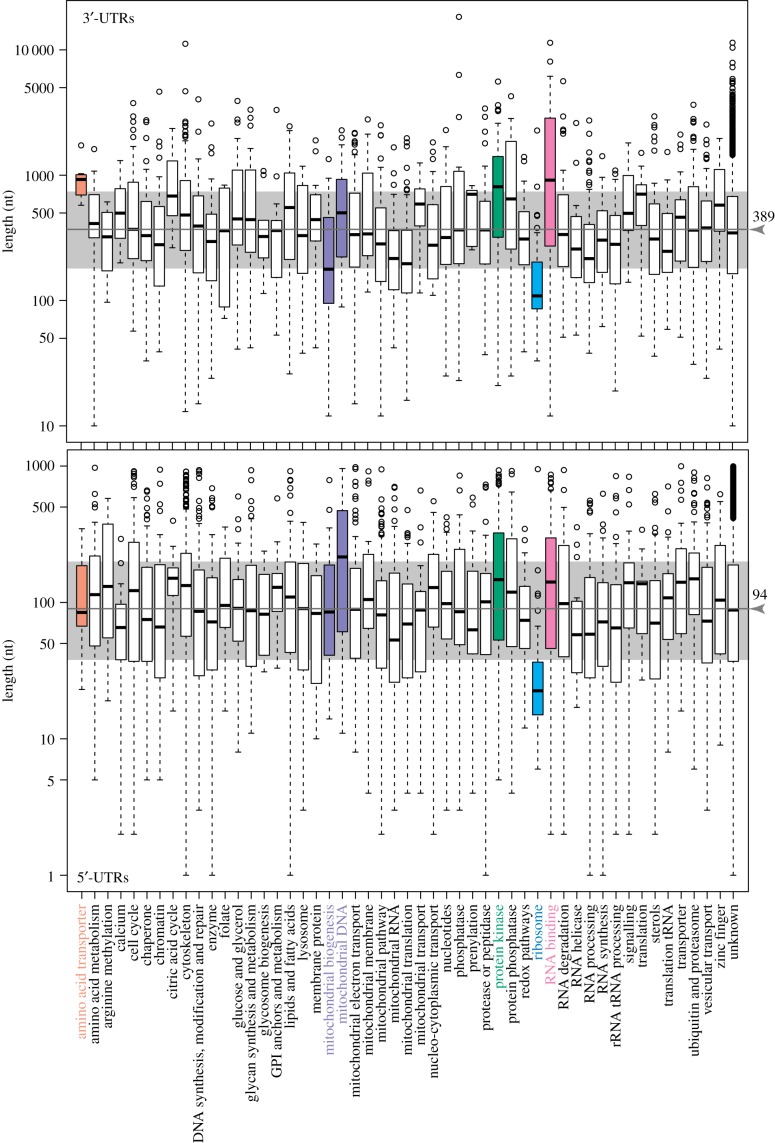
Lengths of 3′- and 5′-UTRs for mRNAs encoding proteins belonging to different functional classes. Protein classes were assigned manually, according to genome annotations and publications [171]. UTR lengths were downloaded from TritrypDB and are shown on a log scale (electronic supplementary material, Note 40). The boxes indicate the 25th and 75th percentiles, and the bar within the box is the median (label is in nt). Whiskers extend to the most extreme measurement that is within 1.5× the inter-quartile range, and spots are outliers. The dark grey line indicates the overall median, and the pale grey area is between the overall 25th and 75th quartiles. Colours indicate classes in which the medians for the 5′- and/or 3′-UTRs are outside the overall 25th–75th percentile range.

When cells are stressed, ribosomal protein synthesis is not required. It is, therefore, curious that the mRNAs encoding *T. brucei* ribosomal proteins are excluded from both starvation and heat-shock granules [296,303]. Ribosomal protein mRNAs are very stable but have abnormally low ribosome occupancy (electronic supplementary material, Note 60). This might be because they have very good codon optimality scores [196], which are expected to result in rapid elongation [306]. These mRNAs are also notable for their short 5′- and 3′-UTRs (figure 6). So far, the only RNA-binding protein known to be associated with these mRNAs is DRBD3 [143] (electronic supplementary material, Note 61).

### 5.13. Regulating the regulators

As RNA-binding proteins are vital for gene expression control, it is essential that they too are tightly regulated. More than 30 *T. brucei* RNA-binding proteins show at least twofold differences in synthesis between bloodstream and procyclic forms [126,193], with contributions from both mRNA level and translation efficiency. Even more are regulated at the protein level [181], where proteolysis can play a role. In addition, more than half are phosphorylated [24], and 11 have methylated arginine residues [307] (electronic supplementary material, table S1). At least one is SUMOylated (as are some exosome subunits) [308], and several others are acetylated [309] (electronic supplementary material, table S1).

Intriguingly, the mRNAs encoding many RNA-binding proteins have unusually long 3′-UTRs—often several kb long—and this is also true for protein kinases (figure 6). There could be two reasons for this. One is the inverse correlation between abundance and mRNA length: the long 3′-UTR may help to ensure low mRNA abundance. The other possibility is that because the long 3′-UTRs can bind many regulatory proteins, control can be more stringent or flexible than for shorter 3′-UTRs. The *ZC3H20*, *ZC3H21* and *ZC3H22* mRNAs are a clear example, with 3′-UTRs longer than 4 kb. *ZC3H20* mRNA has two RBP10 binding sites, and ZC3H20 becomes detectable during stumpy formation [181]; but the other two mRNAs have five and nine binding sites, respectively [252], and the proteins are detected only later in differentiation, when RBP10 has disappeared [181]. The presence of so many binding sites for the same RNA-binding protein is suggestive of a fail-safe mechanism which ensures that the mRNA is destroyed even if polyadenylation occurs upstream of the dominant sites.

ZC3H11 expression increases rapidly upon heat shock—but this has to happen with very little mRNA synthesis. Instead, translation of existing *ZC3H11* mRNA is strongly upregulated, governed by a 70 nt region in the 3′-UTR. ZC3H11 protein stability also increases, probably as a consequence of dephosphorylation [310].

DRBD18 is a substrate of the arginine methyltransferase PRMT1 [311] (electronic supplementary material, Note 62). Depletion of DRBD18 in procyclic forms resulted mainly in mRNA increases which were suggestive of at least partial procyclic–epimastogote–metacyclic differentiation. Whether this effect was direct or indirect is not clear. Further results indicated that methylation promotes mRNA stabilization by DRBD18, as well as changes its RNA-binding specificity and protein–protein interactions [311].

## 6. Future challenges

Numerous gaps in our knowledge have been mentioned in this review. We have no detailed information concerning regulation of mRNA processing, and do not know how the mature mRNA 5′-end is recognized for export from the nucleus. Very few kinetoplastid RNA-binding proteins have been quantitatively analysed using modern high-throughput methods; we do not know how they interact, or compete with each other, or, in most cases, how their activities are regulated by post-translational modification. Sequence-specific recruitment of the mRNA decay apparatus is a mystery: no interactions with destabilizing RNA-binding proteins have been found. We also do not know why there are five different translation-promoting cap-binding complexes. The biggest challenge, however, is disentangling the numerous interactions that can occur on a single mRNA.

Even in organisms that have transcription control many of the same questions remain to be answered, and it is quite possible that trypanosomatid solutions to these problems are conserved—or have evolved separately—in other supergroups.

## Supplementary Material

Table S1

Reviewer comments

## Supplementary Material

Supplementary Notes

## Data accessibility

This article has no additional data.

## References

[1] Adl SM et al. 2012 The revised classification of eukaryotes. J. Eukaryot. Microbiol. 59, 429–493. (10.1111/j.1550-7408.2012.00644.x) 23020233 PMC3483872

[2] Burki F 2014 The eukaryotic tree of life from a global phylogenomic perspective. Cold Spring Harb. Perspect. Biol. 6, a016147 (10.1101/cshperspect.a016147) 24789819 PMC3996474

[3] Begolo D et al. 2018 The trypanocidal benzoxaborole AN7973 inhibits trypanosome mRNA processing. PLoS Pathog. 14, e1007315 (10.1371/journal.ppat.1007315) 30252911 PMC6173450

[4] Wall RJ et al. 2018 Clinical and veterinary trypanocidal benzoxaboroles target CPSF3. Proc. Natl Acad. Sci. USA 115, 9616–9621. (10.1073/pnas.1807915115) 30185555 PMC6156652

[5] Lukes J, Skalicky T, Tyc J, Votypka J, Yurchenko V 2014 Evolution of parasitism in kinetoplastid flagellates. Mol. Biochem. Parasitol. 195, 115–122. (10.1016/j.molbiopara.2014.05.007) 24893339

[6] Horn D 2014 Antigenic variation in African trypanosomes. Mol. Biochem. Parasitol. 195, 123–129. (10.1016/j.molbiopara.2014.05.001) 24859277 PMC4155160

[7] Rojas F et al. 2018 Oligopeptide signaling through TbGPR89 drives trypanosome quorum sensing. Cell 176, 306–317.316. (10.1016/j.cell.2018.10.041) 30503212 PMC6333907

[8] Rico E, Rojas F, Mony BM, Szoor B, Macgregor P, Matthews KR 2013 Bloodstream form pre-adaptation to the tsetse fly in *Trypanosoma brucei*. Front. Cell Infect. Microbiol. 3, 78 (10.3389/fcimb.2013.00078) 24294594 PMC3827541

[9] Rotureau B, Van Den Abbeele J 2013 Through the dark continent: African trypanosome development in the tsetse fly. Front. Cell Infect. Microbiol. 3, 53 (10.3389/fcimb.2013.00053) 24066283 PMC3776139

[10] El-Sayed NM et al. 2005 Comparative genomics of trypanosomatid parasitic protozoa. Science 309, 404–409. (10.1126/science.1112181) 16020724

[11] Clayton C 2016 Gene expression in Kinetoplastids. Curr. Opinion MIcrobiol. 32, 46–51. (10.1016/j.mib.2016.04.018) 27177350

[12] Jackson AP, Quail MA, Berriman M 2008 Insights into the genome sequence of a free-living Kinetoplastid: *Bodo saltans* (Kinetoplastida: Euglenozoa). BMC Genomics 9, 594 (10.1186/1471-2164-9-594) 19068121 PMC2621209

[13] Michaeli S 2011 Trans-splicing in trypanosomes: machinery and its impact on the parasite transcriptome. Future Microbiol. 6, 459–474. (10.2217/fmb.11.20) 21526946

[14] Clayton C, Michaeli S 2011 3′ processing in protists. Wiley Interdiscip. Rev. RNA 2, 247–255. (10.1002/wrna.49) 21957009

[15] Müller L et al. 2018 Genome organization and DNA accessibility control antigenic variation in trypanosomes. Nature 563, 121–125. (10.1038/s41586-018-0619-8) 30333624 PMC6784898

[16] Das A, Banday M, Bellofatto V 2008 RNA polymerase transcription machinery in trypanosomes. Eukaryot. Cell 7, 429–434. (10.1128/EC.00297-07) 17951525 PMC2268515

[17] Srivastava A, Badjatia N, Lee JH, Hao B, Gunzl A 2018 An RNA polymerase II-associated TFIIF-like complex is indispensable for SL RNA gene transcription in *Trypanosoma brucei*. Nucleic Acids Res. 46, 1695–1709. (10.1093/nar/gkx1198) 29186511 PMC5829719

[18] Ruan JP, Arhin GK, Ullu E, Tschudi C 2004 Functional characterization of a *Trypanosoma brucei* TATA-binding protein-related factor points to a universal regulator of transcription in trypanosomes. Mol. Cell. Biol. 24, 9610–9618. (10.1128/mcb.24.21.9610-9618.2004) 15485927 PMC522245

[19] Schimanski B, Nguyen TN, Gunzl A 2005 Characterization of a multisubunit transcription factor complex essential for spliced-leader RNA gene transcription in *Trypanosoma brucei*. Mol. Cell. Biol. 25, 7303–7313. (10.1128/MCB.25.16.7303-7313.2005) 16055738 PMC1190248

[20] Lecordier L, Devaux S, Uzureau P, Dierick J, Walgraffe D, Poelvoorde P, Pays E, Vanhamme L 2007 Characterization of a TFIIH homologue from *Trypanosoma brucei*. Mol. Microbiol. 64, 1164–1181. (10.1111/j.1365-2958.2007.05725.x) 17542913

[21] Lee JH, Cai G, Panigrahi AK, Dunham-Ems S, Nguyen TN, Radolf JD, Asturias FJ, Gunzl A 2010 A TFIIH-associated mediator head is a basal factor of small nuclear spliced leader RNA gene transcription in early-diverged trypanosomes. Mol. Cell. Biol. 30, 5502–5513. (10.1128/mcb.00966-10) 20876299 PMC2976424

[22] Ouna B, Nyambega B, Manful T, Helbig C, Males M, Fadda A, Clayton C 2012 Depletion of trypanosome CTR9 leads to gene expression defects. PLoS ONE 7, e34256 (10.1371/journal.pone.0034256) 22532828 PMC3332058

[23] Gonzalez N, Sato M, Tomomori-Sato S, Conaway C, Conaway JW, Conaway RC 2018 CTD-dependent and -independent mechanisms govern co-transcriptional capping of Pol II transcripts. Nat. Commun. 9, 3392 (10.1038/s41467-018-05923-w) 30139934 PMC6107522

[24] Urbaniak MD, Martin D, Ferguson MA 2013 Global quantitative SILAC phosphoproteomics reveals differential phosphorylation is widespread between the procyclic and bloodstream form lifecycle stages of *Trypanosoma brucei*. J. Proteome Res. 12, 2233–2244. (10.1021/pr400086y) 23485197 PMC3646404

[25] Das A, Banday M, Fisher MA, Chang YJ, Rosenfeld J, Bellofatto V 2017 An essential domain of an early-diverged RNA polymerase II functions to accurately decode a primitive chromatin landscape. Nucleic Acids Res. 45, 7886–7896. (10.1093/nar/gkx486) 28575287 PMC5570084

[26] Das A, Bellofatto V 2009 The non-canonical CTD of RNAP-II is essential for productive RNA synthesis in *Trypanosoma brucei*. PLoS ONE 4, e6959 (10.1371/journal.pone.0006959) 19742309 PMC2734056

[27] Chapman AB, Agabian N 1994 *Trypanosoma brucei* RNA polymerase II is phosphorylated in the absence of carboxyl-terminal domain heptapeptide repeats. J. Biol. Chem. 269, 4754–4760. 8106443

[28] Lee JH, Jung HS, Gunzl A 2009 Transcriptionally active TFIIH of the early-diverged eukaryote *Trypanosoma brucei* harbors two novel core subunits but not a cyclin-activating kinase complex. Nucleic Acids Res. 37, 3811–3820. (10.1093/nar/gkp236) 19386623 PMC2699521

[29] Kolev N, Franklin J, Carmi S, Shi H, Michaeli S, Tschudi C 2010 The transcriptome of the human pathogen *Trypanosoma brucei* at single-nucleotide resolution. PLoS Pathog. 6, e1001090 (10.1371/journal.ppat.1001090) 20838601 PMC2936537

[30] Wedel C, Förstner K, Derr R, Siegel N 2017 GT-rich promoters can drive RNA pol II transcription and deposition of H2A.Z in African trypanosomes. EMBO J. 36, 2581–2594. (10.15252/embj.201695323) 28701485 PMC5579346

[31] Siegel T, Hekstra D, Kemp L, Figueiredo L, Lowell J, Fenyo D, Wang X, Dewell S, Cross G 2009 Four histone variants mark the boundaries of polycistronic transcription units in *Trypanosoma brucei*. Genes Dev. 23, 1063–1076. (10.1101/gad.1790409) 19369410 PMC2682952

[32] Wright JR, Siegel TN, Cross GA 2010 Histone H3 trimethylated at lysine 4 is enriched at probable transcription start sites in *Trypanosoma brucei*. Mol. Biochem. Parasitol. 172, 141–144. (10.1016/j.molbiopara.2010.03.013) 20347883 PMC2875994

[33] Briggs E, Hamilton G, Crouch K, Lapsley C, McCulloch R 2018 Genome-wide mapping reveals conserved and diverged R-loop activities in the unusual genetic landscape of the African trypanosome genome. Nucleic Acids Res. 46, 11 789–11 805. (10.1093/nar/gky928) 30304482 PMC6294496

[34] McAndrew M, Graham S, Hartmann C, Clayton CE 1998 Testing promoter activity in the trypanosome genome: isolation of a metacyclic-type VSG promoter, and unexpected insights into RNA polymerase II transcription. Exp. Parasitol. 90, 65–76. (10.1006/expr.1998.4317) 9709032

[35] Marchetti MA, Tschudi C, Silva E, Ullu E 1998 Physical and transcriptional analysis of the *Trypanosoma brucei* genome reveals a typical eukaryotic arrangement with close interspersion of RNA polymerase II- and III-transcribed genes. Nucleic Acids Res. 26, 3591–3598. (10.1093/nar/26.15.3591) 9671824 PMC147737

[36] LeBowitz JH, Coburn CM, McMahon-Pratt D, Beverley SM 1990 Development of a stable *Leishmania* expression vector and application to the study of parasite surface antigen genes. Proc. Natl Acad. Sci. USA 87, 9736–9740. (10.1073/pnas.87.24.9736) 2124701 PMC55248

[37] Martinez-Calvillo S, Nguyen D, Stuart K, Myler PJ 2004 Transcription initiation and termination on *Leishmania* major chromosome 3. Eukaryot. Cell 3, 506–517. (10.1128/EC.3.2.506-517.2004) 15075279 PMC387636

[38] Fadda A, Ryten M, Droll D, Rojas F, Färber V, Haanstra J, Bakker B, Matthews K, Clayton C 2014 Transcriptome-wide analysis of mRNA decay reveals complex degradation kinetics and suggests a role for co-transcriptional degradation in determining mRNA levels. Mol. Microbiol. 94, 307–326. (10.1111/mmi.12764) 25145465 PMC4285177

[39] Florini F, Naguleswaran A, Gharib WH, Bringaud F, Roditi I 2018 Unexpected diversity in eukaryotic transcription revealed by the retrotransposon hotspot family of *Trypanosoma brucei*. Nucleic Acids Res. 47, 1725–1739. (10.1093/nar/gky1255) PMC6393297 30544263

[40] Bringaud F et al. 2002 A new, expressed multigene family containing a hot spot for insertion of retroelements is associated with polymorphic subtelomeric regions of *Trypanosoma brucei*. Eukaryot. Cell 1, 137–151. (10.1128/EC.1.1.137-151.2002) 12455980 PMC118050

[41] Lueong S, Merce C, Fischer B, Hoheisel J, Erben E 2016 Gene expression regulatory networks in *Trypanosoma brucei*: insights into the role of the mRNA-binding proteome. Mol. Microbiol. 100, 457–471. (10.1111/mmi.13328) 26784394

[42] Das A, Zhang Q, Palenchar JB, Chatterjee B, Cross GA, Bellofatto V 2005 Trypanosomal TBP functions with the multisubunit transcription factor tSNAP to direct spliced-leader RNA gene expression. Mol. Cell. Biol. 25, 7314–7322. (10.1128/MCB.25.16.7314-7322.2005) 16055739 PMC1190245

[43] de Lange T, Liu A, Van der Ploeg L, Borst P, Tromp M, Van Boom J 1983 Tandem repetition of the 5′ mini-exon of variant surface glycoprotein genes: a multiple promoter for VSG gene transcription? Cell 34, 891–900. (10.1016/0092-8674(83)90546-9) 6313212

[44] Dossin FdM, Schenkman S 2005 Actively transcribing RNA polymerase II concentrates on spliced leader genes in the nucleus of *Trypanosoma cruzi*. Eukaryot. Cell 4, 960–970. (10.1128/EC.4.5.960-970.2005) 15879530 PMC1140094

[45] Haanstra J, Stewart M, Luu V-D, van Tuijl A, Westerhoff H, Clayton C, Bakker B 2008 Control and regulation of gene expression: quantitative analysis of the expression of phosphoglycerate kinase in bloodstream form *Trypanosoma brucei*. J. Biol. Chem. 283, 2495–2507. (10.1074/jbc.M705782200) 17991737

[46] Maree JP, Patterton HG 2014 The epigenome of *Trypanosoma brucei*: a regulatory interface to an unconventional transcriptional machine. Biochim. Biophys. Acta 1839, 743–750. (10.1016/j.bbagrm.2014.05.028) 24942804 PMC4138444

[47] Michaeli S 2012 Spliced leader RNA silencing (SLS)—a programmed cell death pathway in *Trypanosoma brucei* that is induced upon ER stress. Parasit Vectors 5, 107 (10.1186/1756-3305-5-107) 22650251 PMC3414811

[48] Hope R, Ben-Mayor E, Friedman N, Voloshin K, Biswas D, Matas D, Drori Y, Gunzl A, Michaeli S 2014 Phosphorylation of the TATA-binding protein activates the spliced leader silencing pathway in *Trypanosoma brucei*. Sci Signal. 7, ra85 (10.1126/scisignal.2005234) 25185157

[49] Loya T, Reinesa D 2016 Recent advances in understanding transcription termination by RNA polymerase II. F1000Res. 5, 1478 (10.12688/f1000research.8455.1) PMC4920211 27408690

[50] Reynolds D, Hofmeister BT, Cliffe L, Alabady M, Siegel TN, Schmitz RJ, Sabatini R 2016 Histone H3 variant regulates RNA polymerase II transcription termination and dual strand transcription of siRNA loci in *Trypanosoma brucei*. Elife 12, e1005758 (10.7554/eLife.11473.1371/journal.pgen.1005758) PMC4721609 26796527

[51] Borst P, Sabatini R 2008 Base J: discovery, biosynthesis, and possible functions. Annu. Rev. Microbiol. 62, 235–251. (10.1146/annurev.micro.62.081307.162750) 18729733

[52] Cliffe LJ, Siegel TN, Marshall M, Cross GA, Sabatini R 2010 Two thymidine hydroxylases differentially regulate the formation of glucosylated DNA at regions flanking polymerase II polycistronic transcription units throughout the genome of *Trypanosoma brucei*. Nucleic Acids Res. 38, 3923–3935. (10.1093/nar/gkq146) 20215442 PMC2896530

[53] Schulz D, Zaringhalam M, Papavasiliou FN, Kim HS 2016 Base J and H3.V regulate transcriptional termination in *Trypanosoma brucei*. PLoS Genet. 12, e1005762 (10.1371/journal.pgen.1005762) 26796638 PMC4721952

[54] Kawasaki F, Beraldi D, Hardisty RE, McInroy GR, van Delft P, Balasubramanian S 2017 Genome-wide mapping of 5-hydroxymethyluracil in the eukaryote parasite *Leishmania*. Genome Biol. 18, 23 (10.1186/s13059-017-1150-1) 28137275 PMC5282726

[55] van Luenen HG et al. 2012 Glucosylated hydroxymethyluracil, DNA base J, prevents transcriptional readthrough in *Leishmania*. Cell 150, 909–921. (10.1016/j.cell.2012.07.030) 22939620 PMC3684241

[56] Reynolds D, Cliffe L, Forstner KU, Hon CC, Siegel TN, Sabatini R 2014 Regulation of transcription termination by glucosylated hydroxymethyluracil, base J, in *Leishmania* *major* and *Trypanosoma brucei*. Nucleic Acids Res. 42, 9717–9729. (10.1093/nar/gku714) 25104019 PMC4150806

[57] Gommers-Ampt J, Lutgerink J, Borst P 1991 A novel DNA nucleotide in *Trypanosoma brucei* only present in the mammalian phase of the life-cycle. Nucleic Acids Res. 19, 1745–1751. (10.1093/nar/19.8.1745) 1674368 PMC328099

[58] Ekanayake DK, Minning T, Weatherly B, Gunasekera K, Nilsson D, Tarleton R, Ochsenreiter T, Sabatini R 2011 Epigenetic regulation of transcription and virulence in *Trypanosoma cruzi* by O-linked thymine glucosylation of DNA. Mol. Cell. Biol. 31, 1690–1700. (10.1128/mcb.01277-10) 21321080 PMC3126337

[59] Fernandez-Moya SM, Carrington M, Estevez AM 2014 Depletion of the RNA-binding protein RBP33 results in increased expression of silenced RNA polymerase II transcripts in *Trypanosoma brucei*. PLoS ONE 9, e107608 (10.1371/journal.pone.0107608) 25215501 PMC4162612

[60] Li C-H, Irmer H, Gudjonsdottir-Planck D, Freese S, Salm H, Haile S, Estévez AM, Clayton CE 2006 Roles of a *Trypanosoma brucei* 5′→3′ exoribonuclease homologue in mRNA degradation. RNA (New York, N.Y.) 12, 2171–2186. (10.1261/rna.291506) PMC1664730 17077271

[61] Sturm NR, Yu MC, Campbell DA 1999 Transcription termination and 3′-end processing of the spliced leader RNA in kinetoplastids. Mol. Cell. Biol. 19, 1595–1604. (10.1128/MCB.19.2.1595) 9891092 PMC116087

[62] Zeiner GM, Hitchcock RA, Sturm NR, Campbell DA 2004 3′-end polishing of the kinetoplastid spliced leader RNA is performed by SNIP, a 3′→5′ exonuclease with a motley assortment of small RNA substrates. Mol. Cell. Biol. 24, 10 390–10 396. (10.1128/MCB.24.23.10390-10396.2004) PMC529039 15542846

[63] Gunzl A, Bruderer T, Laufer G, Schimanski B, Tu LC, Chung HM, Lee PT, Lee MG 2003 RNA polymerase I transcribes procyclin genes and variant surface glycoprotein gene expression sites in *Trypanosoma brucei**.* Eukaryot. Cell 2, 542–551. (10.1128/EC.2.3.542-551.2003) 12796299 PMC161450

[64] Biebinger S, Rettenmaier S, Flaspohler J, Hartmann C, Peña-Diaz J, Wirtz LE, Hotz HR, Barry JD, Clayton CE 1996 The PARP promoter of *Trypanosoma brucei* is developmentally regulated in a chromosomal context. Nucleic Acids Res. 24, 1202–1211. (10.1093/nar/24.7.1202) 8614620 PMC145797

[65] Nguyen TN, Nguyen BN, Lee JH, Panigrahi AK, Gunzl A 2012 Characterization of a novel class I transcription factor A (CITFA) subunit that is indispensable for transcription by the multifunctional RNA polymerase I of *Trypanosoma brucei*. Eukaryot. Cell 11, 1573–1581. (10.1128/ec.00250-12) 23104567 PMC3536272

[66] Ridewood S, Ooi CP, Hall B, Trenaman A, Wand NV, Sioutas G, Scherwitzl I, Rudenko G 2017 The role of genomic location and flanking 3′UTR in the generation of functional levels of variant surface glycoprotein in *Trypanosoma brucei*. Mol. Microbiol. 106, 614–634. (10.1111/mmi.13838) 28906055 PMC5698767

[67] Ürményi TP, van der Ploeg LHT 1995 PARP promoter-mediated activation of a VSG expression site promoter in insect form *Trypanosoma brucei*. Nucleic. Acids Res. 23, 1010–1018. (10.1093/nar/23.6.1010) 7731788 PMC306799

[68] Hotz H-R, Lorenz P, Fischer R, Krieger S, Clayton CE 1995 Developmental regulation of hexose transporter mRNAs in *Trypanosoma brucei*. Mol. Biochem. Parasitol. 75, 1–14. (10.1016/0166-6851(95)02503-0) 8720170

[69] Huang J, van der Ploeg LHT 1991 Requirement of a poly-pyrimidine tract for trans-splicing in trypanosomes: discriminating the PARP promoter from the immediately adjacent 3′ splice acceptor site. EMBO J. 10, 3877–3885. (10.1002/j.1460-2075.1991.tb04957.x) 1935907 PMC453125

[70] Alsford S, Kawahara T, Glover L, Horn D 2005 Tagging a *T. brucei* RRNA locus improves stable transfection efficiency and circumvents inducible expression position effects. Mol. Biochem. Parasitol. 144, 142–148. (10.1016/j.molbiopara.2005.08.009) 16182389 PMC3833055

[71] Stanne T, Rudenko G 2010 Active VSG expression sites in *Trypanosoma brucei* are depleted of nucleosomes. Eukaryot. Cell 9, 136–147. (10.1128/EC.00281-09) 19915073 PMC2805301

[72] Navarro M, Gull K 2001 A pol I transcriptional body associated with VSG mono-allelic expression in *Trypanosoma brucei*. Nature 414, 759–763. (10.1038/414759a) 11742402

[73] Stanne T, Kushwaha M, Wand M, Taylor J, Rudenko G 2011 TbISWI regulates multiple Pol I transcribed loci and is present at Pol II transcription boundaries. Eukaryot. Cell 10, 964–976. (10.1128/EC.05048-11) 21571922 PMC3147422

[74] Rudenko G, Blundell PA, Dirks-Mulder A, Kieft R, Borst P 1995 A ribosomal DNA promoter replacing the promoter of a telomeric VSG expression site can be efficiently switched on and off in *T. brucei*. Cell 83, 547–553. (10.1016/0092-8674(95)90094-2) 7585957

[75] Glover L, Hutchinson S, Alsford S, Horn D 2016 VEX1 controls the allelic exclusion required for antigenic variation in trypanosomes. Proc. Natl Acad. Sci. USA 11, 7225–7230. (10.1073/pnas.1600344113) PMC4932947 27226299

[76] Schumann Burkard G, Kaser S, de Araujo PR, Schimanski B, Naguleswaran A, Knusel S, Heller M, Roditi I 2013 Nucleolar proteins regulate stage-specific gene expression and ribosomal RNA maturation in *Trypanosoma brucei*. Mol. Microbiol. 884, 827–840. (10.1111/mmi.12227) 23617823

[77] Perry KL, Watkins KP, Agabian N 1987 Trypanosome mRNAs have unusual “cap 4” structures acquired by addition of a spliced leader. Proc. Natl Acad. Sci. USA 84, 8190–8194. (10.1073/pnas.84.23.8190) 3120186 PMC299507

[78] Freistadt M, Cross G, Robertson H 1988 Discontinuously synthesized mRNA from *Trypanosoma brucei* contains the highly methylated 5′ cap structure, m7GpppA*A*C(2′-O)mU*A. J. Biol. Chem. 263, 15 071–15 075. 2844798

[79] Bangs JD, Crain PF, Hashizume T, McCloskey JA, Boothroyd JC 1992 Mass spectrometry of mRNA cap 4 from trypanosomatids reveals two novel nucleosides. J. Biol. Chem. 267, 9805–9815. 1349605

[80] Ruan J-P, Shen S, Ullu E, Tschudi C 2007 Evidence for a capping enzyme with specificity for the trypanosome spliced leader RNA. Mol. Biochem. Parasitol. 156, 246–254. (10.1016/j.molbiopara.2007.09.001) 17949828 PMC2706020

[81] Arhin G, Li H, Ullu E, Tschudi C 2006 A protein related to the vaccinia virus cap-specific methyltransferase VP39 is involved in cap 4 modification in *Trypanosoma brucei*. RNA (New York, N.Y.) 12, 53–62. (10.1261/rna.2223406) PMC1370885 16301606

[82] Zamudio J, Mittra B, Campbell D, Sturm N 2009 Hypermethylated cap 4 maximizes *Trypanosoma brucei* translation. Mol. Microbiol. 72, 1100–1110. (10.1111/j.1365-2958.2009.06696.x) 19504740 PMC2859698

[83] Kramer S, McLennan A 2018 The complex enzymology of mRNA decapping: enzymes of four classes cleave pyrophosphate bonds. WIRES RNA 10, e1511 (10.1002/wrna.1511) 30345629

[84] Ullu E, Tschudi C 1991 Trans splicing in trypanosomes requires methylation of the 5′ end of the spliced leader RNA. Proc. Natl Acad. Sci. USA 88, 10 074–10 078. (10.1073/pnas.88.22.10074) PMC52870 1719544

[85] Hury A, Goldshmidt H, Tkacz I, Michaeli S 2009 Trypanosome spliced-leader-associated RNA (SLA1) localization and implications for spliced-leader RNA biogenesis. Eukaryot. Cell 8, 56–68. (10.1128/EC.00322-08) 19028994 PMC2620742

[86] Mair G, Ullu E, Tschudi C 2000 Cotranscriptional cap 4 formation on the *Trypanosoma brucei* spliced leader RNA. J. Biol. Chem. 275, 28 994–28 999. (10.1074/jbc.M004193200) 10880518

[87] Badjatia N, Ambrosio DL, Lee JH, Gunzl A 2013 Trypanosome cdc2-related kinase 9 controls spliced leader RNA cap4 methylation and phosphorylation of RNA polymerase II subunit RPB1. Mol. Cell. Biol. 33, 1965–1975. (10.1128/mcb.00156-13) 23478263 PMC3647971

[88] Ambrosio DL, Badjatia N, Gunzl A 2015 The spliceosomal PRP19 complex of trypanosomes. Mol. Microbiol. 95, 885–901. (10.1111/mmi.12910) 25524563 PMC4374492

[89] Biton M, Mandelboim M, Arvatz G, Michaeli S 2006 RNAi interference of XPO1 and Sm genes and their effect on the spliced leader RNA in *Trypanosoma brucei*. Mol. Biochem. Parasitol. 150, 132–143. (10.1016/j.molbiopara.2006.07.004) 16916550

[90] De Lange T, Michels PA, Veerman HJ, Cornelissen AW, Borst P 1984 Many trypanosome messenger RNAs share a common 5′ terminal sequence. Nucleic Acids Res. 12, 3777–3790. (10.1093/nar/12.9.3777) 6547230 PMC318789

[91] Kooter J, Borst P 1984 Alpha-amanitin-insensitive transcription of variant surface glycoprotein genes provides further evidence for discontinuous transcription in trypanosomes. Nucleic Acids Res. 12, 9457–9472. (10.1093/nar/12.24.9457) 6514580 PMC320473

[92] Krchňáková Z, Krajčovič J, Vesteg M 2017 On the possibility of an early evolutionary origin for the spliced leader trans-splicing. J. Mol. Evol. 85, 37–45. (10.1007/s00239-017-9803-y) 28744787

[93] Jae N, Wang P, Gu T, Huhn M, Palfi Z, Urlaub H, Bindereif A 2010 Essential role of a trypanosome U4-specific Sm core protein in small nuclear ribonucleoprotein assembly and splicing. Eukaryot. Cell 9, 379–386. (10.1128/ec.00353-09) 20081062 PMC2837986

[94] Tkacz I, Gupta S, Volkov V, Romano M, Haham T, Tulinski P, Lebenthal I, Michaeli S 2010 Analysis of spliceosomal proteins in trypanosomatids reveals novel functions in mRNA processing. J. Biol. Chem. 285, 27 982–27 999. (10.1074/jbc.M109.095349) PMC2934664 20592024

[95] Preusser C, Palfi Z, Bindereif A 2009 Special Sm core complex functions in assembly of the U2 small nuclear ribonucleoprotein of *Trypanosoma brucei*. Eukaryot. Cell 8, 1228–1234. (10.1128/EC.00090-09) 19542313 PMC2725569

[96] Preusser C, Jae N, Bindereif A 2012 mRNA splicing in trypanosomes. Int. J. Med. Microbiol. 302, 221–224. (10.1016/j.ijmm.2012.07.004) 22964417

[97] Palfi Z et al. 2009 SMN-assisted assembly of snRNP-specific Sm cores in trypanosomes. Genes Dev. 23, 1650–1664. (10.1101/gad.526109) 19605687 PMC2714709

[98] Jae N, Preusser C, Kruger T, Tkacz ID, Engstler M, Michaeli S, Bindereif A 2011 snRNA-specific role of SMN in trypanosome snRNP biogenesis *in vivo*. RNA Biol. 8, 90–100. (10.4161/rna.8.1.13985) 21282982 PMC3127081

[99] Palfi Z, Schimanski B, Gunzl A, Lucke S, Bindereif A 2005 U1 small nuclear RNP from *Trypanosoma brucei*: a minimal U1 snRNA with unusual protein components. Nucleic Acids Res. 33, 2493–2503. (10.1093/nar/gki548) 15863726 PMC1087902

[100] Preusser C, Rossbach O, Hung LH, Li D, Bindereif A 2014 Genome-wide RNA-binding analysis of the trypanosome U1 snRNP proteins U1C and U1-70 K reveals *cis/trans*-spliceosomal network. Nucleic Acids Res. 42, 6603–6615. (10.1093/nar/gku286) 24748659 PMC4041458

[101] Hug M, Hotz HR, Hartmann C, Clayton CE 1994 Hierarchies of RNA processing signals in a trypanosome surface antigen mRNA precursor. Mol. Cell. Biol. 14, 7428–7435. (10.1128/MCB.14.11.7428) 7935457 PMC359278

[102] Schürch N, Hehl A, Vassella E, Braun R, Roditi I 1994 Accurate polyadenylation of procyclin mRNAs in *Trypanosoma brucei* is determined by pyrimidine-rich elements in the intergenic regions. Mol. Cell. Biol. 14, 3668–3675. (10.1128/MCB.14.6.3668) 7910942 PMC358734

[103] Siegel T, Hekstra D, Wang X, Dewell S, Cross G 2010 Genome-wide analysis of mRNA abundance in two life-cycle stages of *Trypanosoma brucei* and identification of splicing and polyadenylation sites. Nucleic Acids Res. 38, 4946–4957. (10.1093/nar/gkq237) 20385579 PMC2926603

[104] Smith M, Blanchette M, Papadopoulou B 2008 Improving the prediction of mRNA extremities in the parasitic protozoan *Leishmania*. BMC Bioinf. 9, 158 (10.1186/1471-2105-9-158) PMC2335281 18366710

[105] Dillon LA et al. 2015 Transcriptomic profiling of gene expression and RNA processing during *Leishmania* *major* differentiation. Nucleic Acids Res. 43, 6799–6813. (10.1093/nar/gkv656) 26150419 PMC4538839

[106] Siegel T, Tan K, Cross G 2005 Systematic study of sequence motifs for RNA *trans* splicing in *Trypanosoma brucei*. Mol. Cell. Biol. 25, 9586–9594. (10.1128/MCB.25.21.9586-9594.2005) 16227607 PMC1265811

[107] Hummel HS, Gillespie RD, Swindle J 2000 Mutational analysis of 3′ splice site selection during trans-splicing. J. Biol. Chem. 275, 35 522–35 531. (10.1074/jbc.M002424200) 10938269

[108] Patzelt E, Perry KL, Agabian N 1989 Mapping of branch sites in trans-spliced pre-mRNAs of *Trypanosoma brucei*. Mol. Cell. Biol. 9, 4291–4297. (10.1128/MCB.9.10.4291) 2479824 PMC362509

[109] Lücke S, Jürchott K, Hung L-H, Bindereif A 2005 mRNA splicing in *Trypanosoma brucei*: branch-point mapping reveals differences from the canonical U2 snRNA-mediated recognition. Mol. Biochem. Parasitol. 142, 248–251. (10.1016/j.molbiopara.2005.04.007) 15923047

[110] Chikne V et al. 2017 The canonical poly (A) polymerase PAP1 polyadenylates non-coding RNAs and is essential for snoRNA biogenesis in *Trypanosoma brucei*. J. Mol. Biol. 429, 3301–3318. (10.1016/j.jmb.2017.04.015) 28456523

[111] Koch H, Raabe M, Urlaub H, Bindereif A, Preusser C 2016 The polyadenylation complex of *Trypanosoma brucei*: characterization of the functional poly(A) polymerase. RNA Biol. 13, 221–231. (10.1080/15476286.2015.1130208) 26727667 PMC4829303

[112] Campos PC, Bartholomeu DC, DaRocha WD, Cerqueira GC, Teixeira SM 2008 Sequences involved in mRNA processing in *Trypanosoma cruzi*. Int. J. Parasitol. 38, 1383–1389. (10.1016/j.ijpara.2008.07.001) 18700146

[113] Jacobs RT et al. 2011 SCYX-7158, an orally-active benzoxaborole for the treatment of stage 2 human African trypanosomiasis. PLoS Neglected Trop. Dis. 5, e1151 (10.1371/journal.pntd.0001151) PMC3125149 21738803

[114] Akama T et al. 2017 Identification of a 4-fluorobenzyl l-valinate amide benzoxaborole (AN11736) as a potential development candidate for the treatment of animal African trypanosomiasis (AAT). Bioorg. Med. Chem. Lett. 28, 6–10. (10.1016/j.bmcl.2017.11.028) 29169674 PMC5720837

[115] Antosz W et al. 2017 The composition of the Arabidopsis RNA polymerase II transcript elongation complex reveals the interplay between elongation and mRNA processing factors. Plant Cell 29, 854–870. (10.1105/tpc.16.00735) 28351991 PMC5435424

[116] Neves LT, Douglass S, Spreafico R, Venkataramanan S, Kress TL, Johnson TL 2017 The histone variant H2A.Z promotes efficient cotranscriptional splicing in *S. cerevisiae*. Genes Dev. 31, 702–717. (10.1101/gad.295188.116) 28446598 PMC5411710

[117] Orlando TC, Mayer MG, Campbell DA, Sturm NR, Floeter-Winter LM 2007 RNA polymerase I promoter and splice acceptor site recognition affect gene expression in non-pathogenic *Leishmania* species. Mem. Inst. Oswaldo Cruz. 102, 891–894. (10.1590/S0074-02762007005000123) 18060313

[118] Stewart M, Haile S, Jha B, Cristodero M, Li C-H, Clayton C 2010 Processing of a reporter mRNA in trypanosomes is not coupled to transcription by RNA polymerase II. Mol. Biochem. Parasitol. 172, 99–106. (10.1016/j.molbiopara.2010.03.016) 20363263

[119] Ullu E, Matthews KR, Tschudi C 1993 Temporal order of RNA-processing reactions in trypanosomes: rapid *trans* splicing precedes polyadenylation of newly synthesized tubulin transcripts. Mol. Cell. Biol. 13, 720–725. (10.1128/MCB.13.1.720) 8417363 PMC358950

[120] Lombrana R, Alvarez A, Fernandez-Justel JM, Almeida R, Poza-Carrion C, Gomes F, Calzada A, Requena JM, Gomez M 2016 Transcriptionally driven DNA replication program of the human parasite *Leishmania major*. Cell Rep. 16, 1774–1786. (10.1016/j.celrep.2016.07.007) 27477279

[121] Benabdellah K, González-Rey E, González A 2007 Alternative trans-splicing of the *Trypanosoma cruzi LYT1* gene transcript results in compartmental and functional switch for the encoded protein. Mol. Microbiol. 65, 1559–1567. (10.1111/j.1365-2958.2007.05892.x) 17824931

[122] Rettig J, Wang Y, Schneider A, Ochsenreiter T 2012 Dual targeting of isoleucyl-tRNA synthetase in *Trypanosoma brucei* is mediated through alternative trans-splicing. Nucleic Acids Res. 40, 1299–1306. (10.1093/nar/gkr794) 21976735 PMC3273800

[123] Gupta SK, Carmi S, Waldman Ben-Asher H, Tkacz ID, Naboishchikov I, Michaeli S 2013 Basal splicing factors regulate the stability of mature mRNAs in trypanosomes. J. Biol. Chem. 288, 4991–5006. (10.1074/jbc.M112.416578) 23283975 PMC3576102

[124] Hartmann C, Hotz H-R, McAndrew M, Clayton C 1998 Effect of multiple downstream splice sites on polyadenylation in *Trypanosoma brucei*. Mol. Biochem. Parasitol. 93, 149–152. (10.1016/S0166-6851(98)00026-7) 9662038

[125] Lopez-Estrano C, Tschudi C, Ullu E 1998 Exonic sequences in the 5′ untranslated region of alpha-tubulin mRNA modulate trans splicing in *Trypanosoma brucei*. Mol. Cell. Biol. 18, 4620–4628. (10.1128/MCB.18.8.4620) 9671472 PMC109048

[126] Antwi E et al. 2016 Integrative analysis of the *Trypanosoma brucei* gene expression cascade predicts differential regulation of mRNA processing and unusual control of ribosomal protein expression. BMC Genomics 17, 306 (10.1186/s12864-016-2624-3) 27118143 PMC4845500

[127] Mulindwa J et al. 2018 Transcriptomes of *Trypanosoma brucei rhodesiense* from sleeping sickness patients, rodents and culture: effects of strain, growth conditions and RNA preparation methods. PLoS Neglected Trop. Dis. 12, e0006280 (10.1371/journal.pntd.0006280) PMC5842037 29474390

[128] Wasmuth EV, Lima CD 2012 Exo- and endoribonucleolytic activities of yeast cytoplasmic and nuclear RNA exosomes are dependent on the noncatalytic core and central channel. Mol. Cell 48, 133–144. (10.1016/j.molcel.2012.07.012) 22902556 PMC3472098

[129] Haile S, Cristodero M, Clayton C, Estévez A 2007 The subcellular localisation of trypanosome RRP6 and its association with the exosome. Mol. Biochem. Parasitol. 151, 52–58. (10.1016/j.molbiopara.2006.10.005) 17118470

[130] Kramer S, Piper S, Estevez A, Carrington M 2016 Polycistronic trypanosome mRNAs are a target for the exosome. Mol. Biochem. Parasitol. 205, 1–5. (10.1016/j.molbiopara.2016.02.009) 26946399 PMC4850246

[131] Kramer S, Marnef A, Standart N, Carrington M 2012 Inhibition of mRNA maturation in trypanosomes causes the formation of novel foci at the nuclear periphery containing cytoplasmic regulators of mRNA fate. J. Cell Sci. 125, 2896–2909. (10.1242/jcs.099275) 22366449 PMC3434824

[132] Zhou Z, Fu XD 2013 Regulation of splicing by SR proteins and SR protein-specific kinases. Chromosoma 122, 191–207. (10.1007/s00412-013-0407-z) 23525660 PMC3660409

[133] Anko ML 2014 Regulation of gene expression programmes by serine-arginine rich splicing factors. Semin. Cell Dev. Biol. 32, 11–21. (10.1016/j.semcdb.2014.03.011) 24657192

[134] Howard JM, Sanford JR 2015 The RNAissance family: SR proteins as multifaceted regulators of gene expression. Wiley Interdiscip. Rev. RNA 6, 93–110. (10.1002/wrna.1260) 25155147 PMC4268343

[135] Dean S, Sunter J, Wheeler R 2016 TrypTag.org: a trypanosome genome-wide protein localisation resource. Trends Parasitol. 33, 80–82. (10.1016/j.pt.2016.10.009) 27863903 PMC5270239

[136] Manger ID, Boothroyd JC 1998 Identification of a nuclear protein in *Trypanosoma brucei* with homology to RNA-binding proteins from cis-splicing systems. Mol. Biochem. Parasitol. 97, 1–11. (10.1016/S0166-6851(98)00118-2) 9879882

[137] Levy GV, Banuelos CP, Nittolo AG, Ortiz GE, Mendiondo N, Moretti G, Tekiel VS, Sanchez DO 2015 Depletion of the SR-related protein TbRRM1 leads to cell cycle arrest and apoptosis-like death in *Trypanosoma brucei*. PLoS ONE 10, e0136070 (10.1371/journal.pone.0136070) 26284933 PMC4540419

[138] Naguleswaran A, Gunasekera K, Schimanski B, Heller M, Hemphill A, Ochsenreiter T, Roditi I 2015 *Trypanosoma brucei* RRM1 is a nuclear RNA-binding protein and modulator of chromatin structure. mBio 6, e00114 (10.1128/mBio.00114-15) 25784696 PMC4453557

[139] Wippel HH, Malgarin JS, Martins ST, Vidal NM, Marcon BH, Miot HT, Marchini FK, Goldenberg S, Alves LR 2018 The nuclear RNA-binding protein RBSR1 interactome in *Trypanosoma cruzi*. J. Eukaryot. Microbiol. 66, 244–253. (10.1111/jeu.12666) 29984450

[140] Gupta SK, Chikne V, Eliaz D, Tkacz ID, Naboishchikov I, Carmi S, Waldman Ben-Asher H, Michaeli S 2014 Two splicing factors carrying serine-arginine motifs, TSR1 and TSR1IP, regulate splicing, mRNA stability, and rRNA processing in *Trypanosoma brucei*. RNA Biol. 11, 715–731. (10.4161/rna.29143) 24922194 PMC4156503

[141] De Gaudenzi JG, Frasch ACC, Clayton C 2006 RNA-binding domain proteins in kinetoplastids: a comparative analysis. Eukaryot. Cell 4, 2106–2114. (10.1128/EC.4.12.2106-2114.2005) PMC1317496 16339728

[142] Stern M, Gupta S, Salmon-Divon M, Haham T, Barda O, Levi S, Wachtel C, Nilsen T, Michaeli S 2009 Multiple roles for polypyrimidine tract binding (PTB) proteins in trypanosome RNA metabolism. RNA (New York, N.Y.) 15, 648–665. (10.1261/rna.1230209) PMC2661826 19218552

[143] Das A, Bellofatto V, Rosenfeld J, Carrington M, Romero-Zaliz R, del Val C, Estevez AM 2015 High throughput sequencing analysis of *Trypanosoma brucei* DRBD3/PTB1-bound mRNAs. Mol. Biochem. Parasitol. 199, 1–4. (10.1016/j.molbiopara.2015.02.003) 25725478

[144] Gupta SK et al. 2013 The hnRNP F/H homologue of *Trypanosoma brucei* is differentially expressed in the two life cycle stages of the parasite and regulates splicing and mRNA stability. Nucleic Acids Res. 41, 6577–6594. (10.1093/nar/gkt369) 23666624 PMC3711420

[145] Serpeloni M, Vidal NM, Goldenberg S, Avila AR, Hoffmann FG 2011 Comparative genomics of proteins involved in RNA nucleocytoplasmic export. BMC Evol. Biol. 11, 7 (10.1186/1471-2148-11-7) 21223572 PMC3032688

[146] Schwede A, Manful T, Jha B, Helbig C, Bercovich N, Stewart M, Clayton C 2009 The role of deadenylation in the degradation of unstable mRNAs in trypanosomes. Nucleic Acids Res. 37, 5511–5528. (10.1093/nar/gkp571) 19596809 PMC2760810

[147] Dostalova A, Kaser S, Cristodero M, Schimanski B 2013 The nuclear mRNA export receptor Mex67-Mtr2 of *Trypanosoma brucei* contains a unique and essential zinc finger motif. Mol. Microbiol. 88, 728–739. (10.1111/mmi.12217) 23560737

[148] Goos C, Dejung M, Wehman A, M-Natus E, Schmidt J, Sunter J, Engstler M, Butter F, Kramer S 2018 Trypanosomes can initiate nuclear export co-transcriptionally. Nucleic Acids Res. 47, 266–282. (10.1093/nar/gky1136) PMC6326799 30418648

[149] Topisirovic I, Svitkin YV, Sonenberg N, Shatkin AJ 2011 Cap and cap-binding proteins in the control of gene expression. Wiley Interdiscip. Rev. RNA 2, 277–298. (10.1002/wrna.52) 21957010

[150] Gonatopoulos-Pournatzis T, Cowling VH 2014 Cap-binding complex (CBC). Biochem. J. 457, 231–242. (10.1042/bj20131214) 24354960 PMC3901397

[151] Li H, Tschudi C 2005 Novel and essential subunits in the 300-kilodalton nuclear cap binding complex of *Trypanosoma brucei*. Mol. Cell. Biol. 25, 2216–2226. (10.1128/MCB.25.6.2216-2226.2005) 15743819 PMC1061625

[152] Eliseeva IA, Lyabin DN, Ovchinnikov LP 2013 Poly(A)-binding proteins: structure, domain organization, and activity regulation. Biochemistry (Mosc) 78, 1377–1391. (10.1134/s0006297913130014) 24490729

[153] da Costa Lima T, Moura D, Reis C, Vasconcelos J, Ellis L, Carrington M, Figueiredo R, de Melo Neto O 2010 Functional characterization of three *Leishmania* poly(A) binding protein homologues with distinct binding properties to RNA and protein partners. Eukaryot. Cell 9, 1484–1494. (10.1128/EC.00148-10) 20675580 PMC2950419

[154] Kramer S, Bannerman-Chukualim B, Ellis L, Boulden EA, Kelly S, Field MC, Carrington M 2013 Differential localization of the two *T. brucei* poly(A) binding proteins to the nucleus and RNP granules suggests binding to distinct mRNA pools. PLoS ONE 8, e54004 (10.1371/journal.pone.0054004) 23382864 PMC3559699

[155] Zoltner M, Krienitz N, Field MC, Kramer S 2018 Comparative proteomics of the two *T. brucei* PABPs suggests that PABP2 controls bulk mRNA. PLoS Neglected Trop. Dis. 12, e0006679 (10.1371/journal.pntd.0006679) PMC6075789 30040867

[156] Boehm V, Gehring NH 2016 Exon junction complexes: supervising the gene expression assembly line. Trends Genet. 32, 724–735. (10.1016/j.tig.2016.09.003) 27667727

[157] Woodward LA, Mabin JW, Gangras P, Singh G 2017 The exon junction complex: a lifelong guardian of mRNA fate. Wiley Interdiscip. Rev. RNA 8, e1411 (10.1002/wrna.1411) 28008720

[158] Gerbracht JV, Gehring NH 2018 The exon junction complex: structural insights into a faithful companion of mammalian mRNPs. Biochem. Soc. Trans. 46, 153–161. (10.1042/bst20170059) 29351963

[159] Bercovich N, Clayton C, Levin M, Vazquez M 2009 Identification of core components of the exon junction complex in trypanosomes. Mol. Biochem. Parasitol. 166, 190–193. (10.1016/j.molbiopara.2009.03.008) 19450736

[160] Dhalia R, Marinsek N, Reis C, Katz R, Muniz J, Standart N, Carrington M, de Melo Neto O 2006 The two eIF4A helicases in *Trypanosoma brucei* are functionally distinct. Nucleic Acids Res. 34, 2495–2507. (10.1093/nar/gkl290) 16687655 PMC1459412

[161] Bannerman BP, Kramer S, Dorrell RG, Carrington M 2018 Multispecies reconstructions uncover widespread conservation, and lineage-specific elaborations in eukaryotic mRNA metabolism. PLoS ONE 13, e0192633 (10.1371/journal.pone.0192633) 29561870 PMC5862402

[162] Shirokikh NE, Preiss T 2018 Translation initiation by cap-dependent ribosome recruitment: recent insights and open questions. Wiley Interdiscip. Rev. RNA 9, e1473 (10.1002/wrna.1473) 29624880

[163] Shirokikh NE, Archer SK, Beilharz TH, Powell D, Preiss T 2017 Translation complex profile sequencing to study the *in vivo* dynamics of mRNA-ribosome interactions during translation initiation, elongation and termination. Nat. Protoc. 12, 697–731. (10.1038/nprot.2016.189) 28253237

[164] Archer SK, Shirokikh NE, Beilharz TH, Preiss T 2016 Dynamics of ribosome scanning and recycling revealed by translation complex profiling. Nature 535, 570–574. (10.1038/nature18647) 27437580

[165] Archer S, Hirokikh N, Hallwirth C, Beilharz T, Preiss T 2015 Probing the closed-loop model of mRNA translation in living cells. RNA Biol. 12, 248–254. (10.1080/15476286.2015.1017242) 25826658 PMC4615164

[166] Khong A, Parker R 2018 mRNP architecture in translating and stress conditions reveals an ordered pathway of mRNP compaction. J. Cell Biol. 217, 4124–4140. (10.1083/jcb.201806183) 30322972 PMC6279387

[167] Erben E, Fadda A, Lueong S, Hoheisel J, Clayton C 2014 Genome-wide discovery of post-transcriptional regulators in *Trypanosoma brucei*. PLoS Pathog. 10, e1004178 (10.1371/journal.ppat.1004178) 24945722 PMC4055773

[168] Zinoviev A, Leger M, Wagner G, Shapira M 2011 A novel 4E-interacting protein in *Leishmania* is involved in stage-specific translation pathways. Nucleic Acids Res. 39, 8404–8415. (10.1093/nar/gkr555) 21764780 PMC3201875

[169] Yoffe Y et al. 2004 Cap-binding activity of an eIF4E homolog from *Leishmania*. RNA (New York, N.Y.) 10, 1764–1775. (10.1261/rna.7520404) PMC1370664 15388875

[170] Yoffe Y, Zuberek J, Lerer A, Lewdorowicz M, Stepinski J, Altmann M, Darzynkiewicz E, Shapira M 2006 Binding specificities and potential roles of isoforms of eukaryotic initiation factor 4E in *Leishmania*. Eukaryot. Cell 5, 1969–1979. (10.1128/EC.00230-06) 17041189 PMC1694823

[171] Terrao M, Kamanyi Marucha K, Mugo E, Droll D, Minia I, Egler F, Braun J, Clayton C 2018 The suppressive cap-binding-complex factor 4EIP is required for normal differentiation. Nucleic Acids Res. 46, 8993–9010. (10.1093/nar/gky733) 30124912 PMC6158607

[172] Freire ER et al. 2018 *Trypanosoma brucei* EIF4E2 cap-binding protein binds a homolog of the histone-mRNA stem-loop-binding protein. Curr. Genet. 64, 821–839. (10.1007/s00294-017-0795-3) 29288414

[173] Yoffe Y, Leger M, Zinoviev A, Zuberek J, Darzynkiewicz E, Wagner G, Shapira M 2009 Evolutionary changes in the *Leishmania* eIF4F complex involve variations in the eIF4E-eIF4G interactions. Nucleic Acids Res. 37, 3243–3253. (10.1093/nar/gkp190) 19321500 PMC2691829

[174] Freire E et al. 2011 The four trypanosomatid eIF4E homologues fall into two separate groups, with distinct features in primary sequence and biological properties. Mol. Biochem. Parasitol. 176, 25–36. (10.1016/j.molbiopara.2010.11.011) 21111007 PMC6736675

[175] Freire ER et al. 2014 eIF4F-like complexes formed by cap-binding homolog TbEIF4E5 with TbEIF4G1 or TbEIF4G2 are implicated in post-transcriptional regulation in *Trypanosoma brucei*. RNA (New York, N.Y.) 20, 1272–1286. (10.1261/rna.045534.114) PMC4105752 24962368

[176] Freire E et al. 2014 *Trypanosoma brucei* translation-initiation factor homolog EIF4E6 forms a tripartite cytosolic complex with EIF4G5 and a capping enzyme homolog. Eukaryot. Cell 13, 896–908. (10.1128/EC.00071-14) 24839125 PMC4135740

[177] Freire ER, Sturm NR, Campbell DA, de Melo Neto OP 2017 The role of cytoplasmic mRNA cap-binding protein complexes in *Trypanosoma brucei* and other trypanosomatids. Pathogens 6, 55 (10.3390/pathogens6040055) PMC5750579 29077018

[178] Klein C, Terrao M, Inchaustegui Gil D, Clayton C 2015 Polysomes of *Trypanosoma brucei*: association with initiation factors and RNA-binding proteins. PLoS ONE 10, e0135973 (10.1371/journal.pone.0135973) 26287607 PMC4545788

[179] Dos Santos Rodrigues FH, Firczuk H, Breeze AL, Cameron AD, Walko M, Wilson AJ, Zanchin NIT, McCarthy JEG 2018 The *Leishmania* PABP1-eIF4E4 interface: a novel 5′-3′ interaction architecture for trans-spliced mRNAs. Nucleic Acids Res. 47, 1493–1504. (10.1093/nar/gky1187) PMC6379680 30476241

[180] Moura DM, Reis CR, Xavier CC, da Costa Lima TD, Lima RP, Carrington M, de Melo Neto OP 2015 Two related trypanosomatid eIF4G homologues have functional differences compatible with distinct roles during translation initiation. RNA Biol. 12, 305–319. (10.1080/15476286.2015.1017233) 25826663 PMC4615764

[181] Dejung M, Subota I, Bucerius F, Dindar G, Freiwald A, Engstler M, Boshart M, Butter F, Janzen CJ 2016 Quantitative proteomics uncovers novel factors involved in developmental dfferentiation of *Trypanosoma brucei*. PLoS Pathog. 12, e1005439 (10.1371/journal.ppat.1005439) 26910529 PMC4765897

[182] Meleppattu S, Arthanari H, Zinoviev A, Boeszoermenyi A, Wagner G, Shapira M, Leger-Abraham M 2018 Structural basis for LeishIF4E-1 modulation by an interacting protein in the human parasite *Leishmania major*. Nucleic Acids Res. 46, 3791–3801. (10.1093/nar/gky194) 29562352 PMC5909430

[183] Choe J et al. 2014 eIF4AIII enhances translation of nuclear cap-binding complex-bound mRNAs by promoting disruption of secondary structures in 5′UTR. Proc. Natl Acad. Sci. USA 111, E4577–E4586. (10.1073/pnas.1409695111) 25313076 PMC4217471

[184] Kim KM, Cho H, Choi K, Kim J, Kim BW, Ko YG, Jang SK, Kim YK 2009 A new MIF4G domain-containing protein, CTIF, directs nuclear cap-binding protein CBP80/20-dependent translation. Genes Dev. 23, 2033–2045. (10.1101/gad.1823409) 19648179 PMC2751978

[185] Parsyan A, Svitkin Y, Shahbazian D, Gkogkas C, Lasko P, Merrick WC, Sonenberg N 2011 mRNA helicases: the tacticians of translational control. Nat. Rev. Mol. Cell. Biol. 12, 235–245. (10.1038/nrm3083) 21427765

[186] Bourgeois C, Mortreux F, Auboeuf D 2016 The multiple functions of RNA helicases as drivers and regulators of gene expression. Nat. Rev. Mol. Cell. Biol. 17, 426–438. (10.1038/nrm.2016.50) 27251421

[187] Hinnebusch AG, Ivanov IP, Sonenberg N 2016 Translational control by 5′-untranslated regions of eukaryotic mRNAs. Science 352, 1413–1416. (10.1126/science.aad9868) 27313038 PMC7422601

[188] Guenther UP et al. 2018 The helicase Ded1p controls use of near-cognate translation initiation codons in 5′ UTRs. Nature 559, 130–134. (10.1038/s41586-018-0258-0) 29950728 PMC6226265

[189] Chavez S et al. 2017 Transcriptome-wide analysis of the *Trypanosoma cruzi* proliferative cycle identifies the periodically expressed mRNAs and their multiple levels of control. PLoS ONE 12, e0188441 (10.1371/journal.pone.0188441) 29182646 PMC5705152

[190] Delhi P, Queiroz R, Inchaustegui D, Carrington M, Clayton C 2011 Is there a classical nonsense-mediated decay pathway in trypanosomes? PLoS ONE 6, e25112 (10.1371/journal.pone.0025112) 21957477 PMC3177853

[191] Zinoviev A, Akum Y, Yahav T, Shapira M 2012 Gene duplication in trypanosomatids—two DED1 paralogs are functionally redundant and differentially expressed during the life cycle. Mol. Biochem. Parasitol. 185, 127–136. (10.1016/j.molbiopara.2012.08.001) 22910033

[192] Andreev DE, O'Connor PB, Loughran G, Dmitriev SE, Baranov PV, Shatsky IN 2017 Insights into the mechanisms of eukaryotic translation gained with ribosome profiling. Nucleic Acids Res. 45, 513–526. (10.1093/nar/gkw1190) 27923997 PMC5314775

[193] Jensen BC, Ramasamy G, Vasconcelos EJ, Ingolia NT, Myler PJ, Parsons M 2014 Extensive stage-regulation of translation revealed by ribosome profiling of *Trypanosoma brucei*. BMC Genomics 15, 911 (10.1186/1471-2164-15-911) 25331479 PMC4210626

[194] Vasquez JJ, Hon CC, Vanselow JT, Schlosser A, Siegel TN 2014 Comparative ribosome profiling reveals extensive translational complexity in different *Trypanosoma brucei* life cycle stages. Nucleic Acids Res. 42, 3623–3637. (10.1093/nar/gkt1386) 24442674 PMC3973304

[195] Hanson G, Coller J 2018 Codon optimality, bias and usage in translation and mRNA decay. Nat. Rev. Mol. Cell. Biol. 19, 20–30. (10.1038/nrm.2017.91) 29018283 PMC6594389

[196] de Freitas Nascimento J, Kelly S, Sunter J, Carrington M 2018 Codon choice directs constitutive mRNA levels in trypanosomes. Elife 7, e32467 (10.7554/eLife.32467) 29543152 PMC5896880

[197] Jeacock L, Faria J, Horn D 2018 Codon usage bias controls mRNA and protein abundance in trypanosomatids. Elife 7, e32496 (10.7554/eLife.32496) 29543155 PMC5896881

[198] Kalyna M et al. 2012 Alternative splicing and nonsense-mediated decay modulate expression of important regulatory genes in Arabidopsis. Nucleic Acids Res. 40, 2454–2469. (10.1093/nar/gkr932) 22127866 PMC3315328

[199] Kim YK, Maquat LE 2019 UPFront and center in RNA decay: UPF1 in nonsense-mediated mRNA decay and beyond. RNA (New York, N.Y.) 25, 407–422. (10.1261/rna.070136.118) PMC6426291 30655309

[200] Alsford S, Turner D, Obado S, Sanchez-Flores A, Glover L, Berriman M, Hertz-Fowler C, Horn D 2011 High throughput phenotyping using parallel sequencing of RNA interference targets in the African trypanosome. Genome Res. 21, 915–924. (10.1101/gr.115089.110) 21363968 PMC3106324

[201] Collart MA, Panasenko OO 2017 The Ccr4-Not complex: architecture and structural Insights. Subcell. Biochem. 83, 349–379. (10.1007/978-3-319-46503-6_13) 28271483

[202] Ukleja M, Cuellar J, Siwaszek A, Kasprzak JM, Czarnocki-Cieciura M, Bujnicki JM, Dziembowski A, Valpuesta JM 2016 The architecture of the *Schizosaccharomyces pombe* CCR4-NOT complex. Nat. Commun. 7, 10433 (10.1038/ncomms10433) 26804377 PMC4737751

[203] Yan YB 2014 Deadenylation: enzymes, regulation, and functional implications. Wiley Interdiscip. Rev. RNA 5, 421–443. (10.1002/wrna.1221) 24523229

[204] Schwede A, Ellis L, Luther J, Carrington M, Stoecklin G, Clayton C 2008 A role for Caf1 in mRNA deadenylation and decay in trypanosomes and human cells. Nucleic Acids Res. 36, 3374–3388. (10.1093/nar/gkn108) 18442996 PMC2425496

[205] Erben E, Chakraborty C, Clayton C 2014 The CAF1-NOT complex of trypanosomes. Front. Genetics Res. Top. 4, 299 (10.3389/fgene.2013.00299) PMC3877767 24427169

[206] Fadda A, Färber V, Droll D, Clayton C 2013 The roles of 3′-exoribonucleases and the exosome in trypanosome mRNA degradation. RNA (New York, N.Y.) 19, 937–947. (10.1261/rna.038430.113) PMC3683928 23697549

[207] Färber V, Erben E, Sharma S, Stoecklin G, Clayton C 2013 Trypanosome CNOT10 is essential for the integrity of the NOT deadenylase complex and for degradation of many mRNAs. Nucleic Acids Res. 41, 1211–1222. (10.1093/nar/gks1133) 23221646 PMC3553956

[208] Jonas S, Christie M, Peter D, Bhandari D, Loh B, Huntzinger E, Weichenrieder O, Izaurralde E 2014 An asymmetric PAN3 dimer recruits a single PAN2 exonuclease to mediate mRNA deadenylation and decay. Nat. Struct. Mol. Biol. 21, 599–608. (10.1038/nsmb.2837) 24880343

[209] Schafer IB, Rode M, Bonneau F, Schussler S, Conti E 2014 The structure of the Pan2-Pan3 core complex reveals cross-talk between deadenylase and pseudokinase. Nat. Struct. Mol. Biol. 21, 591–598. (10.1038/nsmb.2834) 24880344

[210] Yi H, Park J, Ha M, Lim J, Chang H, Kim V 2018 PABP cooperates with the CCR4-NOT complex to promote mRNA deadenylation and block precocious decay. Mol. Cell 70, 1081–1088. (10.1016/j.molcel.2018.05.009) 29932901

[211] Utter C, Garcia S, Milone J, Bellofatto V 2011 Poly(A)-specific ribonuclease (PARN-1) function in stage-specific mRNA turnover in *Trypanosoma brucei*. Eukaryot. Cell 10, 1230–1240. (10.1128/EC.05097-11) 21743004 PMC3187051

[212] Lima SA, Chipman LB, Nicholson AL, Chen YH, Yee BA, Yeo GW, Coller J, Pasquinelli AE 2017 Short poly(A) tails are a conserved feature of highly expressed genes. Nat. Struct. Mol. Biol. 24, 1057–1063. (10.1038/nsmb.3499) 29106412 PMC5877826

[213] Jha B, Fadda A, Merce C, Mugo E, Droll D, Clayton C 2014 Depletion of the trypanosome pumilio domain protein PUF2 causes transcriptome changes related to coding region length. Eukaryot. Cell 13, 664–674. (10.1128/EC.00018-14) 24681684 PMC4060476

[214] Hu W, Sweet T, Chamnongpol S, Baker K, Coller J 2009 Co-translational mRNA decay in *Saccharomyces cerevisiae*. Nature 461, 225–229. (10.1038/nature08265) 19701183 PMC2745705

[215] Mugridge JS, Coller J, Gross JD 2018 Structural and molecular mechanisms for the control of eukaryotic 5′–3′ mRNA decay. Nat. Struct. Mol. Biol. 25, 1077–1085. (10.1038/s41594-018-0164-z) 30518847

[216] Kramer S 2017 The ApaH-like phosphatase TbALPH1 is the major mRNA decapping enzyme of trypanosomes. PLoS Pathog. 13, e1006456 (10.1371/journal.ppat.1006456) 28628654 PMC5491325

[217] Manful T, Fadda A, Clayton C 2011 The role of the 5′-3′ exoribonuclease XRNA in transcriptome-wide mRNA degradation. RNA (New York, N.Y.) 17, 2039–2047. (10.1261/rna.2837311) PMC3198596 21947264

[218] Ignatochkina AV, Takagi Y, Liu Y, Nagata K, Ho CK 2015 The messenger RNA decapping and recapping pathway in *Trypanosoma*. Proc. Natl Acad. Sci. USA 112, 6967–6972. (10.1073/pnas.1424909112) 26038549 PMC4460440

[219] Milone J, Wilusz J, Bellofatto V 2002 Identification of mRNA decapping activities and an ARE-regulated 3′ to 5′ exonuclease activity in trypanosome extracts. Nucleic Acids Res. 30, 4040–4050. (10.1093/nar/gkf521) 12235388 PMC137106

[220] Kramer S 2017 Simultaneous detection of mRNA transcription and decay intermediates by dual colour single mRNA FISH on subcellular resolution. Nucleic Acids Res. 45, e49 (10.1093/nar/gkw1245) 27940558 PMC5397161

[221] Irmer H, Clayton CE 2001 Degradation of the *EP1* mRNA in *Trypanosoma brucei* is initiated by destruction of the 3′-untranslated region. Nucleic Acids Res. 29, 4707–4715. (10.1093/nar/29.22.4707) 11713321 PMC92565

[222] Haile S, Estévez AM, Clayton C 2003 A role for the exosome in the initiation of degradation of unstable mRNAs. RNA (New York, N.Y.) 9, 1491–1501. (10.1261/rna.5940703) PMC1370503 14624005

[223] Sweet T, Kovalak C, Coller J 2012 The DEAD-Box protein Dhh1 promotes decapping by slowing ribosome movement. PLoS Biol. 10, e1001342 (10.1371/journal.pbio.1001342) 22719226 PMC3373615

[224] Kramer S, Queiroz R, Ellis L, Hoheisel J, Clayton C, Carrington M 2010 The RNA helicase DHH1 is central to correct expression of many developmentally regulated mRNAs in trypanosomes. J. Cell Sci. 123, 699–711. (10.1242/jcs.058511) 20124414 PMC2823576

[225] Brecht M, Parsons M 1998 Changes in polysome profiles accompany trypanosome development. Mol. Biochem. Parasitol. 97, 189–198. (10.1016/S0166-6851(98)00151-0) 9879897

[226] Pays E, Hanocq-Quertier J, Hanocq F, Van Assel S, Nolan D, Rolin S 1993 Abrupt RNA changes precede the first cell division during the differentiation of *Trypanosoma brucei* bloodstream forms into procyclic forms *in vitro*. Mol. Biochem. Parasitol. 61, 107–114. (10.1016/0166-6851(93)90163-R) 8259123

[227] Vassella E, Den Abbeele J, Butikofer P, Renggli CK, Furger A, Brun R, Roditi I 2000 A major surface glycoprotein of *Trypanosoma brucei* is expressed transiently during development and can be regulated post-transcriptionally by glycerol or hypoxia. Genes Dev. 14, 615–626. 10716949 PMC316419

[228] Vassella E, Probst M, Schneider A, Studer E, Renggli C, Roditi I 2004 Expression of a major surface protein of *Trypanosoma brucei* insect forms is controlled by the activity of mitochondrial enzymes. Mol. Biol. Cell 15, 3986–3993. (10.1091/mbc.e04-04-0341) 15201340 PMC515333

[229] Lamour N, Riviere L, Coustou V, Coombs GH, Barrett MP, Bringaud F 2005 Proline metabolism in procyclic *Trypanosoma brucei* is down-regulated in the presence of glucose. J. Biol. Chem. 280, 11 902–11 910. (10.1074/jbc.M414274200) 15665328

[230] Coustou V et al. 2008 Glucose-induced remodeling of intermediary and energy metabolism in procyclic *Trypanosoma brucei*. J. Biol. Chem. 283, 16 342–16 354. (10.1074/jbc.M709592200) 18430732

[231] Bauer S, Morris JC, Morris MT 2013 Environmentally regulated glycosome protein composition in the African trypanosome. Eukaryot. Cell 12, 1072–1079. (10.1128/ec.00086-13) 23709182 PMC3754536

[232] Imhof S, Knusel S, Gunasekera K, Vu XL, Roditi I 2014 Social motility of African trypanosomes is a property of a distinct life-cycle stage that occurs early in tsetse fly transmission. PLoS Pathog. 10, e1004493 (10.1371/journal.ppat.1004493) 25357194 PMC4214818

[233] Qiu Y, Milanes JE, Jones JA, Noorai RE, Shankar V, Morris JC 2018 Glucose signaling is important for nutrient adaptation during differentiation of pleomorphic African Trypanosomes. mSphere 3, e00366-18 (10.1128/mSphere.00366-18) 30381351 PMC6211221

[234] Pineda E et al. 2018 Glycerol supports growth of the *Trypanosoma brucei* bloodstream forms in the absence of glucose: analysis of metabolic adaptations on glycerol-rich conditions. PLoS Pathog. 14, e1007412 (10.1371/journal.ppat.1007412) 30383867 PMC6245841

[235] Benz C, Lo W, Fathallah N, Connor-Guscott A, Benns HJ, Urbaniak MD 2018 Dynamic regulation of the *Trypanosoma brucei* transferrin receptor in response to iron starvation is mediated via the 3′UTR. PLoS ONE 13, e0206332 (10.1371/journal.pone.0206332) 30596656 PMC6312234

[236] Xiao Y, Nguyen S, Kim SH, Volkov OA, Tu BP, Phillips MA 2013 Product feedback regulation implicated in translational control of the *Trypanosoma brucei* S-adenosylmethionine decarboxylase regulatory subunit prozyme. Mol. Microbiol. 88, 846–861. (10.1111/mmi.12226) 23634831 PMC3771639

[237] Fernandez-Moya SM, Carrington M, Estevez AM 2014 A short RNA stem-loop is necessary and sufficient for repression of gene expression during early logarithmic phase in trypanosomes. Nucleic Acids Res. 42, 7201–7209. (10.1093/nar/gku358) 24813448 PMC4066783

[238] Kolev NG, Ullu E, Tschudi C 2014 The emerging role of RNA-binding proteins in the life cycle of *Trypanosoma brucei*. Cell. Microbiol. 16, 482–489. (10.1111/cmi.12268) 24438230 PMC3974610

[239] Lye L-F, Owens K, Shi H, Murta S, Vieira A, Turco S, Tschudi C, Ullu E, Beverley S 2010 Retention and loss of RNA interference pathways in Trypanosomatid protozoans. PLoS Pathog. 6, e1001161 (10.1371/journal.ppat.1001161) 21060810 PMC2965760

[240] Michaeli S, Doniger T, Gupta SK, Wurtzel O, Romano M, Visnovezky D, Sorek R, Unger R, Ullu E 2012 RNA-seq analysis of small RNPs in *Trypanosoma brucei* reveals a rich repertoire of non-coding RNAs. Nucleic Acids Res. 40, 1282–1298. (10.1093/nar/gkr786) 21976736 PMC3273796

[241] Linker K, Pautz A, Fechir M, Hubrich T, Greeve J, Kleinert H 2005 Involvement of KSRP in the post-transcriptional regulation of human iNOS expression-complex interplay of KSRP with TTP and HuR. Nucleic Acids Res. 33, 4813–4827. (10.1093/nar/gki797) 16126846 PMC1192834

[242] Cao H 2004 Expression, purification, and biochemical characterization of the antiinflammatory tristetraprolin: a zinc-dependent mRNA binding protein affected by posttranslational modifications. Biochemistry 43, 13 724–13 738. (10.1021/bi049014y) PMC1351390 15504035

[243] Banerjee H, Rahn A, Davis W, Singh R 2003 Sex lethal and U2 small nuclear ribonucleoprotein auxiliary factor (U2AF^65^) recognize polypyrimidine tracts using multiple modes of binding. RNA (New York, N.Y.) 9, 88–99. (10.1261/rna.2131603) PMC1370373 12554879

[244] Jenkins HT, Malkova B, Edwards TA 2011 Kinked beta-strands mediate high-affinity recognition of mRNA targets by the germ-cell regulator DAZL. Proc. Natl Acad. Sci. USA 108, 18 266–18 271. (10.1073/pnas.1105211108) 22021443 PMC3215079

[245] Archer S, Inchaustegui D, de Queiroz R, Clayton C 2011 The cell-cycle regulated transcriptome of an early-branching eukaryote. PLoS ONE 6, e18425 (10.1371/journal.pone.0018425) 21483801 PMC3069104

[246] Mahmood R, Hines JC, Ray DS 1999 Identification of *cis* and *trans* elements involved in the cell cycle regulation of multiple genes in *Crithidia fasciculata*. Mol. Cell. Biol. 19, 6174–6182. (10.1128/MCB.19.9.6174) 10454564 PMC84551

[247] Alves L, Oliveira C, Morking P, Kessler R, Martins S, Romagnoli B, Marchini F, Goldenberg S 2014 The mRNAs associated to a zinc finger protein from *Trypanosoma cruzi* shift during stress conditions. RNA Biol. 11, 921–933. (10.4161/rna.29622) 25180711 PMC4179965

[248] Mittra B, Ray DS 2004 Presence of a poly(A) binding protein and two proteins with cell cycle-dependent phosphorylation in *Crithidia fasciculata* mRNA cycling sequence binding protein II. Eukaryot. Cell 3, 1185–1197. (10.1128/EC.3.5.1185-1197.2004) 15470247 PMC522618

[249] Archer SK, van Luu D, de Queiroz R, Brems S, Clayton CE 2009 *Trypanosoma brucei* PUF9 regulates mRNAs for proteins involved in replicative processes over the cell cycle. PLoS Pathog. 5, e1000565 (10.1371/journal.ppat.1000565) 19714224 PMC2727004

[250] Goldstrohm AC, Hall TMT, McKenney KM 2018 Post-transcriptional regulatory functions of mammalian Pumilio proteins. Trends Genet. 34, 972–990. (10.1016/j.tig.2018.09.006) 30316580 PMC6251728

[251] Azizi H, Dumas C, Papadopoulou B 2017 The Pumilio-domain protein PUF6 contributes to SIDER2 retroposon-mediated mRNA decay in *Leishmania*. RNA (New York, N.Y.) 23, 1874–1885. (10.1261/rna.062950.117) PMC5689007 28877997

[252] Mugo E, Clayton C 2017 Expression of the RNA-binding protein RBP10 promotes the bloodstream-form differentiation state in *Trypanosoma brucei*. PLoS Pathog. 13, e1006560 (10.1371/journal.ppat.1006560) 28800584 PMC5568443

[253] Urbaniak MD, Guther ML, Ferguson MA 2012 Comparative SILAC proteomic analysis of *Trypanosoma brucei* bloodstream and procyclic lifecycle stages. PLoS ONE 7, e36619 (10.1371/journal.pone.0036619) 22574199 PMC3344917

[254] Butter F, Bucerius F, Michel M, Cicova Z, Mann M, Janzen C 2012 Comparative proteomics of two life cycle stages of stable isotope-labeled *Trypanosoma brucei* reveals novel components of the parasite's host adaptation machinery. Mol. Cell. Proteomics: MCP 12, 172–179. (10.1074/mcp.M112.019224) 23090971 PMC3536898

[255] Christiano R, Kolev N, Shi H, Ullu E, Walther T, Tschudi C 2017 The proteome and transcriptome of the infectious metacyclic form of *Trypanosoma brucei* define quiescent cells primed for mammalian invasion. Mol. Microbiol. 106, 74–92. (10.1111/mmi.13754) 28742275 PMC5607103

[256] Wurst M, Selinger B, Jha B, Klein C, Queiroz R, Clayton C 2012 Expression of the RNA recognition motif protein RBP10 promotes a bloodstream-form transcript pattern in *Trypanosoma brucei*. Mol. Microbiol. 83, 1048–1063. (10.1111/j.1365-2958.2012.07988.x) 22296558

[257] Miguel De Pablos L, Kelly S, Nascimento J, Sunter J, Carrington M 2017 Characterization of RBP9 and RBP10, two developmentally regulated RNA binding proteins in *Trypanosoma brucei*. Open Biol. 7, 160159 (10.1098/rsob.160159) 28381627 PMC5413900

[258] Wippel HH et al. 2018 Assessing the partners of RBP9-mRNP complex in *Trypanosoma cruzi* using shotgun proteomics and RNA-seq. RNA Biol. 15, 1106–1118. (10.1080/15476286.2018.1509660) 30146924 PMC6161725

[259] Klein C, Terrao M, Clayton C 2017 The role of the zinc finger protein ZC3H32 in bloodstream-form *Trypanosoma brucei*. PLoS ONE 12, e0177901 (10.1371/journal.pone.0177901) 28545140 PMC5435347

[260] Chan LY, Mugler CF, Heinrich S, Vallotton P, Weis K 2018 Non-invasive measurement of mRNA decay reveals translation initiation as the major determinant of mRNA stability. Elife 7, e32536 (10.7554/eLife.32536) 30192227 PMC6152797

[261] Estévez A 2008 The RNA-binding protein *Tb*DRBD3 regulates the stability of a specific subset of mRNAs in trypanosomes. Nucleic Acids Res. 36, 4573–4586. (10.1093/nar/gkn406) 18611951 PMC2504296

[262] Paterou A, Walrad P, Craddy P, Fenn K, Matthews K 2006 Identification and stage-specific association with the translational apparatus of TbZFP3, a CCCH protein that promotes trypanosome life cycle development. J. Biol. Chem. 281, 39 002–39 013. (10.1074/jbc.M604280200) PMC2688685 17043361

[263] Hendriks EF, Robinson DR, Hinkins M, Matthews KR 2001 A novel CCCH protein which modulates differentiation of *Trypanosoma brucei* to its procyclic form. EMBO J. 20, 6700–6711. (10.1093/emboj/20.23.6700) 11726506 PMC125759

[264] Walrad P, Paterou A, Acosta-Serrano A, Matthews K 2009 Differential trypanosome surface coat regulation by a CCCH protein that co-associates with *procyclin* mRNA *cis*-elements. PLoS Pathog. 5, e1000317 (10.1371/journal.ppat.1000317) 19247446 PMC2642730

[265] Walrad P, Capewell P, Fenn K, Matthews K 2011 The post-transcriptional trans-acting regulator, TbZFP3, co-ordinates transmission-stage enriched mRNAs in *Trypanosoma brucei*. Nucleic Acids Res. 40, 2869–2883. (10.1093/nar/gkr1106) 22140102 PMC3326296

[266] Rico E, Ivens A, Glover L, Horn D, Matthews KR 2017 Genome-wide RNAi selection identifies a regulator of transmission stage-enriched gene families and cell-type differentiation in *Trypanosoma brucei*. PLoS Pathog. 13, e1006279 (10.1371/journal.ppat.1006279) 28334017 PMC5380359

[267] Ling A, Trotter J, Hendriks E 2011 A zinc finger protein, TbZC3H20, stabilises two developmentally regulated mRNAs in trypanosomes. J. Biol. Chem. 286, 20 152–20 162. (10.1074/jbc.M110.139261) PMC3121479 21467035

[268] Knusel S, Roditi I 2013 Insights into the regulation of GPEET procyclin during differentiation from early to late procyclic forms of *Trypanosoma brucei*. Mol. Biochem. Parasitol. 191, 66–74. (10.1016/j.molbiopara.2013.09.004) 24076427

[269] Viegas SC, Silva IJ, Apura P, Matos RG, Arraiano CM 2015 Surprises in the 3′-end: ‘U’ can decide too! FEBS J. 282, 3489–3499. (10.1111/febs.13377) 26183531

[270] Berberof M, Vanhamme L, Tebabi P, Pays A, Jefferies D, Welburn S, Pays E 1995 The 3′-terminal region of the mRNAs for VSG and procyclin can confer stage specificity to gene expression in *T. brucei*. EMBO J. 14, 2925–2934. (10.1002/j.1460-2075.1995.tb07292.x) 7796818 PMC398412

[271] Webb H, Burns R, Kimblin N, Ellis L, Carrington M 2005 A novel strategy to identify the location of necessary and sufficient cis-acting regulatory mRNA elements in trypanosomes. RNA (New York, N.Y.) 11, 1108–1116. (10.1261/rna.2510505) PMC1360220 15928343

[272] Colasante C, Robles A, Li C-H, Schwede A, Benz C, Voncken F, Guilbride DL, Clayton C 2007 Regulated expression of glycosomal phosphoglycerate kinase in *Trypanosoma brucei*. Mol. Biochem. Parasitol. 151, 193–204. (10.1016/j.molbiopara.2006.11.003) 17187872

[273] Jojic B, Amodeo S, Bregy I, Ochsenreiter T 2018 Distinct 3′ UTRs regulate the life-cycle-specific expression of two TCTP paralogs in *Trypanosoma brucei*. J. Cell Sci. 131, jcs206417 (10.1242/jcs.206417) 29661850 PMC5992589

[274] Telleria EL, Benoit JB, Zhao X, Savage AF, Regmi S, Alves e Silva TL, O'Neill M, Aksoy S 2014 Insights into the trypanosome-host interactions revealed through transcriptomic analysis of parasitized tsetse fly salivary glands. PLoS Neglected Trop. Dis. 8, e2649 (10.1371/journal.pntd.0002649) PMC3998935 24763140

[275] Savage AF, Kolev NG, Franklin JB, Vigneron A, Aksoy S, Tschudi C 2016 Transcriptome profiling of *Trypanosoma brucei* development in the Tsetse fly vector *Glossina morsitans*. PLoS ONE 11, e0168877 (10.1371/journal.pone.0168877) 28002435 PMC5176191

[276] Kolev NG, Ramey-Butler K, Cross GA, Ullu E, Tschudi C 2012 Developmental progression to infectivity in *Trypanosoma brucei* triggered by an RNA-binding protein. Science 338, 1352–1353. (10.1126/science.1229641) 23224556 PMC3664091

[277] Shi H, Ramey-Butler K, Tschudi C 2018 A single-point mutation in the RNA-binding protein 6 generates *Trypanosoma brucei* metacyclics that are able to progress to bloodstream forms *in vitro*. Mol. Biochem. Parasitol. 224, 50–56. (10.1016/j.molbiopara.2018.07.011) 30055184 PMC6147148

[278] Shi H, Butler K, Tschudi C 2018 Differential expression analysis of transcriptome data of *Trypanosoma brucei* RBP6 induction in procyclics leading to infectious metacyclics and bloodstream forms *in vitro*. Data Brief. 20, 978–980. (10.1016/j.dib.2018.08.169) 30225310 PMC6139001

[279] McDonald L, Cayla M, Ivens A, Mony BM, MacGregor P, Silvester E, McWilliam K, Matthews KR 2018 Non-linear hierarchy of the quorum sensing signalling pathway in bloodstream form African trypanosomes. PLoS Pathog. 14, e1007145 (10.1371/journal.ppat.1007145) 29940034 PMC6034907

[280] Najafabadi HS, Lu Z, MacPherson C, Mehta V, Adoue V, Pastinen T, Salavati R 2013 Global identification of conserved post-transcriptional regulatory programs in trypanosomatids. Nucleic Acids Res. 41, 8591–8600. (10.1093/nar/gkt647) 23877242 PMC3794602

[281] Jha BA, Gazestani VH, Yip CW, Salavati R 2015 The DRBD13 RNA binding protein is involved in the insect-stage differentiation process of *Trypanosoma brucei*. FEBS Lett. 589, 1966–1974. (10.1016/j.febslet.2015.05.036) 26028502

[282] Mani J, Güttinger A, Schimanski B, Heller M, Acosta-Serrano A, Pescher P, Späth G, Sabel Roditi I 2011 Alba-domain proteins of *Trypanosoma brucei* are cytoplasmic RNA-binding proteins that interact with the translation machinery. PLoS ONE 6, e22463 (10.1371/journal.pone.0022463) 21811616 PMC3141063

[283] Hartmann C et al. 2007 The small trypanosome RNA-binding proteins *Tb*UBP1 and *Tb*UBP2 influence expression of F box protein mRNAs in bloodstream trypanosomes. Eukaryot. Cell 6, 1964–1978. (10.1128/EC.00279-07) 17873084 PMC2168414

[284] De Gaudenzi JG, D'Orso I, Frasch AC 2003 RNA recognition motif-type RNA-binding proteins in *Trypanosoma cruzi* form a family involved in the interaction with specific transcripts *in vivo*. J. Biol. Chem. 278, 18 884–18 894. (10.1074/jbc.M301756200) 12637517

[285] D'Orso I, Frasch ACC 2002 TcUBP-1, an mRNA destabilizing factor from trypanosomes, homodimerizes and interacts with novel AU-rich element- and poly(A)-binding proteins forming a ribonucleoprotein complex. J. Biol. Chem. 277, 50 520–50 528. (10.1074/jbc.M209092200) 12403777

[286] Cassola A, Frasch A 2009 An RNA recognition motif mediates the nucleocytoplasmic transport of a trypanosome RNA-binding protein. J. Biol. Chem. 284, 35 015–35 028. (10.1074/jbc.M109.031633) PMC2787363 19801539

[287] Hartmann C, Clayton C 2008 Regulation of a transmembrane protein gene family by the small RNA binding proteins *Tb*UBP1 and *Tb*UBP2. Mol. Biochem. Parasitol. 157, 112–115. (10.1016/j.molbiopara.2007.09.009) 18022708

[288] Noe G, De Gaudenzi J, Frasch A 2008 Functionally related transcripts have common RNA motifs for specific RNA-binding proteins in trypanosomes. BMC Mol. Biol. 9, 107 (10.1186/1471-2199-9-107) 19063746 PMC2637893

[289] Wurst M et al. 2009 An RNAi screen of the RRM-domain proteins of *Trypanosoma brucei*. Mol. Biochem. Parasitol. 163, 61–65. (10.1016/j.molbiopara.2008.09.001) 18840477

[290] Das A, Morales R, Banday M, Garcia S, Hao L, Cross GA, Estevez AM, Bellofatto V 2012 The essential polysome-associated RNA-binding protein RBP42 targets mRNAs involved in *Trypanosoma brucei* energy metabolism. RNA (New York, N.Y.) 18, 1968–1983. (10.1261/rna.033829.112) PMC3479388 22966087

[291] Kramer S 2014 RNA in development: how ribonucleoprotein granules regulate the life cycles of pathogenic protozoa. Wiley Interdiscip. Rev. RNA 5, 263–284. (10.1002/wrna.1207) 24339376

[292] Protter DS, Parker R 2016 Principles and properties of stress granules. Trends Cell Biol. 26, 668–679. (10.1016/j.tcb.2016.05.004) 27289443 PMC4993645

[293] Lee MG-S 1995 Heat shock does not affect the transcriptional efficiency of the Hsp 70 genes of *Trypanosoma brucei*. Exp. Parasit. 81, 608–613. (10.1006/expr.1995.1156) 8543004

[294] Rocha AA, Moretti NS, Schenkman S 2014 Stress induces changes in the phosphorylation of *Trypanosoma cruzi* RNA polymerase II, affecting its association with chromatin and RNA processing. Eukaryot. Cell 13, 855–865. (10.1128/ec.00066-14) 24813189 PMC4135739

[295] Kramer S, Queiroz R, Ellis L, Webb H, Hoheisel J, Clayton C, Carrington M 2008 Heat shock causes a decrease in polysomes and appearance of stress granules in trypanosomes independently of eIF2*α* phosphorylation at threonine 169, the residue equivalent to serine 51 in mammalian eIF2*α*. J. Cell Sci. 121, 3002–3014. (10.1242/jcs.031823) 18713834 PMC2871294

[296] Minia I, Merce C, Terrao M, Clayton C 2016 Translation regulation and RNA granule formation after heat shock of procyclic form *Trypanosoma brucei*: many heat-induced mRNAs are increased during differentiation to mammalian-infective forms. PLoS Neglected Trop. Dis. 10, e0004982 (10.1371/journal.pntd.0004982) PMC5015846 27606618

[297] Kelly S, Kramer S, Schwede A, Maini P, Gull K, Carrington M 2012 Genome organization is a major component of gene expression control in response to stress and during the cell division cycle in trypanosomes. Open Biol. 2, 120033 (10.1098/rsob.120033) 22724062 PMC3376733

[298] Lee MGS 1998 The 3′ untranslated region of the Hsp 70 genes maintains the level of steady state mRNA in *Trypanosoma brucei* upon heat shock. Nucleic. Acids Res. 26, 4025–4033. (10.1093/nar/26.17.4025) 9705515 PMC147808

[299] Droll D, Minia I, Fadda A, Singh A, Stewart M, Queiroz R, Clayton C 2013 Post-transcriptional regulation of the trypanosome heat shock response by a zinc finger protein. PLoS Pathog. 9, e1003286 (10.1371/journal.ppat.1003286) 23592996 PMC3616968

[300] Singh A, Minia I, Droll D, Fadda A, Clayton C, Erben E 2014 Trypanosome MKT1 and the RNA-binding protein ZC3H11: interactions and potential roles in post-transcriptional regulatory networks. Nucleic Acids Res. 42, 4652–4668. (10.1093/nar/gkt1416) 24470144 PMC3985637

[301] Cassola A, De Gaudenzi J, Frasch A 2007 Recruitment of mRNAs to cytoplasmic ribonucleoprotein granules in trypanosomes. Mol. Microbiol. 65, 655–670. (10.1111/j.1365-2958.2007.05833.x) 17635187

[302] Holetz F et al. 2010 Protein and mRNA content of TcDHH1-containing mRNPs in *Trypanosoma cruzi*. FEBS J. 277, 3415–3426. (10.1111/j.1742-4658.2010.07747.x) 20629747

[303] Fritz M et al. 2015 Novel insights into RNP granules by employing the trypanosome's microtubule skeleton as a molecular sieve. Nucleic Acids Res. 43, 8013–8032. (10.1093/nar/gkv731) 26187993 PMC4652759

[304] Cristodero M, Schimanski B, Heller M, Roditi I 2014 Functional characterization of the trypanosome translational repressor SCD6. Biochem. J. 457, 57–67. (10.1042/bj20130747) 24087925

[305] Kruger T, Hofweber M, Kramer S 2013 SCD6 induces RNP granule formation in trypanosomes in a translation-independent manner, regulated by its LSM and RGG domains. Mol. Biol. Cell 24, 2098–2111. (10.1091/mbc.E13-01-0068) 23676662 PMC3694794

[306] Heyer EE, Moore MJ 2016 Redefining the translational status of 80S monosomes. Cell 164, 757–769. (10.1016/j.cell.2016.01.003) 26871635

[307] Lott K, Li J, Fisk JC, Wang H, Aletta JM, Qu J, Read LK 2013 Global proteomic analysis in trypanosomes reveals unique proteins and conserved cellular processes impacted by arginine methylation. J. Proteomics 91, 210–225. (10.1016/j.jprot.2013.07.010) 23872088 PMC3935770

[308] Iribarren PA, Berazategui MA, Bayona JC, Almeida IC, Cazzulo JJ, Alvarez VE 2015 Different proteomic strategies to identify genuine Small Ubiquitin-like MOdifier targets and their modification sites in *Trypanosoma brucei* procyclic forms. Cell. Microbiol. 17, 1413–1422. (10.1111/cmi.12467) 26096196

[309] Moretti NS, Cestari I, Anupama A, Stuart K, Schenkman S 2018 Comparative proteomic analysis of lysine acetylation in trypanosomes. J. Proteome Res. 17, 374–385. (10.1021/acs.jproteome.7b00603) 29168382

[310] Minia I, Clayton C 2016 Regulating a post-transcriptional regulator: protein phosphorylation, degradation and translational blockage in control of the trypanosome stress-response RNA-binding protein ZC3H11. PLoS Pathog. 12, e1005514 (10.1371/journal.ppat.1005514) 27002830 PMC4803223

[311] Lott K, Mukhopadhyay S, Li J, Wang J, Yao J, Sun Y, Qu J, Read LK 2015 Arginine methylation of DRBD18 differentially impacts its opposing effects on the trypanosome transcriptome. Nucleic Acids Res. 43, 5501–5523. (10.1093/nar/gkv428) 25940618 PMC4477658

