# Supplementary material for: Regulation of gene expression in trypanosomatids: living with polycistronic transcription
Source: Open Biol. 2019 Jun 5;9(6):190072. doi: 10.1098/rsob.190072 (PMC6597758; doi:10.1098/rsob.190072)
Supplement: Supplementary Notes [file rsob190072supp2.pdf]

Supplementary Notes

Introduction

Note 1: The full classification of the family Trypanosomatida is as follows: Phylum Euglenozoa; Class: Kinetoplastea; Subclass Metakinetoplastina; Order Trypanosomatida, [1]. The supergroups are Excavata, SAR (Stramenopila, Alveolata, Rhizaria), Archaeplastida, Opisthokonta and Amoebozoa; but some organisms do not fit into any of these. Animals and fungi are both in Opisthokonta, so are actually relatively closely related. The position of the root is controversial [2].

Note 2: Bloodstream forms are cultivated at 37°C in medium containing glucose, whereas procyclic forms are cultivated at 27°C in medium containing proline, and sometimes also glucose. The peptides that mediate quorum sensing mechanism in *T. brucei* are produced from host (or culture serum) proteins by secreted peptidases. For more details and references see [3].

Note 3: Sexual forms of *T. cruzi* and *L. major* have not been identified, but based on population genetics, they seem likely to exist (e.g. [4, 5]).

Genome organisation and transcription

Genome organisation

Note 4: Results from sequencing of a *Euglena* genome and transcriptome showed a similar overall genome architecture, with extensive *trans* splicing, but also a lot of *cis* splicing. Euglena is within Eugleozoa, but outside Metakinetoplastina. The loss of *cis* splicing probably occurred when the Kinetoplastea (which includes the trypanosomatids and their free-living relatives, the bodonids) branched from other Euglenids [6].

A recent claim for extensive alternative *cis*-splicing, and for miRNAs, in *Trypanosoma evansi* [7] is inconsistent with published results for another *T. evansi* isolate [8], and with the fact that *T. evansi* is a derivative of *T. brucei* that has defective mitochondrial DNA. Raw data for the *T. evansi* genome for which *cis* splicing was claimed are not publically available and the authors offered no supporting evidence. Moreover, the only example of *cis* splicing that they show in a Figure [7] shows translation of the antisense strand of the annotated open reading frame. Another paper containing miRNA predictions is also devoid of experimental evidence [9].

Note 5: For example, in *T. brucei*, fibre-FISH experiments demonstrated 19 copies of the alpha-beta-tubulin tandem repeat [10], and there are at least 40 different genes that are present in more than 3 copies per haploid genome [11].

Polymerase II transcription

Note 6: In yeasts, plants and mammalian cells, Maf1 is a general repressor of tRNA synthesis during stress or differentiation [12]. However it can also influence transcription by pol I and pol II, in part by specific regulation of TBP transcription [12]. *T. brucei* has a MAF1 homologue and its depletion increased most transcription (relative to 18S) while over-expression decreased it [13]. In both cases parasite growth was inhibited, suggesting that the relationship between transcription and growth is finely tuned. The effect of MAF1 seemed to be more general than in other organisms, since it was enriched on tRNA, *SLRNA*, 5S rRNA and procyclin promoters [13].

Note 7: In *Leishmania major*, episomes that contain only plasmid backbone, RNA processing signals and a selectable marker gene are transcribed [14]. In *T. brucei*, polymerase II initiation can be turned on by transcription by a bacteriophage polymerase with a cognate integrated promoter [15], or by inserting a reporter in a region that is kept open by RNA polymerase III activity [16].

A ~200bp GT-rich sequence was able to drive 3-5x more forward transcription of a reporter than an inverted version`, if integrated at the centre of a strand-switch region [17]. Curiously, early ChIP results for

TRF4 and the largest RNA polymerase II subunit suggested that both accumulate at the ends of some coding regions [18]. ChIP-Seq results for the RNA polymerase II subunit RPB9 [17] do not substantiate this, however.

Polymerase II termination

Note 8: SnoRNA precursors are synthesised by RNA polymerase II, and are *trans* spliced and polyadenylated before cleavage to the final products. Whether they are independently transcribed, or made as part of polycistronic units, is unknown (references in [19]). The distribution of the RNA polymerase II subunit RPB9 over snoRNA clusters is rather more irregular than over regions that are processed to make mRNAs (see tritrypdb.org for data from [17]).

Note 9: RBP33 is essential in both bloodstream and procyclic form *T. brucei*. In addition to associating with various mature mRNAs, it binds to snoRNAs, tRNAs, and to very low abundance mRNAs, and affects the abundances and lengths of retroposon and repeat RNAs [20]. Overexpression of RBP33 caused cell cycle arrest, with decreases in total mRNA and *SLRNA* [21]. This result is difficult to interpret since over-expressed RNA-binding proteins may bind to sequences that are not normally bound when the protein is expressed at the normal level; and decreases in mRNA and *SLRNA* levels could be secondary to transcription arrest. The result nevertheless shows that RBP33 dosage is very important.

mRNA processing and export

The 5' cap and *SLRNA* processing

Note 10: The start sites for polymerase II transcription of protein-coding genes were identified through their possession of a 5'-triphosphate. It therefore seems unlikely that these primary transcripts are capped; ChIP data for capping enzymes, which might resolve this, are not available.

Splicing and polyadenylation: basal machineries and signals

Note 11: In 1982, the spliced leader on *VSG* mRNA was thought to be added by *cis*-splicing [22] but evidence for *trans* splicing was strong by 1984 [23-25]. *Trans*-splicing was found in *C. elegans* later, in 1987 [26]. Apart from its presence throughout *Euglenozoa*, *trans* splicing has since been observed in other animals, including *Cnidaria, Ctenophora, Rotifera, Platyheminthes, Tunicata, Arthropoda*; in *Perkinsozoa* and dinoflagellates [27], and most recently in a Cryptomonad [28] and the rhizarian *Paulinella micropora* [29]. Is this the result of multiple evolutionary accidents? There is no obvious selective advantage.

Note 12: Results in *T. brucei* using transiently transfected reporters [30] suggested that $(U)_{10}$ was sufficient to obtain a functional mRNA, while maximal activity was attained with 25nt, but in this study the mRNAs themselves were not analysed. So far there is little evidence for effects of polypyrimidine tracts length on mRNA abundance [31], but extremely little relevant work has been done. RNA-Seq results [32, 33] confirmed that *T. brucei* trans splicing sites are downstream of polypyrimidine tracts (median length 18nt) with a strong preference for U over C; in *Leishmania* this preference is not so pronounced [34]. Several alternative 3' acceptor AG dinucleotides downstream of the polypyrimidine tract may be used, but there is a preference for the first one [35]; the median distance from the tract to the AG in *T. brucei* is 43nt [32] but distances range to 200nt. Mapping of branch points for eighteen *T. brucei* genes [36, 37] and one from *T. cruzi* [35] suggested use of the nearest A residues upstream of the polypyrimidine tract, with no upstream consensus sequence - suggesting that pairing of the branch point with the U2 snRNA does not occur.

The mRNAs at the beginnings and ends of polycistronic transcripts are processed in just the same way: polyadenylation occurs upstream of first splice site, either at divergences or downstream of tRNA genes. This results in uncapped but polyadenylated RNAs which are presumably rapidly destroyed. At the end of the unit, the last remnant before termination is spliced but not polyadenylated [32].

Note 13: Transcriptome-wide data for *T. brucei* and *Leishmania*, and cDNA studies for *T. cruzi*, indicated that the approximate distances between the polypyrimidine tract and polyadenylation sites are 100nt for *T. brucei* [32], 400nt for *Leishmania* [38], and around 50 nt) in *T. cruzi* [39]. Cleavage and polyadenylation

factor CPSF3 (or CPSF73) co-purified with the U1 snRNP protein U1A [40] - but the involvement of U1A in *cis* splicing is unclear.

Note 14: Site-directed mutagenesis of *CPSF3* also gave moderate resistance to acoziborole [41]. Notably, a different benzoxaborole series targets the same process in *Plasmodium*, and in that case, 10000-fold resistance could easily be obtained. The failure to generate any trypanosome resistance above about 5-fold (see also [42]), combined with the absence of structure-function relationships with different benzoxaboroles [43], suggests that one or more additional targets may be present, although it also possible that resistance mutations in *T. brucei* CSPF3 are incompatible with activity. Inhibition of CPSF3 enzyme activity by these compounds has not yet been reported.

Note 15: In Opisthokonts and plants,splicing factors interact with the phosphorylated C-terminal domain [44-46]. Alternative splicing patterns are influenced by chromatin status and the rate of polymerase II elongation [47], and elongation is in turn influenced by splicing [48, 49].

Note 16: At present it isn't possible to say whether the trypanosomatid splicing machinery is influenced by chromatin or not. In *T. brucei*, many polyadenylation sites and most splice signals contain poly(A:T) tracts, and nucleosomes are depleted over each, with a strong nucleosome positioning immediately downstream (and therefore at splice acceptor sites) [50]. It is not known whether this nucleosome alignment is important for mRNA processing. RNA-DNA hybrids were also much more concentrated in intergenic regions than coding regions [51]; the significance of this is unknown. In *Leishmania*, although there is there is some nucleosome depletion at poly(A) sites, the pattern for splice sites is opposite to that in trypanosomes, with strong nucleosome positioning immediately *upstream* of the splice site, with depletion at the site itself [52]; this may be because, in contrast to *T. brucei*, *Leishmania* polypyrimidine tracts contain roughly equal levels of C and T [38].

Note 17: U2AF35, U2AF65, and SF1 depletion affected both splicing (at a global level) and stability [53]. The effects of depleting these factors on mature mRNA abundances could not be correlated directly with the effects on splicing. mRNAs that were decreased after U2AF65 or SF1 depletion had very slightly shorter than average polypyrimidine tracts, while those that increased had more purine-rich exons [53].

Quality control of RNA processing

Note 18: To test the influence of length, we measured the abundances of mRNAs from two loci that included very long open reading frames. We progressively truncated each through integration of a reporter at different positions. Although the shorter versions were indeed more abundant, increased mRNA stability accounted for the differences [54]. The conclusions concerning both length and the effects of differing processing relied on modelling, using half-lives that were measured after inhibition of transcription with Actinomycin D, and of splicing with the general methylation inhibitor Sinefungin. It is likely that this method gives incorrect half-lives for at least some mRNAs (see for example [55]).

Note 19: The principal role of the exosome is processing of stable RNAs in the nucleus, but a fraction is also present in the cytosol. It has a ring-like core that consists of 6 proteins, with a central channel through which RNAs can pass [56]. At the top (entrance), there are three S1 domain proteins. These 9 proteins together act to unwind RNAs, making them more accessible to exonucleases. Substrates that transit the channel are degraded a different, processive exoribonuclease called Rrp44 (all names here from *S. cerevisiae*), the active site of which is near the channel exit [57, 58]. Rrp44 also, however, has an additional endonuclease domain (PIN domain) that is not oriented towards the channel. A distributive 3'-5' exoribonuclease called Rrp6 (in *S. cerevisiae*) binds towards the top (entrance) of the exosome. Rrp6 is responsible for trimming rRNAs; its substrates are recruited by the exosome (and cofactors) but do not travel throught the entire central channel [59]. In yeast, Rrp6 is predominantly in the nucleus whereas Rrp44 is mostly in the cytoplasm, whereas in human cells, Rrp6 is also cytoplasmic.

Note 20: Trypanosomes have the core exosome, and homologues of both Rrp6 (RRP6) and Rrp44 (RRP44) [60, 61]. The proportions of exosome proteins found in nucleus and cytoplasm vary depending on methods used; results indicate that it is mostly, but not exclusively, in the nucleus [62, 63]. RRP6 is reproducibly exosome-associated, and found in both cytoplasm and nucleus [62]. RRP44 does not co-purify with the exosome core on affinity columns, and does not co-sediment with the complex on a glycerol

gradient [60]. Despite this, depletion of RRP44 - like depletion of demonstrated exosome subunits - results in impaired rRNA processing [60, 64], suggesting that it is indeed associated with the exosome *in vivo*. Proximity labelling methods might resolve this issue.

Note 21: The *T. brucei* alpha and beta tubulin genes are arranged in an alternating tandem repeat [65, 66]. When splicing is impaired, beta-alpha dimer and tetramer tubulin mRNAs accumulate [67, 68], suggesting less efficient alpha-tubulin splicing [69]. This could be because the alpha-tubulin polypyrimidine tract is shorter, and less U-rich than that for beta-tubulin.

Note 22: The effect of exosome depletion on mRNA was so far seen for three different loci containing repeated genes, including alpha-beta tubulin. It could relatively easily be examined transcriptome-wide by re-analysis of existing RNASeq datasets from exosome-depleted cells [70].

Splicing and polyadenylation: possible specific regulators

Note 23: The proteins that interacted were Tb927.10.15870, DRBD2, and RBP23. TSR1IP, but not TSR1, also showed some co-migration with monosomes or disomes in a sucrose gradient but not clear if this was actual association.

Note 24: These proteins have at least two RRM domains, but possibly more. The number of motifs identified in a protein depends on the algorithm used and the threshold that is set. The C-rich DRBD3 recognition sequence is present in the *PAP1* intron and DRBD3 depletion impaired *PAP1 cis*-splicing [71].

Note 25: RHS2 proteins may also exit the nucleus with mRNAs as they have been implicated in both mRNA export and translation [72].

Export of mRNAs to the cytoplasm

Note 26: The additional proteins are CBP110, CBP66 and CBP30 [73]. CBP66 has a zinc finger so might bind - perhaps specifically - to the *SL* sequence.

Note 27: These proteins are essential in mammals, and are conserved in all eukaryotes except those with very low intron densities [74].

Note 28: *T. brucei* EIF4AIII was not detectably associated with Y14 [75], and it is enriched in the nucleolus [76], consistent with the role of the yeast homologue, FAL1, in rRNA processing. Results using a different antibody, in both *T. cruzi* and *T. brucei*, gave a different location, on the cytosolic side of the nuclear pore [77]. These authors presented evidence that the protein shuttles between nucleus and cytoplasm, with export depending on interaction with MEX67 [77]. An NTF2 (nuclear transport factor) domain protein that was associated with Y14-Magoh [75] is predominantly cytosolic.

Translation

Cap-binding translation initiation complexes

Note 29: The efficiency of *in vitro* translation initiation is enhanced by the presence of a poly(A) tail on the mRNA. All cytosolic mRNAs that have been examined so far were strongly compacted (relative to the stretched-out length), but interaction between the 5' and 3' ends is seen in only a minority [78-80], suggesting that circularization might be non-obligatory, or occur transiently during initiation.

Translation initiation

Note 30: The "tethering" assay involves expression of a protein fused to a (poly)peptide that binds to a target RNA sequence with high affinity and specificity, together with a reporter RNA that bears that sequence [81]. One example is the lambdaN peptide, which binds to the boxB sequence. The assay measures how a protein affects the mRNA to which it is bound, so can give information about the protein's possible effect on mRNA decay and translation without any prior knowledge of the mRNAs to which that protein binds. The assay can give artifactual answers - for example, if fusion with the RNA-binding peptide affects activity, or the protein in question never, under normal circumstances, binds to RNA. Additional evidence is therefore always required. In high-throughput assays using random-shotgun fragments, false-

positive and false-negative results can occur, since fragments may lack interactions or be prone to aggregation [82]. Results with full-length proteins [83] are more reliable but artifacts still cannot be ruled out.

Note 31: It has been reported that the internal ribosome entry site of Cricket Paralysis Virus functions in *T. brucei*; those of Encephalomyocarditis Virus and Human Papilloma Virus do not [84]. If confirmed, this could be a useful experimental tool.

Note 32: EIF4E1 might be similar to the cap-binding mammalian protein 4EHP and its interaction partner GIGYF2 [85]. 4EHP has no initiation activity by itself, and tethered GIGYF2 inhibits independently of 4EHP [85] - as does 4EIP [86]. Alternatively, the model may be more similar to the action of 4E-BPs on their target eIF4Es - in which case 4EIP would inhibit active initiation by EIF4E1. To test this it is necessary to know which proteins are bound by EIF4E1 in the absence of 4EIP. An interaction between Leishmania EIF4E1 and eIF3 was seen using proteins made *in vitro* [87], but has not been confirmed i*n vivo*.

Scanning, elongation and termination

Note 33: Oddly, even mRNAs without uORFs from procyclic forms were found to have substantial ribosome occupancy of the 5'-UTR [88]. This be a consequence of slow initiation (perhaps "queuing" 40S subunits were also included) but might also have been influenced by cycloheximide treatment.

Note 34: The median half-lives of mRNAs with uORFs are 11.3 min for bloodstream forms, and 16.5 min for procyclic forms; for remaining mRNAs the values are 11.8 min and 19.7 min (calculated from [89]).

Note 35: In mammals, a protein called Upf3 binds to the exon junction complex, while Upf1 is recruited by the translation termination complex, which includes proteins called eRF1 and eRF3. Upf1 recruits Upf2. If termination occurs upstream of an exon junction, Upf1 is activated by a kinase and the complex recruits an endonuclease which cleaves the mRNA [90-92]. In *S. cerevisae*, which lacks the exon-junction complex and has very little splicing, Upf1-dependent nonsense-mediated decay seems to be triggered in mRNAs that have abnormally long 3'-UTRs, or perhaps in mRNAs with G-or GC-rich sequences near the termination codon, and these may influence NMD in other species as well [92-95].

In *T. brucei*, insertion of a premature termination codon in either a reporter or an endogenous gene decreased mRNA abundance, and tethering of UPF1 to a reporter also suppressed expression [96]. These results would be consistent with NMD. Unfortunately, depletion of UPF1 by RNAi was not very effective, so the lack of any effect on the mRNA abundances, for either reporters of the whole transcriptome, could not be interpreted. Nonsense-mediated decay should also be dependent on ongoing translation, but insertion of a translation-inhibitory hairpin did not increase the abundance of a reporter with a premature termination codon. Instead, the hairpin decreased the abundance of mRNAs without premature termination codons, in tune with the general positive correlation between translation and mRNA stability [96].

mRNA decay

Deadenylation

Note 36: Other complex components that are conserved from trypanosomes to man are NOT2, NOT3/5, NOT10, NOT11 and the 3'-5' A-specific processive exonuclease CAF1. Opisthokonts also have NOT9, and in most species examined so far, an additional deadenylase enzyme, CCR4, is attached to the complex via CAF1. The helicase DHH1 is often co-purified but may be associated indirectly. The functions of the other subunits are not really understood in any organism. A homologue of NOT9 exists in trypaosoes, but no interaction with the trypanosome complex was detected. Trypanosomes do have a protein similar to CCR4, but it lacks the CAF1-interacting domain and, like a possible NOT9 homologue, shows no detectable association with the NOT complex [97]. Trypanosome NOT10 is required to stabilise the association of CAF1 with NOT1, but this is not the case in human cells [98], and in yeast, NOT10 may be replaced by a different protein, Caf130.

Note 37: Metabolic labelling with 4-thio-uridine or 4-thio-uracil, followed by a chase period, it the most popular way to determine mRNA half-lives since it results in minimal disturbance of cellular function [55].

Unfortunately, incorporation of 4-thoi-uridine into mRNA by trypanosomes is exceptionally inefficient. All measurements of mRNA half-lives to date have involved inhibition of RNA synthesis (with Actinomycin D) and/or of trans splicing (with Sinefungin). This method is unsuitable for determining mRNA half-lives longer than 4-6h, because of toxicity. Moreover, it is susceptible to artifacts. Notably, the amounts of many mRNAs appear to increase (relative to total RNA) before they decrease. This seems impossible - and has never been satisfactorily explained. Even if both mRNA synthesis and processing are inhibited (using Actinomycin D and Sinefungin, respectively), this phenomenon persists. Actinomycin D treatment also leads to accumulation of poly(A)-containing RNA in the nucleus, suggesting an effect on export [72].

Transcriptome-wide measurements in trypanosomes using both Actinomycin D and Sinefungin suggested three different decay patterns: immediate exponential decay, decay after a delay (perhaps indicating gradual deadenylation), and very rapid decay followed by more gradual loss [89]. The last pattern could be explained if a fraction of the mRNA is immediately decapped, while the remainder is first subject to deadenylation [99-101]. Using inhibitors, complex decay patterns were also seen in yeast [102], but more recent pulse-chase assays did not reproduce this and yielded substantially shorter half-lives [55]. To measure mRNA decay in future, it should be possible to label mRNAs with 5-ethynyl uridine, chase, and then sequence labeled RNAs, as described in [72].

Decapping and degradation of the body of the mRNA

Note 38: This enzyme, which the authors unfortunately named Dcp2, is clearly not the major decapping enzyme since it has poor activity on the complete modified cap; moreover its depletion has no effect on trypanosome multiplication [100, 103, 104], whereas all other components of the constitutive pathway are essential.

Note 39: Intriguingly, XRNA depletion led to accumulation of an aberrantly spliced version of the EP procyclin mRNA [100]. This is however a single observation which remains to be confirmed, and no such effect was seen for a few other mRNAs [63, 105]. Re-examination of transcriptome data could reveal whether or not mRNAs with alternative 5'-UTRs accumulate after XRNA depletion.

Regulation of mRNA decay and translation

The role of untranslated regions

Note 40: The published estimates of 300-500nt [32, 33, 106] are probably under-estimates since potential polyadenylation sites that were many kb from the end of the ORF were generally not considered during the annotation. The estimate of the number of possible bound proteins is a lower estimate, since it is based on the 30nt occupancy of PABP. PABP has 4 RRM domains, but many kinetoplastid RBPs have only a single RNA recognition motif (RRM) or CCCH ($CX_8CX_5CX_3H$) domain, so could recognise only 3-5 bases [107-109]. Pumilio (Puf) domain proteins usually recognise 7-8 bases [110-112].

Environmental adaptation and differentiation

Note 41: Diverse growth-inhibitory *in vitro* treatments of long slender *T. brucei* result in at least partially differentiation-competent stumpy-like cells, with varying subsets of the expected mRNA changes [3, 113-117], but so far no *in vitro* stumpy-formation protocol has been able to mimic fully the process that is seen inside a mouse [118]. Growth-inhibitory treatments of procyclic forms can also cause a partial switch towards bloodstream-form gene expression (e.g. [119]), but it is difficult to tell whether such responses are biologically meaningful, unless the cells are then able to grow as bloodstream forms.

Note 42: The prozyme and enzyme subunits of S-adenosylmethionine decarboxylase are both required for enzyme activity. Repression of prozyme mRNA translation by high levels of decarboxoylated S-adenosylmethionine depends on the presence of the enzyme subunit [120]. A catalytically inactive version of the enzyme suffices for repression but here is currently no evidence for a direct enzyme-mRNA interaction [82]. A microarray analysis documented increases or decreases in expression of 138 mRNAs in response to addition of guanosine to late log-phase procyclic forms. Repression of expression of the NT8

purine transporter mRNA by guanosine or hypoxanthine, or in early log phase, required the presence of a 60nt putative stem-loop in the 3'-UTR [121].

Regulation by RNA-binding proteins

Note 43: The mRNP includes cap-binding proteins, PABPs, various other RBPs bound to the mRNA, and other proteins that interact with them. Proteins that bind to the 5'-UTR or coding region (including the EJC) will be swept away by ribosomes, whereas those on the 3'-UTR may persist through numerous rounds of translation. Associations depend on the binding affinities and intracellular concentrations of RBPs, and the number of available target sequences. The behaviour of an mRNA will depend on the combined effects of all bound proteins, which will in turn be affected by the age and poly(A) tail length of the mRNA. Changes in binding of just one protein may well not suffice to change RNA fate. Unfortunately, it has so far not been possible to isolate individual mRNAs in sufficient purity and yield to identify the specifically-bound RBPs. Instead, we have to rely on incomplete information from studies of individual RBPs. A Puf domain binds a single base, but the presence of 7-8 Puf domains ensures RNA specificity [110]. RRM and CCCH domains recognise only 3-5 bases [107-109]. Consequently, in order to have specificity for particular mRNAs, at least two RRM or CCCH domains must be present in a protein or protein complex.

Note 44: To find all proteins that were bound to mRNAs in bloodstream forms, cells were UV-treated to create protein-RNA cross-links. Proteins were then identified after purification of poly(A)+ mRNA under very stringent conditions [83] (supplementary TAble S1). Several of the bound proteins - including some which are metabolic enzymes - lacked known RNA binding domains, but the functions of these in gene expression are not yet known. Results from tethering [82, 83] are incomplete since in some cases, only protein fragments were assayed.

Cell-cycle regulation

Note 45: Data for RNA binding and tethering are from *T. brucei*. *T. cruzi* ZC3H39 is associated with ZC3H40, and with an RNA helicase, a possible translation elongation factor, release factor ERF1, and a few other proteins. It is found in mRNP granules in stressed epimastigotes [122]. A potential binding sequence, AAACAA, was suggested for the 3'-UTRs of target mRNAs, but the complete list of RNA targets was not supplied, enrichment of the motif relative to unbound RNAs was not assessed, and the role of the sequence in ZC3H39 binding to mRNAs was not tested [122].

Procyclic-form-specific mRNAs

Note 46: RBP10 has poly(Q) regions which might mediate protein-protein interactions or aggregation. A yeast 2-hybrid screen yielded too many potential binding partners to be interpretable. This could be because RBP10 is prone to aggregation, as seen after over-expression of the protein in procyclic forms [123]. Interactions of RBP10 were also investigated using proximity biotinylation (BioID), after expression of a BirA fusion in procyclic forms [123]. More than 180 proteins were identified as putative interactors, including several implicated in mRNA pathways [123]. Comparison of the BioID results with those from RBP10 affinity purification [124] yielded a few common interaction partners, including CAF40 and some proteasome subunits, but the two-hybrid results did not overlap.

Note 47: Expression of the bloodstream-form-specific repressor RBP9 in procyclic forms prompted a switch towards a bloodstream-type transcriptome [123], but our preliminary results (unpublished) for RNA binding and RNAi in bloodstream forms do not support a role in developmental regulation. Pull-down of *T. cruzi* RBP9 from exponentially growing epimastigotes revealed association with numerous RNA-binding proteins, both PABPs, and EIF4G5 [125].

Note 48: Bloodstream forms lacking ZFP1 were capable of surface protein exchange during differentiation, but could not reposition the kinetoplast towards the nucleus [126]. RNAi targeting ZFP2 reduced procyclin expression, morphological changes, and subsequent growth in differentiating monomorphic cells; over-expression also resulted in abnormal morphology [127]. It would be interesting to re-examine ZFP1 and ZFP2 function in pleomorphic parasites. Over-expression of ZFP3 in bloodstream forms accelerated EP procyclin expression during differentiation [128]. In procyclic forms, over-expression resulted in elongation

of the posterior part of the parasites [128]. RNA binding by ZFP3 was examined, first using a candidate approach and later with a microarray. ZFP3 indeed associates with the EP procyclin 3'-UTR [128], and this requires both the UA$(U)_6$-containing regulatory 26mer and a 16mer that promotes translation [129]. Neither RNAi nor over-expression affected the of ZFP3 abundances of EP and procyclin mRNAs, but at the protein level, an increase in EP at the expense of GPEET was seen [129]. This could well be indicative of a switch from early to late procyclic forms. A microarray analysis, however, showed that ZFP3 also associates with many other mRNAs that are not regulated during any analysed transition [130].

Note 49: The "early" phenotype can be maintained by glucose deprivation or addition of glycerol to the medium and is prevented by hypoxia, by RNAi that inhibits pyruvate catabolism, and by inhibiting the mitochondrial alternative oxidase [131, 132]. Conversely, GPEET expression was promoted by alternative oxidase over-expression [133]. A sequence that overlaps with the 26mer of *GPEET* mRNA specifies this regulation [131, 132]. It is not known what this element responds to - a metabolite, or perhaps levels of oxidative stress? The role of the cytosolic terminal uridylyl transferase TUT3 is not known. The only other candidate, TUT4, is cytosolic by cell fractionation (R. Aphasizhev, Boston University, personal communication) but a GFP fusion localises to mitochondria [134].

From procyclic to metacyclic forms.

Note 50: In cultured bloodstream and procyclic forms, there is approximately one *RBP6* mRNA for every 2 cells [89]. Although recombinant RBP6 interacts with repeats of AUUU but not AUCU [135], these were the only sequences tested and its RNA targets *in vivo* are not known.

Note 51: Possible epimastigote- or metacyclic-specific RNA-binding proteins are DRBD6, PUF5, PUF11, and SLBP1 and, in some datasets, RBP7. The fact that induced expression of PUF5 in procyclic forms inhibited growth [136] could indicate a role in differentiation.

Note 52: Ectopic expression of myc-tagged DRBD13 slightly increased levels of three bloodstream-form specific mRNAs including *VSG* [137]. If DRBD13 represses RBP6 expression the opposite would be expected. However changes in mRNA levels were all less than 1.5-fold.

Abundant mRNA-binding RBPs with unclear functions.

Note 53: In bloodstream form *T. brucei*, UBP1 and UBP2 appear to bind to and stabilise the mRNAs encoding the cyclin-F-box-containing proteins CFB1 and CFB2. UBP2 also accentuates developmental regulation of pyruvate transporter mRNAs [138]. Studies with RNA homopolymers indicated that *T. cruzi* UBP1, UBP2, and the single-RRM RBPs 2-6 had differing sequence preferences [139], and a limited survey of mRNAs bound to UBP1 and RBP3 [140] also suggested some targets. However, comprehensive studies are missing for these proteins, and screening results relied on relatively insensitive microarrays. While *T. cruzi* RBP3 bound to ribosomal protein mRNAs [140], ten completely different bound targets were found for the *T. brucei* version [141]. A microarray after *RBP3* RNAi did not reveal any changes in gene expression [141].

Note 54: The *AATP11* mRNA has two RBP10 binding sites; deletion analyses suggest that these may contribute to, but are not sufficient for, down-regulation in bloodstream forms [142]. The same RNA also binds to DRBD3 in procyclic forms, and is destabilised by DRBD3 depletion. The DRBD3 binding sites are towards the 3'-half of the 3'-UTR [143], which appeared, from deletion analyses, to be needed for a high procyclic-form mRNA level [142].

Stress responses and ZC3H11

Note 55: Stress granules are thought to form by a phase-separation mechanism which is assisted by poly-Q tracts and disordered regions in some of the component proteins [144, 145]. Aggregation can be inhibited by arginine methylation [146]. SCD6, a universal granule component, suppresses translation in Opisthokonts, preventing formation of 48S complexes [147] and binding eIF4Gs via RGG motifs [148]. Methylation of those motifs enhances the repression [149].

Note 56: The fact that translation inhibition stabilizes mRNAs has often been interpreted to mean that the degradation depends on an unstable protein factor. Although this may well, in some cases, be true,

evidence from translation inhibitors alone alone cannot be used to draw the conclusion. Cycloheximide causes ribosomes to build up on the mRNA; puromycin results in the formation of RNA-protein granules [150] and may induce stress because of a build-up of protein fragments [119]. One publication showed that stopping translation of the reporter under investigation, by adding a stem-loop in the 5'-UTR, did not have the same effect as inhibitor treatment [151], but even there, a stress response is not ruled out. Even if an unstable protein is involved in mRNA decay, it could just be one of the decay enzymes - for example, the stabilities of ALPH1 and XRNA are not known. Interactions between termination factors or initiation factors with the decay machinery are also likely.

Note 57: The five *T. brucei* genes encoding the major cytosolic HSP70 [152] are present as a tandem repeat at least 74kb from the transcription start site. (It may be further since the genome assembly collapses tandem repeats.) Very severe heat shock also inhibits mRNA processing [153]. The authors thought that processing of *HSP70* mRNA continued at high temperature [154] because the precursor:mature ratio for tubulin mRNAs increased, whereas the same ratio for *HSP70* mRNA did not. This is however probably because tubulin mRNAs are degraded, while *HSP70* mRNAs have increased stability.

Chromatin immunoprecipitation results showed that a 41°C heat shock caused a 1.5-3-fold increase in the pull-down of several different DNA segments with histone H3. This may suggest that the loss of transcription is caused by - or results in - chromatin compaction. Exceptionally, association of histone H3 with the *HSP70* genes was 12-fold increased. The duration of the heat shock is not given, but if it was (as for the other experiments) 1 h, the increased pull-down of *HSP70* is at odds with the idea that their transcription continues because they are a long way away from the start site [155]. On the other hand, nuclear run-on results suggested strong inhibition of *HSP70* gene transcription within 45min at 41°C [156]. GRO-Seq labeling experiments [72] could be used to address these contradictory results.

Note 58: RNAi targeting *MKT1* was lethal in bloodstream forms but did not affect growth or survival of procyclic forms after heat shock. Although it is possible that ZC3H11 acts on chaperone mRNAs without needing MKT1 in procyclic forms, it is more likely that the low levels of MKT1 that remain after RNAi are sufficient. Attempts to delete both *MKT1* genes or both *ZC3H11* genes in procyclic forms failed (our unpublished results and [157]).

Note 59: Upon over-expression, *T. brucei* SCD6 aggregates via the LSm domain, and the RGG domain promotes formation of larger granules [158].

The odd behaviour of mRNAs encoding ribosomal proteins

Note 60: Median values are as follows:

	Bloodstream ribosomes/kb	Procyclic ribosomes/kb	Bloodstream half-life, min	Procyclic half-life, min
ribosomal protein RNAs	2.3	2.0	>120	>240
All other RNAs	2.7	6.5	12	19.7
Other with RNAs with t1/2 >30 min	4.3	14.0		

Only one copy each of repeated or similar genes was considered. Data are from [89, 159].

The discrepancy is particularly obvious in procyclic forms, which also show a much stronger relationship between ribosome occupancy and mRNA half-life than bloodstream forms.

Note 61: Ribosomal protein mRNAs were depleted in pull-downs of UBP1 and PUF2 [160], ZC3H32 [161], ZC3H30 [162], TRRM1 [163] and DRBD13 [137], but all of these datasets showed bias towards long RNAs in the bound fractions.

Regulating the regulators

Note 62: PMRT1 and consists of catalytically active and proenzyme subunits [164]. Proteins that are potentially associated with PRMT1 include DRBD13, TRRM1, RBSR1, SCD6 and ALPH1 [165]. However

only a few proteins altered their association with mRNA in the absence of PMRT1, the most notable effects being decreased association of EIF4G5, G5IP, PUF11, RBP35 and DRBD17.

Literature cited

1 Cavalier-Smith, T. 2016 Higher classification and phylogeny of Euglenozoa. *Eur J Protistol*. **56**, 250-276. (10.1016/j.ejop.2016.09.003)

2 Burki, F. 2014 The eukaryotic tree of life from a global phylogenomic perspective. *Cold Spring Harb Perspect Biol*. **6**, a016147. (10.1101/cshperspect.a016147)

3 Rojas, F., Silvester, E., Young, J., Milne, R., Tettey, M., Houston, D. R., Walkinshaw, M. D., Perez-Pi, I., Auer, M., Denton, H., *et al.* 2018 Oligopeptide signaling through TbGPR89 drives trypanosome quorum sensing. *Cell*. (10.1016/j.cell.2018.10.041)

4 Rougeron, V., De Meeus, T., Banuls, A. L. 2015 A primer for Leishmania population genetic studies. *Trends Parasitol*. **31**, 52-59. (10.1016/j.pt.2014.12.001)

5 Baptista Rde, P., D'Avila, D. A., Segatto, M., do Valle, I. F., Franco, G. R., Valadares, H. M., Gontijo, E. D., Galvao, L. M., Pena, S. D., Chiari, E., *et al.* 2014 Evidence of substantial recombination among Trypanosoma cruzi II strains from Minas Gerais. *Infect Genet Evol*. **22**, 183-191. (10.1016/j.meegid.2013.11.021)

6 Field, M. e. a. 2017 Unlocking the biological potential of *Euglena gracilis*: evolution, cell biology and significance to parasitism. *bioRxiv*. 228015. (10.1101/228015)

7 Zheng, L., Jiang, N., Sang, X., Zhang, N., Zhang, K., Chen, H., Yang, N., Feng, Y., Chen, R., Suo, X., *et al.* 2019 In-depth analysis of the genome of *Trypanosoma evansi*, an etiologic agent of surra. *Sci China Life Sci*. **62**, https://doi.org/10.1007/s11427-11018-19473-11428. (10.1007/s11427-018-9473-8)

8 Carnes, J., Anupama, A., Balmer, O., Jackson, A., Lewis, M., Brown, R., Cestari, I., Desquesnes, M., Gendrin, C., Hertz-Fowler, C., *et al.* 2015 Genome and Phylogenetic Analyses of Trypanosoma evansi Reveal Extensive Similarity to T. brucei and Multiple Independent Origins for Dyskinetoplasty. *PLoS neglected tropical diseases*. **9**, e3404. (10.1371/journal.pntd.0003404)

9 Mallick, B., Ghosh, Z., Chakrabarti, J. 2008 MicroRNA switches in *Trypanosoma brucei*. *Biochemical and biophysical research communications*. **372**, 459-463. (10.1016/j.bbrc.2008.05.084)

10 Ersfeld, K., Asbeck, K., Gull, K. 1998 Direct visualisation of individual gene organisation in Trypanosoma brucei by high-resolution in situ hybridisation. *Chromosoma*. **107**, 237-240.

11 Manful, T., Fadda, A., Clayton, C. 2011 The role of the 5'-3' exoribonuclease XRNA in transcriptome-wide mRNA degradation. *RNA (New York, N.Y.)*. **17**, 2039-2047. (10.1261/rna.2837311)

12 Zhang, S., Li, X., Wang, H. Y., Steven Zheng, X. F. 2018 Beyond regulation of pol III: Role of MAF1 in growth, metabolism, aging and cancer. *Biochim Biophys Acta Gene Regul Mech*. **1861**, 338-343. (10.1016/j.bbagrm.2018.01.019)

13 Romero-Meza, G., Velez-Ramirez, D. E., Florencio-Martinez, L. E., Roman-Carraro, F. C., Manning-Cela, R., Hernandez-Rivas, R., Martinez-Calvillo, S. 2016 Maf1 is a negative regulator of transcription in *Trypanosoma brucei*. *Molecular microbiology*. (10.1111/mmi.13568)

14 LeBowitz, J. H., Coburn, C. M., McMahon-Pratt, D., Beverley, S. M. 1990 Development of a stable Leishmania expression vector and application to the study of parasite surface antigen genes. *Proceedings of the National Academy of Sciences of the United States of America*. **87**, 9736-9740.

15 McAndrew, M., Graham, S., Hartmann, C., Clayton, C. E. 1998 Testing promoter activity in the trypanosome genome: isolation of a metacyclic-type VSG promoter, and unexpected insights into RNA polymerase II transcription. *Exp. Parasitol.* **90**, 65-76.

16 Marchetti, M. A., Tschudi, C., Silva, E., Ullu, E. 1998 Physical and transcriptional analysis of the *Trypanosoma brucei* genome reveals a typical eukaryotic arrangement with close interspersion of RNA polymerase II- and III-transcribed genes. *Nucleic acids research*. **26**, 3591-3598.

17 Wedel, C., Förstner, K., Derr, R., Siegel, N. 2017 GT-rich promoters can drive RNA pol II transcription and deposition of H2A.Z in African trypanosomes. *EMBO J*. **36**, 2581-2594. (10.15252/embj.201695323)

18 Ruan, J. P., Arhin, G. K., Ullu, E., Tschudi, C. 2004 Functional characterization of a Trypanosoma brucei TATA-binding protein-related factor points to a universal regulator of transcription in trypanosomes. *Mol Cell Biol*. **24**, 9610-9618. (10.1128/mcb.24.21.9610-9618.2004)

19 Chikne, V., Gupta, S. K., Doniger, T., K, S. R., Cohen-Chalamish, S., Ben-Asher, H. W., Kolet, L., Yahia, N. H., Unger, R., Ullu, E.*, et al.* 2017 The Canonical Poly (A) Polymerase PAP1 Polyadenylates Non-Coding RNAs and Is Essential for snoRNA Biogenesis in Trypanosoma brucei. *J Mol Biol*. (10.1016/j.jmb.2017.04.015)

20 Fernandez-Moya, S. M., Carrington, M., Estevez, A. M. 2014 Depletion of the RNA-binding protein RBP33 results in increased expression of silenced RNA polymerase II transcripts in Trypanosoma brucei. *PLoS One*. **9**, e107608. (10.1371/journal.pone.0107608)

21 Cirovic, O., Trikin, R., Hoffmann, A., Doiron, N., Jakob, M., Ochsenreiter, T. 2017 The nuclear RNA binding protein RBP33 influences mRNA and spliced leader RNA abundance in Trypanosoma brucei. *Molecular and biochemical parasitology*. **212**, 16-20. (10.1016/j.molbiopara.2016.12.008)

22 Michels, P. A., Bernards, A., Van der Ploeg, L. H., Borst, P. 1982 Characterization of the expression-linked gene copies of variant surface glycoprotein 118 in two independently isolated clones of Trypanosoma brucei. *Nucleic acids research*. **10**, 2353-2366.

23 De Lange, T., Michels, P. A., Veerman, H. J., Cornelissen, A. W., Borst, P. 1984 Many trypanosome messenger RNAs share a common 5' terminal sequence. *Nucleic acids research*. **12**, 3777-3790.

24 Kooter, J., Borst, P. 1984 Alpha-amanitin-insensitive transcription of variant surface glycoprotein genes provides further evidence for discontinuous transcription in trypanosomes. *Nucleic Acids Res.* **12**, 9457-9472.

25 Guyaux, M., Cornelissen, A. W., Pays, E., Steinert, M., Borst, P. 1985 Trypanosoma brucei: a surface antigen mRNA is discontinuously transcribed from two distinct chromosomes. *Embo j*. **4**, 995-998.

26 Krause, M., Hirsh, D. 1987 A trans-spliced leader sequence on actin mRNA in C. elegans. *Cell*. **49**, 753-761.

27 Krchňáková, Z., Krajčovič, J., Vesteg, M. 2017 On the Possibility of an Early Evolutionary Origin for the Spliced Leader Trans-Splicing. *Journal of Molecular Evolution*. **in press**, (10.1007/s00239-017-9803-y)

28 Roy, S. W. 2017 Genomic and Transcriptomic Analysis Reveals Spliced Leader Trans-Splicing in Cryptomonads. *Genome Biol Evol*. **9**, 468-473. (10.1093/gbe/evx012)

29 Matsuo, M., Katahata, A., Satoh, S., Matsuzaki, M., Nomura, M., Ishida, K. I., Inagaki, Y., Obokata, J. 2018 Characterization of spliced leader trans-splicing in a photosynthetic rhizarian amoeba, Paulinella micropora, and its possible role in functional gene transfer. *PLoS One*. **13**, e0200961. (10.1371/journal.pone.0200961)

30 Siegel, T., Tan, K., Cross, G. 2005 Systematic study of sequence motifs for RNA *trans* splicing in *Trypanosoma brucei*. *Mol. Cell Biol.* **25**, 9586–9594.

31 Matthews, K. R., Tschudi, C., Ullu, E. 1994 A common pyrimidine-rich motif governs *trans*-splicing and polyadenylation of tubulin polycistronic pre-mRNA in trypanosomes. *Genes Dev.* **8**, 491-501.

32 Kolev, N., Franklin, J., Carmi, S., Shi, H., Michaeli, S., Tschudi, C. 2010 The transcriptome of the human pathogen *Trypanosoma brucei* at single-nucleotide resolution. *PLoS pathogens*. **6**, e1001090.

33 Siegel, T., Hekstra, D., Wang, X., Dewell, S., Cross, G. 2010 Genome-wide analysis of mRNA abundance in two life-cycle stages of *Trypanosoma brucei* and identification of splicing and polyadenylation sites. *Nucleic acids research*. **38**, 4946-4957.

34 Smith, M., Blanchette, M., Papadopoulou, B. 2008 Improving the prediction of mRNA extremities in the parasitic protozoan Leishmania. *BMC Bioinformatics*. **9**, 158. (10.1186/1471-2105-9-158)

35 Hummel, H. S., Gillespie, R. D., Swindle, J. 2000 Mutational analysis of 3' splice site selection during trans-splicing. *The Journal of biological chemistry*. **275**, 35522-35531. (10.1074/jbc.M002424200)

36 Patzelt, E., Perry, K. L., Agabian, N. 1989 Mapping of branch sites in trans-spliced pre-mRNAs of Trypanosoma brucei. *Mol Cell Biol*. **9**, 4291-4297.

37 Lücke, S., Jürchott, K., Hung, L.-H., Bindereif, A. 2005 mRNA splicing in Trypanosoma brucei: Branch-point mapping reveals differences from the canonical U2 snRNA-mediated recognition. *Mol Biochem Parasitol.* **142**, 248-251.

38 Dillon, L. A., Okrah, K., Hughitt, V. K., Suresh, R., Li, Y., Fernandes, M. C., Belew, A. T., Corrada Bravo, H., Mosser, D. M., El-Sayed, N. M. 2015 Transcriptomic profiling of gene expression and RNA processing during Leishmania major differentiation. *Nucleic acids research*. (10.1093/nar/gkv656)

39 Campos, P. C., Bartholomeu, D. C., DaRocha, W. D., Cerqueira, G. C., Teixeira, S. M. 2008 Sequences involved in mRNA processing in Trypanosoma cruzi. *Int J Parasitol*. **38**, 1383-1389.

40 Tkacz, I., Gupta, S., Volkov, V., Romano, M., Haham, T., Tulinski, P., Lebenthal, I., Michaeli, S., . 2010 Analysis of spliceosomal proteins in trypanosomatids reveals novel functions in mRNA processing. *The Journal of biological chemistry*. **285**, 27982-27999.

41 Wall, R. J., Rico, E., Lukac, I., Zuccotto, F., Elg, S., Gilbert, I. H., Freund, Y., Alley, M. R. K., Field, M. C., Wyllie, S.*, et al.* 2018 Clinical and veterinary trypanocidal benzoxaboroles target CPSF3. *Proceedings of the National Academy of Sciences of the United States of America*. **115**, 9616-9621. (10.1073/pnas.1807915115)

42 Jones, D. C., Foth, B. J., Urbaniak, M. D., Patterson, S., Ong, H. B., Berriman, M., Fairlamb, A. H. 2015 Genomic and Proteomic Studies on the Mode of Action of Oxaboroles against the African Trypanosome. *PLoS neglected tropical diseases*. **9**, e0004299. (10.1371/journal.pntd.0004299)

43 Begolo, D., Vincent, I., Giordani, F., Pöhner, I., Witty, M., Rowan, T., Bengaly, Z., Gillingwater, K., Freund, Y., Wade, R.*, et al.* 2018 The trypanocidal benzoxaborole AN7973 inhibits trypanosome mRNA processing. *PLoS pathogens*. **14**, e1007315. . (https://doi.org/10.1371/journal.ppat.1007315)

44 David, C. J., Boyne, A. R., Millhouse, S. R., Manley, J. L. 2011 The RNA polymerase II C-terminal domain promotes splicing activation through recruitment of a U2AF65-Prp19 complex. *Genes Dev*. **25**, 972-983. (10.1101/gad.2038011)

45 Saldi, T., Cortazar, M. A., Sheridan, R. M., Bentley, D. L. 2016 Coupling of RNA Polymerase II Transcription Elongation with Pre-mRNA Splicing. *J Mol Biol*. **428**, 2623-2635. (10.1016/j.jmb.2016.04.017)

46 Nojima, T., Gomes, T., Carmo-Fonseca, M., Proudfoot, N. J. 2016 Mammalian NET-seq analysis defines nascent RNA profiles and associated RNA processing genome-wide. *Nat Protoc*. **11**, 413-428. (10.1038/nprot.2016.012)

47 Neves, L. T., Douglass, S., Spreafico, R., Venkataramanan, S., Kress, T. L., Johnson, T. L. 2017 The histone variant H2A.Z promotes efficient cotranscriptional splicing in S. cerevisiae. *Genes Dev*. **31**, 702-717. (10.1101/gad.295188.116)

48 Chathoth, K. T., Barrass, J. D., Webb, S., Beggs, J. D. 2014 A splicing-dependent transcriptional checkpoint associated with prespliceosome formation. *Molecular cell*. **53**, 779-790. (10.1016/j.molcel.2014.01.017)

49 Alpert, T., Herzel, L., Neugebauer, K. M. 2017 Perfect timing: splicing and transcription rates in living cells. *Wiley interdisciplinary reviews. RNA*. **8**, (10.1002/wrna.1401)

50 Maree, J. P., Patterton, H. G. 2014 The epigenome of Trypanosoma brucei: a regulatory interface to an unconventional transcriptional machine. *Biochim Biophys Acta*. **1839**, 743-750. (10.1016/j.bbagrm.2014.05.028)

51 Briggs, E., Hamilton, G., Crouch, K., Lapsley, C., McCulloch, R. 2018 Genome-wide mapping reveals conserved and diverged R-loop activities in the unusual genetic landscape of the African trypanosome genome. *Nucleic acids research*. (10.1093/nar/gky928)

52 Lombrana, R., Alvarez, A., Fernandez-Justel, J. M., Almeida, R., Poza-Carrion, C., Gomes, F., Calzada, A., Requena, J. M., Gomez, M. 2016 Transcriptionally Driven DNA Replication Program of the Human Parasite *Leishmania major*. *Cell Rep*. **16**, 1774-1786. (10.1016/j.celrep.2016.07.007)

53 Gupta, S. K., Carmi, S., Waldman Ben-Asher, H., Tkacz, I. D., Naboishchikov, I., Michaeli, S. 2013 Basal splicing factors regulate the stability of mature mRNAs in trypanosomes. *The Journal of biological chemistry*. **288**, 4991-5006. (10.1074/jbc.M112.416578)

54 Mulindwa, J., Leiss, K., Ibberson, D., Kamanyi Marucha, K., Helbig, C., Melo do Nascimento, L., Silvester, E., Matthews, K., Matovu, E., Enyaru, J.*, et al.* 2018 Transcriptomes of *Trypanosoma brucei rhodesiense* from sleeping sickness patients, rodents and culture: Effects of strain, growth conditions and RNA preparation methods. *PLoS neglected tropical diseases*. **12**, e0006280. (10.1371/journal.pntd.0006280)

55 Chan, L. Y., Mugler, C. F., Heinrich, S., Vallotton, P., Weis, K. 2018 Non-invasive measurement of mRNA decay reveals translation initiation as the major determinant of mRNA stability. *Elife*. **7**, (10.7554/eLife.32536)

56 Wasmuth, E. V., Lima, C. D. 2012 Exo- and endoribonucleolytic activities of yeast cytoplasmic and nuclear RNA exosomes are dependent on the noncatalytic core and central channel. *Molecular cell*. **48**, 133-144. (10.1016/j.molcel.2012.07.012)

57 Makino, D. L., Schuch, B., Stegmann, E., Baumgartner, M., Basquin, C., Conti, E. 2015 RNA degradation paths in a 12-subunit nuclear exosome complex. *Nature*. **524**, 54-58. (10.1038/nature14865)

58 Makino, D. L., Baumgartner, M., Conti, E. 2013 Crystal structure of an RNA-bound 11-subunit eukaryotic exosome complex. *Nature*. **495**, 70-75. (10.1038/nature11870)

59 Schuller, J. M., Falk, S., Fromm, L., Hurt, E., Conti, E. 2018 Structure of the nuclear exosome captured on a maturing preribosome. *Science*. **360**, 219-222. (10.1126/science.aar5428)

60 Estévez, A., Kempf, T., Clayton, C. E. 2001 The exosome of *Trypanosoma brucei*. *EMBO J.* **20**, 3831-3839.

61 Estévez, A. M., Lehner, B., Sanderson, C. M., Ruppert, T., Clayton, C. 2003 The roles of inter-subunit interactions in exosome stability. *J. Biol. Chem.* **278**, 34943-34951.

62 Haile, S., Cristodero, M., Clayton, C., Estévez, A. 2007 The subcellular localisation of trypanosome RRP6 and its association with the exosome. *Molecular and biochemical parasitology*. **151**, 52-58. (10.1016/j.molbiopara.2006.10.005)

63 Kramer, S., Piper, S., Estevez, A., Carrington, M. 2016 Polycistronic trypanosome mRNAs are a target for the exosome. *Molecular and biochemical parasitology*. **205**, 1-5. (10.1016/j.molbiopara.2016.02.009)

64 Cesaro, G., Carneiro, F. R. G., Avila, A. R., Zanchin, N. I. T., Guimaraes, B. G. 2018 Trypanosoma brucei RRP44 is involved in an early stage of large ribosomal subunit RNA maturation. *RNA biology*. (10.1080/15476286.2018.1564463)

65 Seebeck, T., Whittaker, P. A., Imboden, M. A., Hardman, N., Braun, R. 1983 Tubulin genes of Trypanosoma brucei: a tightly clustered family of alternating genes. *Proceedings of the National Academy of Sciences of the United States of America*. **80**, 4634-4638.

66 Thomashow, L. S., Milhausen, M., Rutter, W. J., Agabian, N. 1983 Tubulin genes are tandemly linked and clustered in the genome of trypanosoma brucei. *Cell*. **32**, 35-43.

67 Muhich, M. L., Hsu, M. P., Boothroyd, J. C. 1989 Heat shock disruption of *trans* splicing in trypanosomes: effect on Hsp70, Hsp85 and tubulin mRNA synthesis. *Gene*. **82**, 169-175.

68 Kramer, S., Marnef, A., Standart, N., Carrington, M. 2012 Inhibition of mRNA maturation in trypanosomes causes the formation of novel foci at the nuclear periphery containing cytoplasmic regulators of mRNA fate. *J Cell Sci*. **125**, 2896-2909. (10.1242/jcs.099275)

69 Ullu, E., Matthews, K. R., Tschudi, C. 1993 Temporal order of RNA-processing reactions in trypanosomes: rapid *trans* splicing precedes polyadenylation of newly synthesized tubulin transcripts. *Mol. Cell. Biol.* **13**, 720-725.

70 Fadda, A., Färber, V., Droll, D., Clayton, C. 2013 The roles of 3'-exoribonucleases and the exosome in trypanosome mRNA degradation. *RNA (New York, N.Y.)*. **19**, 937-947.

71 Stern, M., Gupta, S., Salmon-Divon, M., Haham, T., Barda, O., Levi, S., Wachtel, C., Nilsen, T., Michaeli, S. 2009 Multiple roles for polypyrimidine tract binding (PTB) proteins in trypanosome RNA metabolism. *RNA (New York, N.Y.)*. **15**, 648-665.

72 Florini, F., Naguleswaran, A., Gharib, W. H., Bringaud, F., Roditi, I. 2018 Unexpected diversity in eukaryotic transcription revealed by the retrotransposon hotspot family of *Trypanosoma brucei*. *Nucleic acids research*. (10.1093/nar/gky1255)

73 Li, H., Tschudi, C. 2005 Novel and essential subunits in the 300-kilodalton nuclear cap binding complex of *Trypanosoma brucei*. *Mol Cell Biol*. **25**, 2216-2226. (10.1128/MCB.25.6.2216-2226.2005)

74 Bannerman, B. P., Kramer, S., Dorrell, R. G., Carrington, M. 2018 Multispecies reconstructions uncover widespread conservation, and lineage-specific elaborations in eukaryotic mRNA metabolism. *PLoS One*. **13**, e0192633. (10.1371/journal.pone.0192633)

75 Bercovich, N., Clayton, C., Levin, M., Vazquez, M. 2009 Identification of core components of the exon junction complex in trypanosomes. *Molecular and biochemical parasitology*. **166**, 190-193.

76 Dhalia, R., Marinsek, N., Reis, C., Katz, R., Muniz, J., Standart, N., Carrington, M., de Melo Neto, O. 2006 The two eIF4A helicases in Trypanosoma brucei are functionally distinct. *Nucleic Acids Res.* **34**, 2495-2507.

77 Inoue, A. H., Serpeloni, M., Hiraiwa, P. M., Yamada-Ogatta, S. F., Muniz, J. R., Motta, M. C., Vidal, N. M., Goldenberg, S., Avila, A. R. 2014 Identification of a novel nucleocytoplasmic shuttling RNA helicase of trypanosomes. *PLoS One*. **9**, e109521. (10.1371/journal.pone.0109521)

78 Archer, S., hirokikh, N., Hallwirth, C., Beilharz, T., Preiss, T. 2015 Probing the closed-loop model of mRNA translation in living cells. *RNA biology*. **12**, 248-254.

79 Goos, C., Dejung, M., Wehman, A., M-Natus, E., Schmidt, J., Sunter, J., Engstler, M., Butter, F., Kramer, S. 2018 Trypanosomes can initiate nuclear export co-transcriptionally *Nucleic acids research*. (10.1093/nar/gky1136)

80 Khong, A., Parker, R. 2018 mRNP architecture in translating and stress conditions reveals an ordered pathway of mRNP compaction. *J Cell Biol*. **217**, 4124-4140. (10.1083/jcb.201806183)

81 Coller, J., Wickens, M. 2007 Tethered function assays: an adaptable approach to study RNA regulatory proteins. *Methods Enzymol*. **429**, 299-321. (10.1016/s0076-6879(07)29014-7)

82 Erben, E., Fadda, A., Lueong, S., Hoheisel, J., Clayton, C. 2014 Genome-wide discovery of post-transcriptional regulators in *Trypanosoma brucei*. *PLoS pathogens*. **10**, e1004178. (doi:10.1371/journal.ppat.1004178)

83 Lueong, S., Merce, C., Fischer, B., Hoheisel, J., Erben, E. 2016 Gene expression regulatory networks in *Trypanosoma brucei*: insights into the role of the mRNA-binding proteome. *Molecular microbiology*. **100**, 457-471.

84 Li, K., Zhou, S., Guo, Q., Chen, X., Lai, D., Lun, Z., Guo, X. 2017 The eIF3 complex of *Trypanosoma brucei*: composition conservation does not imply the conservation of structural assembly and subunits function. *RNA*. **23**, 333-345 (10.1261/rna.058651.116)

85 Peter, D., Weber, R., Sandmeir, F., Wohlbold, L., Helms, S., Bawankar, P., Valkov, E., Igreja, C., Izaurralde, E. 2017 GIGYF1/2 proteins use auxiliary sequences to selectively bind to 4EHP and repress target mRNA expression. *Genes Dev*. **31**, 1147-1161. (10.1101/gad.299420.117)

86 Terrao, M., Kamanyi Marucha, K., Mugo, E., Droll, D., Minia, I., Egler, F., Braun, J., Clayton, C. 2018 The suppressive cap-binding-complex factor 4EIP is required for normal differentiation. *Nucleic Acids Res.* **46**, 8993-9010. (10.1093/nar/gky733)

87 Zinoviev, A., Leger, M., Wagner, G., Shapira, M. 2011 A novel 4E-interacting protein in *Leishmania* is involved in stage-specific translation pathways. *Nucleic acids research*. **39**, 8404-8415. (gkr555 [pii]

10.1093/nar/gkr555 [doi])

88 Vasquez, J. J., Hon, C. C., Vanselow, J. T., Schlosser, A., Siegel, T. N. 2014 Comparative ribosome profiling reveals extensive translational complexity in different *Trypanosoma brucei* life cycle stages. *Nucleic acids research*. **42**, 3623-3637. (10.1093/nar/gkt1386)

89 Fadda, A., Ryten, M., Droll, D., Rojas, F., Färber, V., Haanstra, J., Bakker, B., Matthews, K., Clayton, C. 2014 Transcriptome-wide analysis of mRNA decay reveals complex degradation kinetics and suggests a role for co-transcriptional degradation in determining mRNA levels. *Molecular microbiology*. **94**, 307-326. (10.1111/mmi.12764)

90 Woodward, L. A., Mabin, J. W., Gangras, P., Singh, G. 2017 The exon junction complex: a lifelong guardian of mRNA fate. *Wiley interdisciplinary reviews. RNA*. **8**, (10.1002/wrna.1411)

91 Hurt, J. A., Robertson, A. D., Burge, C. B. 2013 Global analyses of UPF1 binding and function reveal expanded scope of nonsense-mediated mRNA decay. *Genome Res.* **23**, 1636-1650. (10.1101/gr.157354.113)

92 Shoemaker, C. J., Green, R. 2012 Translation drives mRNA quality control. *Nat Struct Mol Biol*. **19**, 594-601. (10.1038/nsmb.2301)

93 Boehm, V., Haberman, N., Ottens, F., Ule, J., Gehring, N. H. 2014 3' UTR length and messenger ribonucleoprotein composition determine endocleavage efficiencies at termination codons. *Cell Rep*. **9**, 555-568. (10.1016/j.celrep.2014.09.012)

94 Zahdeh, F., Carmel, L. 2016 The role of nucleotide composition in premature termination codon recognition. *BMC Bioinformatics*. **17**, 519. (10.1186/s12859-016-1384-z)

95 Colombo, M., Karousis, E. D., Bourquin, J., Bruggmann, R., Muhlemann, O. 2017 Transcriptome-wide identification of NMD-targeted human mRNAs reveals extensive redundancy between SMG6- and SMG7-mediated degradation pathways. *RNA (New York, N.Y.)*. **23**, 189-201. (10.1261/rna.059055.116)

96 Delhi, P., Queiroz, R., Inchaustegui, D., Carrington, M., Clayton, C. 2011 Is there a classical nonsense-mediated decay pathway in trypanosomes? *PLoSOne*. **6**, e25112. (10.1371/journal.pone.0025112)

97 Schwede, A., Ellis, L., Luther, J., Carrington, M., Stoecklin, G., Clayton, C. 2008 A role for Caf1 in mRNA deadenylation and decay in trypanosomes and human cells. *Nucleic acids research*. **36**, 3374-3388. (10.1093/nar/gkn108)

98 Färber, V., Erben, E., Sharma, S., Stoecklin, G., Clayton, C. 2013 Trypanosome CNOT10 is essential for the integrity of the NOT deadenylase complex and for degradation of many mRNAs. *Nucleic acids research*. **41**, 1211-1222.

99 Irmer, H., Clayton, C. E. 2001 Degradation of the *EP1* mRNA in *Trypanosoma brucei* is initiated by destruction of the 3'-untranslated region. *Nucleic Acids Res.* **29**, 4707-4715.

100 Schwede, A., Manful, T., Jha, B., Helbig, C., Bercovich, N., Stewart, M., Clayton, C. 2009 The role of deadenylation in the degradation of unstable mRNAs in trypanosomes. *Nucleic acids research*. **37**, 5511-5528. (10.1093/nar/gkp571)

101 Haile, S., Estévez, A. M., Clayton, C. 2003 A role for the exosome in the initiation of degradation of unstable mRNAs. *RNA (New York, N.Y.)*. **9**, 1491-1501.

102 Deneke, C., Lipowsky, R., Valleriani, A. 2013 Complex degradation processes lead to non-exponential decay patterns and age-dependent decay rates of messenger RNA. *PLoS ONE*. **8**, e55442.

103 Ignatochkina, A. V., Takagi, Y., Liu, Y., Nagata, K., Ho, C. K. 2015 The messenger RNA decapping and recapping pathway in Trypanosoma. *Proceedings of the National Academy of Sciences of the United States of America*. **112**, 6967-6972. (10.1073/pnas.1424909112)

104 Kramer, S. 2017 The ApaH-like phosphatase TbALPH1 is the major mRNA decapping enzyme of trypanosomes. *PLoS pathogens*. **13**, e1006456. (10.1371/journal.ppat.1006456)

105 Li, C.-H., Irmer, H., Gudjonsdottir-Planck, D., Freese, S., Salm, H., Haile, S., Estévez, A. M., Clayton, C. E. 2006 Roles of a *Trypanosoma brucei* 5'->3' exoribonuclease homologue in mRNA degradation. *RNA (New York, N.Y.)*. **12**, 2171-2186. (10.1261/rna.291506)

106 Chavez, S., Eastman, G., Smircich, P., Becco, L. L., Oliveira-Rizzo, C., Fort, R., Potenza, M., Garat, B., Sotelo-Silveira, J. R., Duhagon, M. A. 2017 Transcriptome-wide analysis of the Trypanosoma cruzi proliferative cycle identifies the periodically expressed mRNAs and their multiple levels of control. *PLoS One*. **12**, e0188441. (10.1371/journal.pone.0188441)

107 Handa, N., Nureki, O., Kurimoto, K., Kim, I., Sakamoto, H., Shimura, Y., Muto, Y., Yokoyama, S. 1999 Structural basis for recognition of the tra mRNA precursor by the Sex-lethal protein. *Nature*. **398**, 579-585.

108 Wang, X., Tanaka Hall, T. 2001 Structural basis for recognition of AU-rich element RNA by the HuD protein. *Nat Struct Biol*. **8**, 141-145.

109 Hudson, B., Martinez-Yamout, M., Dyson, H., Wright, P. 2004 Recognition of the mRNA AU-rich element by the zinc finger domain of TIS11d. *Nat Struct Mol Biol*. **11**, 257–264.

110 Wang, X., Zamore, P. D., Hall, T. M. 2001 Crystal structure of a Pumilio homology domain. *Molecular cell*. **7**, 855-865.

111 Quenault, T., Lithgow, T., Traven, A. 2011 PUF proteins: repression, activation and mRNA localization. *Trends Cell Biol*. **21**, 104-112. (10.1016/j.tcb.2010.09.013)

112 Miller, M., Olivas, W. 2011 Roles of Puf proteins in mRNA degradation and translation. *WIREs RNA*. **2**, 471-492.

113 Batram, C., Jones, N. G., Janzen, C. J., Markert, S. M., Engstler, M. 2014 Expression site attenuation mechanistically links antigenic variation and development in Trypanosoma brucei. *Elife (Cambridge)*. **3**, e02324. (10.7554/eLife.02324)

114 Schulz, D., Mugnier, M. R., Paulsen, E. M., Kim, H. S., Chung, C. W., Tough, D. F., Rioja, I., Prinjha, R. K., Papavasiliou, F. N., Debler, E. W. 2015 Bromodomain Proteins Contribute to Maintenance of Bloodstream Form Stage Identity in the African Trypanosome. *PLoS Biol*. **13**, e1002316. (10.1371/journal.pbio.1002316)

115 McDonald, L., Cayla, M., Ivens, A., Mony, B. M., MacGregor, P., Silvester, E., McWilliam, K., Matthews, K. R. 2018 Non-linear hierarchy of the quorum sensing signalling pathway in bloodstream form African trypanosomes. *PLoS pathogens*. **14**, e1007145. (10.1371/journal.ppat.1007145)

116 Qiu, Y., Milanes, J. E., Jones, J. A., Noorai, R. E., Shankar, V., Morris, J. C. 2018 Glucose Signaling Is Important for Nutrient Adaptation during Differentiation of Pleomorphic African Trypanosomes. *mSphere*. **3**, (10.1128/mSphere.00366-18)

117 Naguleswaran, A., Doiron, N., Roditi, I. 2018 RNA-Seq analysis validates the use of culture-derived Trypanosoma brucei and provides new markers for mammalian and insect life-cycle stages. *BMC genomics*. **19**, 227. (10.1186/s12864-018-4600-6)

118 Silvester, E., Ivens, A., Matthews, K. R. 2018 A gene expression comparison of *Trypanosoma brucei* and *Trypanosoma congolense* in the bloodstream of the mammalian host reveals species-specific adaptations to density-dependent development. *PLoS neglected tropical diseases*. **12**, e0006863. (10.1371/journal.pntd.0006863)

119 Minia, I., Merce, C., Terrao, M., Clayton, C. 2016 Translation regulation and RNA granule formation after heat shock of procyclic form *Trypanosoma brucei*: many heat-induced mRNAs are increased during differentiation to mammalian-infective forms. *PLoS neglected tropical diseases*. **10**, e0004982.

120 Patel, M. M., Volkov, O. A., Leija, C., Lemoff, A., Phillips, M. A. 2018 A dual regulatory circuit consisting of S-adenosylmethionine decarboxylase protein and its reaction product controls expression of the paralogous activator prozyme in Trypanosoma brucei. *PLoS pathogens*. **14**, e1007404. (10.1371/journal.ppat.1007404)

121 Fernandez-Moya, S. M., Carrington, M., Estevez, A. M. 2014 A short RNA stem-loop is necessary and sufficient for repression of gene expression during early logarithmic phase in trypanosomes. *Nucleic acids research*. **42**, 7201-7209. (10.1093/nar/gku358)

122 Alves, L., Oliveira, C., Morking, P., Kessler, R., Martins, S., Romagnoli, B., Marchini, F., Goldenberg, S. 2014 The mRNAs associated to a zinc finger protein from Trypanosoma cruzi shift during stress conditions. *RNA biology*. **11**, (10.4161/rna.29622)

123 Miguel De Pablos, L., Kelly, S., Nascimento, J., Sunter, J., Carrington, M. 2017 Characterization of RBP9 and RBP10, two developmentally regulated RNA binding proteins in *Trypanosoma brucei*. *Open Biol*. **7**, 160159.

124 Mugo, E., Clayton, C. 2017 Expression of the RNA-binding protein RBP10 promotes the bloodstream-form differentiation state in *Trypanosoma brucei*. *PLoS pathogens*. **13**, e1006560.

125 Wippel, H. H., Inoue, A. H., Vidal, N. M., Costa, J. F. D., Marcon, B. H., Romagnoli, B. A. A., Santos, M. D. M., Carvalho, P. C., Goldenberg, S., Alves, L. R. 2018 Assessing the partners of RBP9-mRNP complex in *Trypanosoma cruzi* using shotgun proteomics and RNA-seq. *RNA biology*. (10.1080/15476286.2018.1509660)

126 Hendriks, E. F., Matthews, K. R. 2005 Disruption of the developmental programme of *Trypanosoma brucei* by genetic ablation of TbZFP1, a differentiation-enriched CCCH protein. *Molecular microbiology*. **57**, 706-716.

127 Hendriks, E. F., Robinson, D. R., Hinkins, M., Matthews, K. R. 2001 A novel CCCH protein which modulates differentiation of *Trypanosoma brucei* to its procyclic form. *EMBO J.* **20**, 6700-6711.

128 Paterou, A., Walrad, P., Craddy, P., Fenn, K., Matthews, K. 2006 Identification and stage-specific association with the translational apparatus of TbZFP3, a ccch protein that promotes trypanosome life cycle development. *The Journal of biological chemistry*. **281**, 39002-39013.

129 Walrad, P., Paterou, A., Acosta-Serrano, A., Matthews, K. 2009 Differential trypanosome surface coat regulation by a CCCH protein that co-associates with procyclin mRNA cis-elements. *PLoS Pathog.* **5**, e1000317.

130 Walrad, P., Capewell, P., Fenn, K., Matthews, K. 2011 The post-transcriptional trans-acting regulator, TbZFP3, co-ordinates transmission-stage enriched mRNAs in *Trypanosoma brucei*. *Nucleic acids research*. **40**, 2869-2883. (10.1093/nar/gkr1106)

131 Vassella, E., Den Abbeele, J., Butikofer, P., Renggli, C. K., Furger, A., Brun, R., Roditi, I. 2000 A major surface glycoprotein of *Trypanosoma brucei* is expressed transiently during development and can be regulated post-transcriptionally by glycerol or hypoxia. *Genes Dev.* **14**, 615-626.

132 Vassella, E., Probst, M., Schneider, A., Studer, E., Renggli, C., Roditi, I. 2004 Expression of a major surface protein of *Trypanosoma brucei* insect forms is controlled by the activity of mitochondrial enzymes. *Mol Biol Cell.* **15**, 3986-3993.

133 Walker, R., Jr., Saha, L., Hill, G. C., Chaudhuri, M. 2005 The effect of over-expression of the alternative oxidase in the procyclic forms of Trypanosoma brucei. *Molecular and biochemical parasitology*. **139**, 153-162. (10.1016/j.molbiopara.2004.11.003)

134 Dean, S., Sunter, J., Wheeler, R. 2016 TrypTag.org: A trypanosome genome-wide protein localisation resource. *Trends Parasitol.* **33**, 80-82.

135 Najafabadi, H. S., Lu, Z., MacPherson, C., Mehta, V., Adoue, V., Pastinen, T., Salavati, R. 2013 Global identification of conserved post-transcriptional regulatory programs in trypanosomatids. *Nucleic acids research*. **41**, 8591-8600. (10.1093/nar/gkt647)

136 Jha, B., Archer, S., Clayton, C. 2013 The trypanosome pumilio domain protein PUF5. *PloS ONE*. **8**, e77371. (10.1371/journal.pone.0077371)

137 Jha, B. A., Gazestani, V. H., Yip, C. W., Salavati, R. 2015 The DRBD13 RNA binding protein is involved in the insect-stage differentiation process of Trypanosoma brucei. *FEBS Lett.* **589**, 1966-1974. (10.1016/j.febslet.2015.05.036)

138 Hartmann, C., Clayton, C. 2008 Regulation of a transmembrane protein gene family by the small RNA binding proteins TbUBP1 and TbUBP2. *Molecular and biochemical parasitology*. **157**, 112-115.

139 De Gaudenzi, J. G., D'Orso, I., Frasch, A. C. 2003 RNA recognition motif-type RNA-binding proteins in Trypanosoma cruzi form a family involved in the interaction with specific transcripts in vivo. *The Journal of biological chemistry*. **278**, 18884-18894. (10.1074/jbc.M301756200)

140 Noe, G., De Gaudenzi, J., Frasch, A. 2008 Functionally related transcripts have common RNA motifs for specific RNA-binding proteins in trypanosomes. *BMC Mol. Biol.* **9**, 107.

141 Wurst, M., Robles, A., Po, J., Luu, V., Brems, S., Marentije, M., Stoitsova, S., Quijada, L., Hoheisel, J., Stewart, M.*, et al.* 2009 An RNAi screen of the RRM-domain proteins of *Trypanosoma brucei*. *Molecular and biochemical parasitology*. **163**, 61-65. (DOI: 10.1016/j.molbiopara.2008.09.001)

142 Robles, A., Clayton, C. 2008 Regulation of an amino acid transporter mRNA in *Trypanosoma brucei*. *Molecular and biochemical parasitology*. **157**, 102-106.

143 Das, A., Bellofatto, V., Rosenfeld, J., Carrington, M., Romero-Zaliz, R., del Val, C., Estevez, A. M. 2015 High throughput sequencing analysis of Trypanosoma brucei DRBD3/PTB1-bound mRNAs. *Molecular and biochemical parasitology*. **199**, 1-4. (10.1016/j.molbiopara.2015.02.003)

144 Reijns, M. A., Alexander, R. D., Spiller, M. P., Beggs, J. D. 2008 A role for Q/N-rich aggregation-prone regions in P-body localization. *J Cell Sci*. **121**, 2463-2472.

145 Zhang, H., Elbaum-Garfinkle, S., Langdon, E. M., Taylor, N., Occhipinti, P., Bridges, A. A., Brangwynne, C. P., Gladfelter, A. S. 2015 RNA Controls PolyQ Protein Phase Transitions. *Molecular cell*. **60**, 220-230. (10.1016/j.molcel.2015.09.017)

146 Nott, T. J., Petsalaki, E., Farber, P., Jervis, D., Fussner, E., Plochowietz, A., Craggs, T. D., Bazett-Jones, D. P., Pawson, T., Forman-Kay, J. D.*, et al.* 2015 Phase transition of a disordered nuage protein

generates environmentally responsive membraneless organelles. *Molecular cell*. **57**, 936-947. (10.1016/j.molcel.2015.01.013)

147 Nissan, T., Rajyaguru, P., She, M., Song, H., Parker, R. 2010 Decapping activators in *Saccharomyces cerevisiae* act by multiple mechanisms. *Molecular cell*. **39**, 773-783.

148 Rajyaguru, P., She, M., Parker, R. 2012 Scd6 targets eIF4G to repress translation: RGG motif proteins as a class of eIF4G-binding proteins. *Molecular cell*. **45**, 244-254. (10.1016/j.molcel.2011.11.026)

149 Poornima, G., Shah, S., Vignesh, V., Parker, R., Rajyaguru, P. I. 2016 Arginine methylation promotes translation repression activity of eIF4G-binding protein, Scd6. *Nucleic acids research*. **44**, 9358-9368. (10.1093/nar/gkw762)

150 Holetz, F., Correa, A., Avila, A., Nakamura, C., Krieger, M., Goldenberg, S. 2007 Evidence of P-body-like structures in *Trypanosoma cruzi*. *Biochem. Biophys. Res. Commun.* **356**, 1062-1067.

151 Webb, H., Burns, R., Ellis, L., Kimblin, N., Carrington, M. 2005 Developmentally regulated instability of the GPI-PLC mRNA is dependent on a short-lived protein factor. *Nucleic Acids Res.* **33**, 1503-1512.

152 Glass, D., Polvere, R., Van der Ploeg, L. 1986 Conserved sequences and transcription of the hsp70 gene family in *Trypanosoma brucei*. *Mol Cell Biol*. **6**, 4657-4666.

153 Muhich, M., Boothroyd, J. C. 1988 Polycistronic transcripts in trypanosomes and their accumulation during heat shock: evidence for a precursor role in mRNA synthesis. *Mol. Cell. Biol.* **8**, 3837-3846.

154 Muhich, M. L., Boothroyd, J. C. 1989 Synthesis of trypanosome hsp70 mRNA is resistant to disruption of *trans*-splicing by heat shock. *J. Biol. Chem.* **264**, 7107-7110.

155 Kelly, S., Kramer, S., Schwede, A., Maini, P., Gull, K., Carrington, M. 2012 Genome organization is a major component of gene expression control in response to stress and during the cell division cycle in trypanosomes. *Open Biol*. **2**, 120033.

156 Lee, M. G.-S. 1995 Heat shock does not affect the transcriptional efficiency of the hsp 70 genes of *Trypanosoma brucei*. *Exp. Parasit.* **81**, 608-613.

157 Singh, A., Minia, I., Droll, D., Fadda, A., Clayton, C., Erben, E. 2014 Trypanosome MKT1 and the RNA-binding protein ZC3H11: interactions and potential roles in post-transcriptional regulatory networks. *Nucleic acids research*. **42**, 4652-4668. (10.1093/nar/gkt1416)

158 Kruger, T., Hofweber, M., Kramer, S. 2013 SCD6 induces RNP granule formation in trypanosomes in a translation-independent manner, regulated by its LSM and RGG domains. *Mol Biol Cell*. **24**, 2098-2111. (10.1091/mbc.E13-01-0068)

159 Antwi, E., Haanstra, J., Ramasamy, G., Jensen, B., Droll, D., Rojas, F., Minia, I., Terrao, M., Mercé, C., Matthews, K.*, et al.* 2016 Integrative analysis of the *Trypanosoma brucei* gene expression cascade predicts differential regulation of mRNA processing and unusual control of ribosomal protein expression. *BMC genomics*. **17**, 306. (10.1186/s12864-016-2624-3)

160 Jha, B., Fadda, A., Merce, C., Mugo, E., Droll, D., Clayton, C. 2014 Depletion of the trypanosome pumilio domain protein PUF2 causes transcriptome changes related to coding region length. *Eukaryot Cell*. **13**, 664-674. (doi: 10.1128/EC.00018-14)

161 Klein, C., Terrao, M., Clayton, C. 2017 The role of the zinc finger protein ZC3H32 in bloodstream-form *Trypanosoma brucei*. *PLoS ONE*. **12**, e0177901.

162 Chakraborty, C., Clayton, C. 2018 Stress susceptibility in Trypanosoma brucei lacking the RNA-binding protein ZC3H30. *PLoS neglected tropical diseases*. **12**, e0006835. (10.1371/journal.pntd.0006835)

163 Naguleswaran, A., Gunasekera, K., Schimanski, B., Heller, M., Hemphill, A., Ochsenreiter, T., Roditi, I. 2015 Trypanosoma brucei RRM1 is a nuclear RNA-binding protein and modulator of chromatin structure. *mBio*. **6**, e00114. (10.1128/mBio.00114-15)

164 Kafkova, L., Debler, E. W., Fisk, J. C., Jain, K., Clarke, S. G., Read, L. K. 2017 The major protein arginine methyltransferase in *Trypanosoma brucei* functions as an enzyme-prozyme complex. *The Journal of biological chemistry*. **292**, 2089-2100. (10.1074/jbc.M116.757112)

165 Kafkova, L., Tu, C., Pazzo, K. L., Smith, K. P., Debler, E. W., Paul, K. S., Qu, J., Read, L. K. 2018 *Trypanosoma brucei* PRMT1 Is a Nucleic Acid Binding Protein with a Role in Energy Metabolism and the Starvation Stress Response. *mBio*. **9**, (10.1128/mBio.02430-18)
